# Origin and Loss of Nested LRRTM*/*α-Catenin Genes during Vertebrate Evolution

**DOI:** 10.1371/journal.pone.0089910

**Published:** 2014-02-24

**Authors:** Pavel Uvarov, Tommi Kajander, Matti S. Airaksinen

**Affiliations:** 1 Institute of Biomedicine, Anatomy, University of Helsinki, Helsinki, Finland; 2 Institute of Biotechnology, University of Helsinki, Helsinki, Finland; University Zürich, Switzerland

## Abstract

Leucine-rich repeat transmembrane neuronal proteins (LRRTMs) form in mammals a family of four postsynaptic adhesion proteins, which have been shown to bind neurexins and heparan sulphate proteoglycan (HSPG) glypican on the presynaptic side. Mutations in the genes encoding LRRTMs and neurexins are implicated in human cognitive disorders such as schizophrenia and autism. Our analysis shows that in most jawed vertebrates, *lrrtm1, lrrtm2,* and *lrrtm3* genes are nested on opposite strands of large conserved intron of α-catenin genes *ctnna2, ctnna1,* and *ctnna3*, respectively. No *lrrtm* genes could be found in tunicates or lancelets, while two *lrrtm* genes are found in the lamprey genome, one of which is adjacent to a single *ctnna* homolog. Based on similar highly positive net charge of lamprey LRRTMs and the HSPG-binding LRRTM3 and LRRTM4 proteins, we speculate that the ancestral LRRTM might have bound HSPG before acquiring neurexins as binding partners. Our model suggests that *lrrtm* gene translocated into the large *ctnna* intron in early vertebrates, and that subsequent duplications resulted in three *lrrtm/ctnna* gene pairs present in most jawed vertebrates. However, we detected three prominent exceptions: (1) the *lrrtm3/ctnna3* gene structure is absent in the ray-finned fish genomes, (2) the genomes of clawed frogs contain *ctnna1* but lack the corresponding nested (*lrrtm2*) gene, and (3) contain *lrrtm3* gene in the syntenic position but lack the corresponding host (*ctnna3*) gene. We identified several other protein-coding nested gene structures of which either the host or the nested gene has presumably been lost in the frog or chicken lineages. Interestingly, majority of these nested genes comprise LRR domains.

## Introduction

Members of the leucine-rich repeat transmembrane (LRRTM) family of neuronal proteins contain ten N-terminal LRR repeats, a single pass transmembrane domain, and a C-terminal cytoplasmic tail comprising a PDZ domain binding motif [1]. LRRTMs function as postsynaptic adhesion proteins in excitatory synapses [2] by interacting with presynaptic neurexins, similar to the neuroligins [3]–[7]. *LRRTM1* gene is associated with schizophrenia and handedness [8]. In rodents, LRRTM1 and LRRTM2 proteins have been shown to interact with neurexins, but there are also indications that all the four LRRTMs can bind to neurexins [3]–[6]. Recently, heparan sulfate proteoglycan (HSPG) glypican was identified as an alternative receptor for LRRTM4 and possibly for LRRTM3 [9], [10].

In human and mouse genomes LRRTM1 is encoded by a single exon, whereas the first four coding nucleotides (ATGG) of other LRRTM genes (*Lrrtm2, Lrrtm3*, and *Lrrtm4*) are located in a separate first exon [1]. Three of the four LRRTM genes (*Lrrtm1* to *Lrrtm3*) are nested in a large conserved intron of α-catenin genes (*Ctnna1* to *Ctnna3*) [1]. Each mammalian *Ctnna* gene has 17 coding exons (encoding a protein of about 900 amino acids) and hosts one *Lrrtm* nested in the opposite orientation in a large (∼50–450 kb in human) intron between coding exons 6 and 7: *Lrrtm1* is nested in *Ctnna2*, *Lrrtm2* in *Ctnna1*, and *Lrrtm3* in *Ctnna3*. *Lrrtm4* gene is not nested but is located within a few genes away from the *Lrrtm1/Ctnna2* gene pair in mammals [1]. Genes encoding for α-catenins exist in all metazoan animals analyzed [11], whereas LRRTM genes have only been found in vertebrate genomes [1].

Nested genes represent a subgroup of overlapping genes [12]: one gene (“nested”) is situated totally inside another gene (“host”). Nearly all protein-coding nested genes are thought to have emerged by insertion of a corresponding DNA sequence into an intron of a pre-existing gene [13]. Most commonly, the internal/nested gene lies inside an intron of the larger host gene in the opposite orientation [12]. Nested genes that have a single coding exon presumably emerged by retrotransposition [13]. A gene may also become nested by fusion of two flanking genes or by acquisition of new exons. Alternatively, nested genes may originate *de novo* through accumulation of mutations inside a preexisting gene [12]. Once formed, a nested gene structure can be duplicated or lost during evolution. However, no loss of a nested gene structure encoding conserved proteins was reported in vertebrates in a previous study [13].

Here, we have studied the evolution of the LRRTM family. Our analysis suggests that in early vertebrates an ancestral *lrrtm* gene had become incorporated into a pre-existing *ctnna* intron that was followed by two duplications of the nested *lrrtm/ctnna* structure. We found that the nested *lrrtm/ctnna* gene structure is conserved in jawed vertebrates. However, the clawed frog (*Xenopus*) genome contains two notable exceptions. First, the genome contains *ctnna1* but lacks the corresponding nested (*lrrtm2*) gene. Second, the genome contains a clear *lrrtm3* ortholog in syntenic position but lacks the corresponding host (*ctnna3*) gene. A database analysis identified several other phylogenetically old nested gene structures comprising LRR-domain encoding genes that have apparently been lost in amphibian or avian lineages.

Although invertebrates, such as fruit fly and nematode have a neurexin (*nrxn*) gene ortholog [14], [15], the evolution of the alternatively spliced *nrxn* AS4 exon, which encodes a loop sequence required for LRRTM binding in mammals [3]–[6], has not been investigated. Therefore, we also studied whether the alternative splicing of *nrxn* AS4 exon would have co-evolved with the appearance of *lrrtm*. We show that the AS4 exon emerged *de novo* in chordates, and that the mechanism of its alternative splicing may have evolved in the early vertebrates. Based on analysis of net charge of the extracellular LRR domains, we speculate that the first LRRTMs may have bound HPSGs before acquiring neurexins as binding partners.

## Materials and Methods

### Identification of Sequences

We searched the Ensembl genome database (release 72, Jun 2013) for the genomic location and structure of the annotated LRRTM and α-catenin gene homologs (by searching for their names/gene symbols) from the following species: human, chicken (*Gallus gallus*), Western (tropical) clawed frog (*Xenopus tropicalis*), coelacanth (*Latimeria chalumnae*), zebrafish (*Danio rerio*), and sea lamprey (*Petromyzon marinus*). *Lrrtm* orthologs were also retrieved from other ray-finned fish genomes (*Gasterosteus aculeatus, Oryzias latipes, Takifugu rubripes,* and *Tetraodon nigroviridis*). In addition, we searched the tunicates (*Ciona intestinalis* and *Ciona savignyi*), amphioxus (*Branchiostoma floridae,* genome.jgi-psf.org/Brafl1), elephant shark (*Callorhinchus milii*, esharkgenome.imcb.a-star.edu.sg), spotted gar (*Lepisosteus oculatus*, pre.ensembl.org/Lepisosteus_oculatus), and the African clawed frog (*Xenopus laevis,* xenopus.lab.nig.ac.jp/assembly v7.1) genomes. We also searched the transcriptomes of clawed frogs (*X. laevis* and *X. tropicalis*, www.xenbase.org) and salamander (axolotl, *Ambystoma mexicanum*, www.ambystoma.org, assembly V4.0) for *lrrtm* and *ctnna* homologs. If some LRRTM or α-catenin homologs seemed to be missing or incompletely annotated, we searched the corresponding genomes by using TBLASTN (blast.ncbi.nlm.nih.gov/) using the corresponding mouse and chicken protein sequences as a query and verified the hits by reciprocal BLAST searches (using default parameters). The N-terminal part of some LRRTM transcripts was curated manually to conform to the splice site consensus sequences. Identified shark and coelacanth CTNNA fragments were aligned and assembled manually. Isoelectric point (pI) values were calculated using Geneious 6.1.7 (Biomatters Ltd.) for the extracellular LRR-domains of LRRTMs (excluding the signal sequence and hinge domain). These pI values and accession numbers for the identified LRRTM and α-catenin sequences are provided in Table S1.

### Analysis of Synteny

We identified human orthologs for genes surrounding the *lrrtm3* gene within *X. tropicalis* scaffold_7:33-34M (www.xenbase.org) and their chromosomal position in human genome using Ensembl. Presence of regions of conserved synteny (paralogous pairwise clusters) between the *CTNNA* gene regions within the human genome were analyzed using the Synteny Database (syntenydb.uoregon.edu/synteny_db/) using a sliding window size of 50 or 100 genes and *C. intestinalis* as outgroup [16]. Possible conserved synteny between vertebrate genomes (e.g. in regions containing the *lrrtm4* gene) was analyzed using the Genomicus database (v73 www.genomicus.biologie.ens.fr).

### Alignment and Phylogenetic Analysis

The predicted LRRTM and CTNNA amino acid sequences were aligned using MAFFT v.7 (http://mafft.cbrc.jp/alignment/software/) [17] with default parameters. For LRRTM3 and LRRTM4 orthologs that have alternative C-terminal splice forms, only the shorter isoform (ending to -ECEV) was used. The alignment was edited using Geneious in order to remove positions (amino acid residues) of the LRRTM signal sequence and the extracellular juxtamembrane domain where more than half of the sequences had gaps. The LRRTM alignment is shown in Fig. S1. Phylogenetic trees were inferred using PhyML3.0 under the following model parameters (LG substitution model, empirical equilibrium frequencies, four gamma-distributed substitution rate categories and five random starting trees) with confidence estimates derived from 1000 bootstrap replicates [18]. Trees were rearranged with Geneious and visualized using the MEGA5 software [19].

### Analysis of Selected Nested Gene Structures in Vertebrates

We also searched the Ensembl database for vertebrate orthologs for a subset of previously identified human different strand nested gene pairs [12]. We included for the search only those different strand nested gene pairs that were reported to be shared between human and mouse [12], and in which a protein-coding nested gene is flanked by protein-coding exons of the host gene. This selection resulted in 91 protein-coding different strand nested gene pairs for our analysis (Table S3). If annotated orthologs for the nested gene pair were identified (by searching for their names/gene symbols) in coelacanth or zebrafish (or in both), as well as in chicken and clawed frog (*X. tropicalis*) genomes, the nested structure was designated as conserved. If the nested gene structure was present in coelacanth or zebrafish but either the host or the nested gene, or both, were not annotated in either chicken or clawed frog genomes, the nested gene structure was designated as potentially lost (not conserved). The absence of these nested gene structures in chicken or in *X. tropicalis* genomes was verified by BLAST searches and by synteny analysis of adjacent genes.

### Evolution of *neurexin* AS4 Exon and Alternative Splicing

To study when the *nrxn* AS4 exon emerged during evolution, we searched selected invertebrate and vertebrate genomes with BLASTP using a 160 amino acid residue fragment of mouse neurexin-1 protein (ENSMUSP00000125407, Refseq NP_064648.3) that is encoded by the AS4 and flanking exons (see Fig. S6). To estimate the relative percentage of *nrxn* transcripts in which the AS4 exon is skipped or retained in selected species (that contain the AS4 exon), we searched the NCBI expressed sequence tag database (dbEST) with TBLASTN with default parameters (BLOSUM62 matrix) using the above 160 amino acid fragment of mouse neurexin-1 as a query (Fig. S6). Hits that were considered relevant for the analysis were at least 80 amino acid long, aligned at least partially with the AS4 exon of the query, and had over 30% sequence identity (Table S6). This ruled out short fragments and distant (non-neurexin) sequences. The location of AU-rich sequence motifs in the introns flanking the *nrxn* AS4 exon (within 200 bp upstream and 200 bp downstream of the exon) was analyzed by text search.

### Genomic PCR with Degenerate Primers

We purified *X. tropicalis* (obtained from the European Xenopus Resource Centre, www.port.ac.uk/research/exrc/) and chicken genomic DNA using the Wizard SV Genomic DNA Purification System (Promega, Madison, WI). Degenerate *ctnna3* primers were designed to conform to three conditions. (1) The primers efficiently amplify a corresponding genomic fragment of *ctnna3* from other vertebrate species. (2) The primers also amplify a corresponding genomic fragment of *ctnna1* and/or *ctnna2* from *X. tropicalis*, as well as from other vertebrate genomes, although with a lower efficiency compared to the corresponding fragment of *ctnna3*. This would serve as an internal positive control for the quality of genomic DNA and for the PCR amplification process itself. (3) The PCR product is at least 100 bp and the primer pairs belong to a single (conserved) *ctnna* exonic region.

iCODEHOP (COnsensus-DEgenerate Hybrid Oligonucleotide Primers) software [20] was used to design degenerate PCR primers from protein multiple alignments. One pair of degenerate primers that conformed to all the conditions was identified inside the last (and the longest) coding exon of the *ctnna3* gene: (a3-F) 5′-GGC TGC CAA RAA YYT NAT GAA YGC-3′ and (a3-R) 5′- GGC TTC TTT KCN GGN GCY TTC AT-3′. Both primers recognize *ctnna3* sequences, which are highly conserved in different vertebrates (Fig. S3). Moreover, the primers amplify the corresponding genomic fragments of *ctnna1* and *ctnna2* from *X. tropicalis* genomic DNA (Fig. S4). The predicted size of the PCR products obtained with these primers for all known *ctnna* genes is 144 bp. Both primers have degeneracy (number of different nucleotide sequences in the primer pool) of 64.

We used a two-step PCR protocol and a PCR machine with a gradient temperature block option. Annealing temperature was kept 45°C for all samples for the first 5 cycles and then was increased up to 54–65°C for 8 different samples (gradient block) for the last 35 cycles. The PCR reactions were run on a 2% agarose gel and an expected product about 150 bp was observed in the reactions with annealing temperatures during the second step kept from 54.1°C up to 56.3°C. These PCR products were extracted from gel, pooled, and sequenced using the a3-F and a3-R primers.

## Results

### Phylogenetic Analysis of LRRTM and α-catenin Genes in Vertebrates

**Figure 1:**
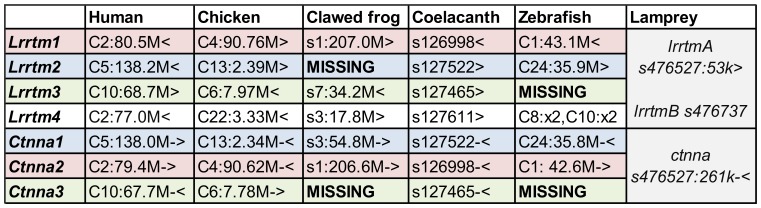
List of *lrrtm* and *ctnna* genes and their location in selected vertebrate genomes. The color shading indicates the nested/host gene pairs. Note that the clawed frog genome contains *ctnna1* and *lrrtm3* but lacks the corresponding *lrrtm2* and *ctnna3* orthologs. Both *lrrtm3* and *ctnna3* are absent in zebrafish that has four copies of *lrrtm4* (two adjacent genes in two chromosomes). Lamprey genome has two *lrrtm* genes, one of which (*lrrtmA*) is adjacent to (but not nested in) the single *ctnna* gene. The protein coding region of *lrrtm1* resides within one exon in all vertebrate species analyzed. The other *lrrtm* genes (*lrrtm2, lrrtm3,* and *lrrtm4* and lamprey *lrrtm* genes), have two (or three) protein-coding exons: the first coding exon covers the translation initiation codon and one additional coding nucleotide, while most of the open reading frame is located in the 2^nd^ coding exon. A third coding exon in *lrrtm3* and *lrrtm4* encodes for an alternative C-terminus [35].

The LRRTM family is thought to be vertebrate-specific since clear LRRTM gene homologs were originally identified in several mammalian and teleost fish genomes but not in the fruit fly or nematode genomes [1]. To study the evolution of the LRRTM family in vertebrates, we collected all annotated *lrrtm* and *ctnna* genes, and noted their corresponding genomic structures and locations, from representative model organisms (human, chicken, African clawed frog, coelacanth, zebrafish, and sea lamprey), for which whole genome sequences are available (Fig. 1). Partial *lrrtm* and *ctnna* sequences were also obtained from the elephant shark [21] and spotted gar draft genomes (Tables S1 and S2). No *lrrtm* homologs could be found from the sea squirt (*Ciona intestinalis* and *Ciona savignyi*) or from the lancelet (*Branchiostoma floridae*) genomes. The best hits from these species correspond to Slit-like and other LRR-domain containing proteins as confirmed by reciprocal BLAST search (Table S4).

**Figure 2:**
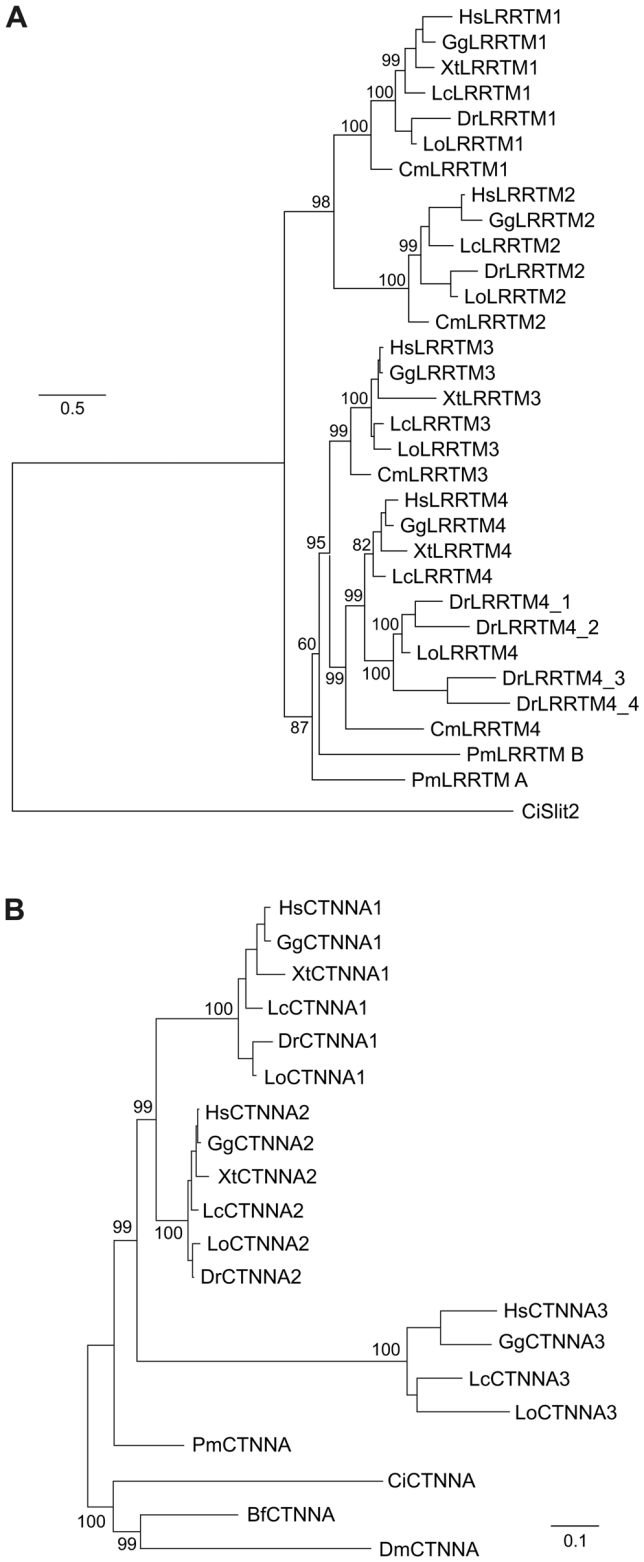
Phylogenetic trees of LRRTM and α-catenin proteins in selected vertebrates. (A) A LRRTM family tree was generated using the alignment shown in Fig. S1 and the maximum likelihood method. A tunicate LRR-domain protein CiSlit2 (one of the best BLAST hits shown in Table S4) is used to root the tree. Notice the absence of XtLRRTM2 and the divergence of XtLRRTM3 from the other vertebrate LRRTM3s. Numbers at each branch point represent bootstrap support for that branch. Bootstrap values of short terminal branches (all >90%) are omitted for clarity. The branch lengths are proportional to the expected proportion of amino acid sequence divergence ( = number of residue substitution) between groups. (B) A maximum likelihood phylogenetic tree of α-catenin proteins. Invertebrate (fruit fly, sea squirt, and lancelet) CTNNAs are included as outgroup. Note that the CTNNA3 clade has diverged more rapidly than the CTNNA1 and CTNNA2 clades during vertebrate evolution. Bf = *Branchiostoma floridae* (lancelet), Ci = *Ciona intestinalis* (sea squirt), Cm = *Callorhinchus milii* (elephant shark), Dm = Drosophilia melanogaster (fruit fly), Dr = *Danio rerio* (zebrafish), Gg = *Gallus gallus* (chicken), Hs = *Homo sapiens*, Lc = *Latimeria chalumnae* (coelacanth), Lo = *Lepisosteus oculatus* (spotted gar), Pm = *Petromyzon marinus* (sea lamprey), Xt = *Xenopus tropicalis* (African clawed frog).

To correctly identify the subtypes of the new LRRTM protein sequences in the novel species and to provide a relative time point for the divergence of the different subtypes within each family, we aligned the predicted LRRTM sequences (Fig. S1) and generated phylogenetic trees using PhyML (Fig. 2A) and MrBayes (Fig. S2). Orthologs of each LRRTM family member (LRRTM1 to LRRTM4) from different jawed vertebrate species group together forming a clade. Individual family members in the tree are located in general as expected from the known vertebrate phylogeny. Among the four LRRTMs, the highest amino acid sequence identity is seen between LRRTM3 and LRRTM4 proteins in all the analyzed jawed vertebrate species (with average pairwise sequence identities of ∼60%, Table S5). Consistent with this, the LRRTM3 and LRRTM4 clades cluster together in the phylogenetic trees. LRRTM2 proteins show higher (47–49%) pairwise sequence identity to LRRTM1 than to LRRTM3 or LRRTM4 proteins (∼40%) in all the analyzed species (Table S5). Consistent with this, the LRRTM1 and LRRTM2 clades branch together (Fig. 2A).

In a similar way, we aligned CTNNA proteins and inferred phylogenetic trees (Fig. 2B and Fig. S2). The resulting tree topology has high bootstrap support and, in agreement with a previous study [11], shows that orthologs of CTNNA1 and CTNNA2 from different jawed vertebrates form separate clades that apparently originated by duplication from a common ancestor. The CTNNA3 orthologs from different jawed vertebrates also form a distinct clade that originated before the split of the CTNNA1 and CTNNA2 proteins. However, the CTNNA3 clade has diverged clearly more from the common ancestor than CTNNA1 and CTNNA2 clades.

### Structure of *lrrtm/ctnna* Genes in Jawed Vertebrates

**Figure 3:**
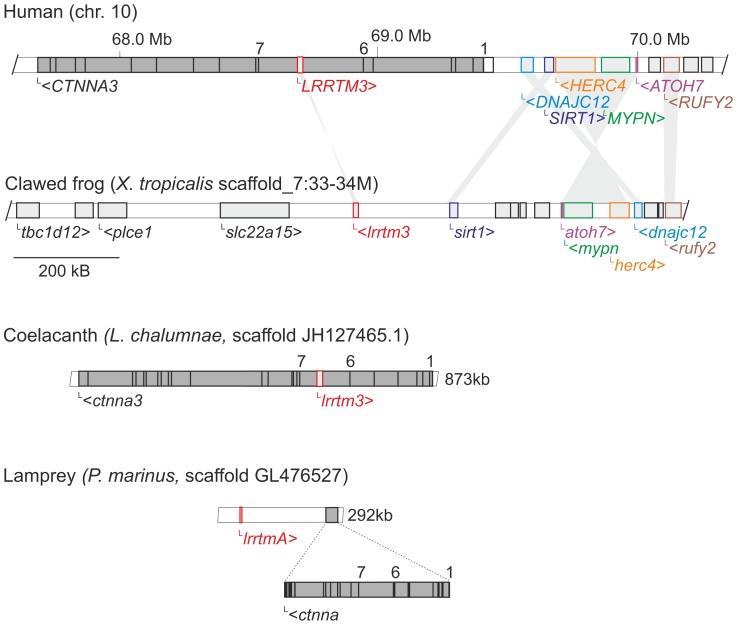
Synteny analysis of the *lrrtm3/ctnna3* locus between the human and the clawed frog genomes. Data is retrieved from Ensembl release 72, Jun 2013 and from Xentr. 7.1. Several genes in *X. tropicalis* scaffold_7 (colored) on one side of *lrrtm3* are orthologs of genes near the human *LRRTM3/CTNNA3* locus. Another cluster of genes (including *tbc1d12* and *plce1*) on the other side of *lrrtm3* is syntenic to another region of human chromosome 10 (∼96.1 Mb). However, no sequences orthogous to human *CTNNA3* exons were found in this scaffold. Genomic fragments from coelacanth, which contains a nested *lrrtm3/ctnna3* gene structure, and from lamprey, which contains adjacent *ctnna* and *lrrtmA* genes, are shown for comparison.

In all analyzed jawed vertebrate genomes (except the amphibians, see below), *lrrtm1* and *lrrtm2* are nested in a large intron between conserved coding exons 6 and 7 of α-catenin genes *ctnna2* and *ctnna1*, respectively. Similarly, *lrrtm3* gene resides in a homologous position (inside the large intron between coding exons 6 and 7) of the *ctnna3* gene in all annotated genomes of amniotes (mammals, reptiles, and birds), as well as in the lobe-finned fish coelacanth (*Latimeria chalumnae*) and the ray-finned fish spotted gar (*Lepisosteus oculatus*) genomes (Figs. 1 and 3, and Tables S1 and S2). Clear orthologs of all four *lrrtm* and three *ctnna* genes have also been found in the elephant shark genome. Nested gene structures of *lrrtm2/ctnna1* and *lrrtm3/ctnna3* are annotated, while the expected *lrrtm1/ctnna2* gene structure could not be verified because of the short size of the scaffold_422 which contains *lrrtm1* (esharkgenome.imcb.a-star.edu.sg). In contrast, the genomes of ray-finned fishes (other than the spotted gar, which diverged before the teleost fish-specific whole genome duplication [22]) lack both *lrrtm3* and *ctnna3*. Clear *lrrtm4* orthologs were found in all jawed vertebrate species analyzed. In mammals, *Lrrtm4* is located near the nested *Lrrtm1/Ctnna2* gene structure, whereas in other vertebrates, *lrrtm4* is located in a different chromosome than the *lrrtm1/ctnna2.* In contrast to other jawed vertebrates (shark, coelacanth, and tetrapods), which have a single *lrrtm4* ortholog, the analyzed genomes of ray-finned fishes (other than the spotted gar) contain four *lrrtm4* orthologs located as two closely situated genes in two chromosomes, each pair on a single chromosome being phylogenetically closer to each other (Figs. 1 and 2A, and data not shown).

Analysis of paralogous clusters of genes using the Synteny Database (syntenydb.uoregon.edu/synteny_db/) found suggestive evidence of conserved synteny between human *CTNNA1, CTNNA2,* and *CTNNA3* gene regions: a few genes (including *EGR1-4* and *REEP1-4*) that are located near the *CTNNA* genes have four paralogs in the human genome (Fig. S5). This is consistent with the idea that the three nested *lrrtm/ctnna* gene structures may have originated from two rounds of whole genome duplications in the early vertebrate lineage [23]–[25]. However, tracing back to these events is difficult. The conserved paralogous genes in human genome (*EGR1-4* and *REEP1-4*) are not immediately adjacent to the *CTNNA* genes and similar regions of conserved synteny (paralogous pairwise clusters) containing *lrrtm*/*ctnna* were not found in other vertebrate (e.g. chicken or clawed frog) genomes. *Lrrtm4* neighboring genes are not even syntenic between chicken and clawed frog and the *lrrtm4* locus is not assembled in coelacanth genome to allow analysis of synteny.

### *Lrrtm* and *ctnna* Genes in Lamprey

The genome of sea lamprey (*Petromyzon marinus)*, a jawless fish, contains two genes encoding for LRRTMs (annotated in Ensembl as LRRTM3 and LRRTM2, but named here as *lrrtmA* and *lrrtmB*, respectively), of which *lrrtmA* is situated adjacent to, but is not nested in, the single lamprey *ctnna* homolog (Figs. 1 and 3). Both *lrrtmA* and *lrrtmB* possess two protein-coding exons: the first coding exon provides only the first four nucleotides [ATGG] of the open reading frame. The structures of the predicted lamprey LRRTM (PmLRRTM_A and PmLRRTM_B) proteins with 10 LRRs, a single transmembrane domain, and a short cytoplasmic domain (with a C-terminal PDZ binding motif ECEV) are similar to that of mammalian LRRTMs [1], [2]. PmLRRTM_A and PmLRRTM_B show higher amino acid sequence identity to LRRTM3 and LRRTM4 (50–55%), than to LRRTM1 and LRRTM2 (40–45%) of other vertebrates (Table 1). In the phylogenic trees (Fig. 2A and Fig. S2) both lamprey LRRTMs branch basal to the LRRTM3-LRRTM4 divergence. Since the *lrrtmA* and *lrrtmB* reside in short scaffolds and many lamprey sequences have unresolved orthologies (possibly due to lineage-specific sequence modifications [26] and independent genome duplications [27]), it is not possible to assign origins to the two lamprey LRRTM sequences by conserved synteny analyses comparing them to other vertebrate genomes. In other words, it remains unclear whether the two sea lamprey LRRTMs originated by an independent duplication after the divergence of lampreys from the vertebrate lineage.

**Table 1:** Nested gene structures lost in clawed frog or chicken genomes.

Host gene	Nested gene	Explanation
*ASTN2*	***TRIM32***	nested absent in frog
***CACNA2D3***	***LRTM1***	both absent in frog
*CASK*	***GPR82***	nested absent in frog
***CENPP***	*ECM2*	host gene absent in frog
	*ASPN*	host gene absent in frog
	***OMD***	both absent in frog
	*OGN*	host gene absent in frog
*CTNNA1*	***LRRTM2***	nested absent in frog
***CTNNA3***	*LRRTM3*	host gene absent in frog
*FBXL13*	***LRRC17***	nested absent in frog
***FYCO1***	***CXCR6***	host absent in frog, nested absent in chicken
***IMMP2L***	*LRRN3*	host gene absent in frog
***MED12L***	*P2RY13*	host gene absent in frog
	*P2RY12*	host gene absent in frog
***PC***	***LRFN4***	both absent in chicken
*RNF123*	***AMIGO3***	nested absent in frog
***SND1***	*LRRC4*	host absent in chicken
*SYN1*	***TIMP1***	nested absent in frog
*TFB1M*	***CLDN20***	nested absent in frog

The table lists human protein-coding different strand nested gene structures that are also found in coelacanth and/or zebrafish but are absent in clawed frog (*X. tropicalis*) or chicken genomes. The genes were selected (as described in the Methods and Table S3) from a previously published list of human nested genes [12]. The missing host or nested genes are marked in bold. Nested genes that encode LRR-superfamily proteins are underlined.

The lamprey *ctnna* gene has a similar structure as other vertebrate α-catenin genes with 17 coding exons, but is much shorter (about 31 kb, compare e.g. to human *CTNNA3* that spans 1.8 Mb). However, the longest intron of lamprey *ctnna* gene (∼5.8 kb) is the one between the exons 6 and 7 that hosts *lrrtm* genes in other vertebrates. In the phylogenetic tree, the lamprey α-catenin (PmCTNNA) is basal to the jawed vertebrate branches (Fig. 2B), suggesting that it represents the common ancestor of the tree jawed vertebrate CTNNA subtypes.

### Lack of *lrrtm2* and *ctnna3* in Amphibian Genomes

Although the nested *lrrtm/ctnna* gene structure is conserved in most of the analyzed jawed vertebrate species, the genomes of the clawed frogs *Xenopus tropicalis* and *X. laevis* have two notable exceptions. First, the *X. tropicalis* genome [28] lacks an ortholog of *lrrtm2* (Fig. 1). The *X. tropicalis ctnna1*, otherwise similar in structure to α-catenin genes of other jawed vertebrates, is very compact (its length is about 16.5 kb). In particular, the intron between exons 6 and 7 of *X. tropicalis ctnna1* (that would be expected to host *lrrtm2*) is unusually short (434 bp) compared to the corresponding intron of *X. tropicalis ctnna2* (∼469 kb) hosting *lrrtm1,* or to the corresponding intron of other jawed vertebrates. Second, an ortholog for *ctnna3* is absent in the *X. tropicalis* genome, although an apparent *lrrtm3* ortholog is present (Fig. 1). Analysis of synteny confirmed that the clawed frog *lrrtm3* is indeed an ortholog of human *LRRTM3* (Fig. 3). Similar to *X. tropicalis*, the draft *X. laevis* genome (xenopus.lab.nig.ac.jp/assembly v7.1 at www.xenbase.org) lacks orthologs of *lrrtm2* and *ctnna3* but contains orthologs for all the other LRRTM and α-catenin genes. We also searched for transcripts corresponding to α-catenins in the extensive *X. tropicalis* and *X. laevis* mRNA databases (www.xenbase.org). While multiple hits are present for *ctnna1* (XB-GENEPAGE-479598) and *ctnna2* (XB-GENEPAGE-5955200), no *ctnna3* mRNAs were found by reciprocal BLAST searches. Similarly, the recently available salamander (axolotl, *Ambystoma mexicanum*) transcriptome (www.ambystoma.org) lacks orthologs of both *lrrtm2* and *ctnna3*, while clear transcripts of all the other LRRTM and α-catenin genes are present.

### Experimental Support that the *X. tropicalis* Genome Lacks *ctnna3*

**Figure 4:**
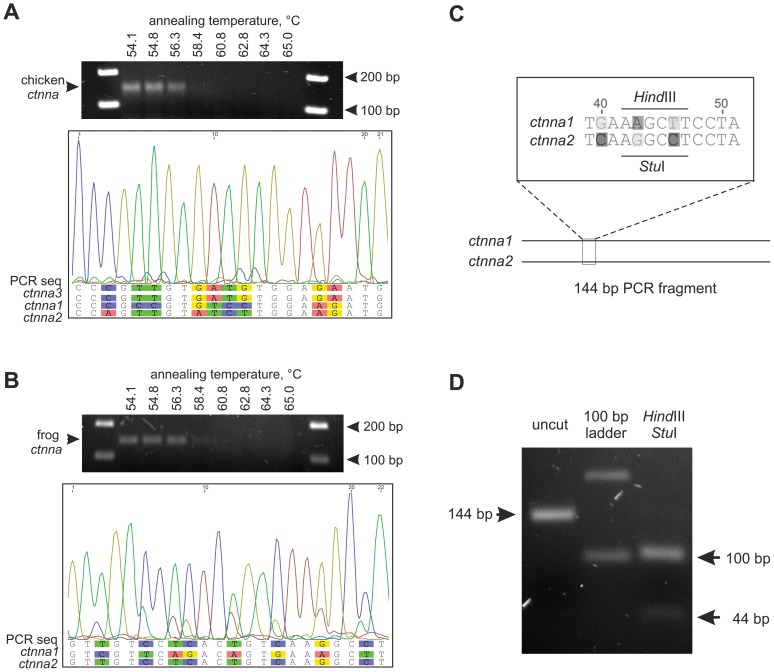
Experimental evidence that the clawed frog genome does not contain a *ctnna*3 ortholog. (A) The degenerate primers a3-F and a3-R (corresponding to the conserved last coding exon of *ctnna3* in vertebrates, see Fig. S3) were used to amplify corresponding fragments of the *ctnna* genes from the chicken genomic DNA. PCR product of the predicted size (about 150 bp) was observed using annealing temperatures from 54.1°C to 56.3°C. Sequencing of the PCR fragment (with the same primers) revealed spectra corresponding mainly to chicken ctnna3. Minor peaks corresponding to chicken ctnna1 PCR product are slightly shifted to the right. (B) The same primers were used to amplify corresponding fragments of the *ctnna* genes from the clawed frog genomic DNA. See also Fig. S4. Sequencing of the PCR fragment revealed spectra corresponding to the frog *ctnna1* and *ctnna2* genes only. Shown is a part of the sequence spectrum obtained with a3-F primer. (C) Schematic drawing of the experimental strategy. A PCR of *X. tropicalis* genomic DNA using degenerate *ctnna3* primers is expected to amplify 144 bp fragments of frog *ctnna1* and *ctnna2* that contain *Hind*III and *Stu*I restriction enzyme sites, respectively. (D) Arrow on the left points at the 144 bp PCR product obtained from the frog genomic DNA. Sequencing of this band is shown in B. Arrows on the right indicate the diagnostic *Hind*III/*Stu*I fragments of the PCR product verifying that the product is solely composed of the predicted *ctnna1* and *ctnna2* fragments.

The apparent lack of *ctnna3* in the current amphibian genomes and transcriptomes suggests loss of the *ctnna3* gene in the amphibian lineage during evolution. To obtain further support for this, we carried out polymerase chain reaction (PCR) with degenerate *ctnna3* primers (a3-F and a3-R) designed to amplify *ctnna* sequences from various species (Fig. S3). As a positive control for our strategy, we first used these degenerate primers to amplify corresponding *ctnna* fragments from the chicken genome (Fig. 4A). The primers have no mismatches with chicken *ctnna3*, but have one mismatch with a corresponding region of *ctnna1* and two mismatches with *ctnna2* (Fig. S3). Thus, the primers are expected to primarily amplify *ctnna3*, but may also amplify *ctnna1* though with a lower efficiency. Consistent with this, most of the amplified product from chicken genomic DNA corresponded to *ctnna3*, but a minor part corresponded to *ctnna1* (Fig. 4A).

The same PCR protocol was then applied to the *X. tropicalis* genomic DNA. Sequencing of the PCR fragment revealed spectra matching only to *X. tropicalis ctnna1* and *ctnna2* (Fig. 4B). Digestion with diagnostic *Hind*III and *Stu*I restriction enzymes confirmed that no other PCR products except for the frog *ctnna1* and *ctnna2* were amplified (Fig. 4C, D).

### Analysis of Selected Different-strand Nested Genes in Vertebrates

A previous study of nested genes did not report any phylogenetically old protein-coding nested gene structure that would have been lost in vertebrates [13]. To reassess whether protein-coding nested gene structures are conserved in vertebrates, we identified orthologs for the previously identified human different strand nested genes [12] in zebrafish, coelacanth, clawed frog, and chicken genomes. We included in our analysis only those gene pairs that are conserved in human and mouse, and in which a protein-coding nested gene is flanked by protein-coding exons of the host gene (see Table S3). Most of the analyzed mammalian protein-coding nested gene structures (63/91) have orthologs in the zebrafish and/or coelacanth genomes, but several of these (19/63) cannot be found in the clawed frog or chicken genomes (Table 1 and Table S3). Notably, in majority of these (12/19), the nested gene encodes for an LRR-superfamily protein.

### Evolution of Neurexin Alternative Splice Segment

The alternatively spliced segment (AS4) of neurexin protein comprises a loop structure in the binding domain, and deletion of this loop structure (by exon skipping) is required for LRRTM binding in mammals [3]–[6]. We used BLASTP search to investigate whether an exon homologous to the AS4 exon was present in *nrxn* genes of other species than the jawed vertebrates. Amino acid alignment shows that the fruit fly and sea urchin neurexin proteins lack exactly the region that is homologous to the AS4 amino acid sequence of vertebrate neurexins (Fig. S6). Moreover, in fruit fly and sea urchin *nrxn* genes, the intron between the exons that are homologous to vertebrate AS4-flanking exons is very short. In contrast, an exon homologous to the AS4-exon of mouse *Nrxn1* gene is present in the sea squirt and lamprey *nrxn* genes (Fig. S6). This suggests that the *nrxn* AS4 exon sequence appeared early in chordate evolution.

To further analyze the expression of *nrxn* isoforms lacking AS4 during evolution, we searched the available vertebrate EST databases for *nrxn* transcripts with deletion of the AS4 sequence (Table S6). As in mammals, zebrafish *nrxn* pre-mRNAs are known to undergo alternative splicing, including exon AS4 skipping [15]. We found 9 out of 23 (39%) *nrxn* ESTs that lack AS4 in human, 5 out of 27 (19%) in mouse, 1 out of 9 (11%) in clawed frog, and 1 out of 6 (17%) hits in zebrafish, confirming that this splice variant is expressed throughout the jawed vertebrate class. However, no *nrxn* EST transcripts (with or without the AS4 sequence) were found in lamprey, and therefore it remains unclear whether the alternative splicing of *nrxn* was present in jawless vertebrates. In the sea squirt (*C. intestinalis*), none of the few *nrxn* ESTs revealed the AS4 exon deletion according to our selection criteria (Table S6).

Recently it has been reported that alternative splicing of *nrxn* AS4 exon is regulated by RNA binding proteins of KHDBRS family (T-STAR and SAM68) [29], [30], which are known to recognize specific tandem repeats of UAAA/UUAA sequences in the introns adjacent to the AS4 exon [29], [31]. We therefore analyzed intronic sequences surrounding the *nrxn* AS4 exon in sea squirt (*C. intestinalis*) and sea lamprey for the presence of these repeats. In sea squirt *nrxn*, the introns surrounding the AS4-like exon are short (441 and 430 bp compared to 13620 and 1598 bp in lamprey) and contain only one UWAA (W = U/A) repeat in the upstream and three of them in downstream introns. In contrast, markedly more of the UWAA repeats can be found in both upstream and downstream proximal regions of the large introns surrounding the “AS4-exon” in two out of three lamprey *nrxn* genes (Fig. S7).

## Discussion

**Figure 5:**
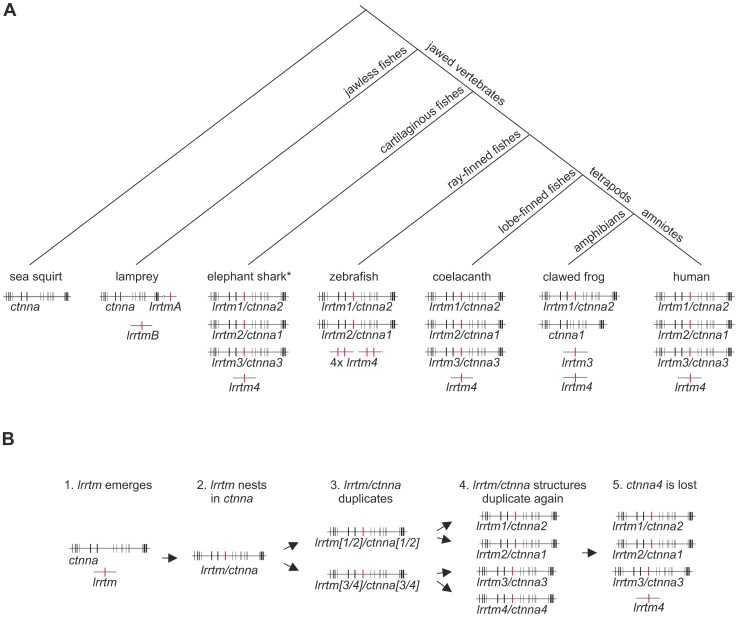
Evolution of LRRTM and α-catenin genes in vertebrates. (A) Structures of the identified *lrrtm* (red) and *ctnna* (black) genes in representative species are shown schematically below a tree of major vertebrate lineages. Intron sizes of individual *ctnna* genes are not in scale. Note that the clawed frog lacks both *lrrtm2* and *ctnna3* orthologs but has the corresponding host (*ctnna2*) and nested (*lrrtm3*) genes. Ray-finned fishes lack *lrrtm3/ctnna3* but have four copies of *lrrtm4*. (B) Hypothetical model of the nested *lrrtm/ctnna* gene structure evolution. (1) The first *lrrtm* gene emerged in the early jawless vertebrates, possibly by exon shuffling. (2) The *lrrtm* gene was translocated into a *ctnna* intron, presumably by retrotransposition (and thereby became intronless). (3–4) The nested *lrrtm/ctnna* gene structure was duplicated twice presumably as part of the two rounds of whole genome duplications that occurred at the base of vertebrates. (5) Loss of one *ctnna* host resulted in *lrrtm4* that is not nested and three nested *lrrtm/ctnna* genes present in the majority of extant jawed vertebrates.

We show here that the nested *lrrtm/ctnna* gene structure was established in early jawed vertebrates and that a conserved structure of three nested *lrrtm/ctnna* pairs is present in lobe-finned fish (and presumably also in cartilaginous fish) as in amniotes (Fig. 5A). Based on the available data, we propose a hypothetical sequence of events to explain the evolution of the nested *lrrtm/ctnna* genes (Fig. 5B).

The available genomes of invertebrates (including uro- and cephalochordates) lack clear homologs of *lrrtm*, whereas the jawless vertebrate lamprey has two copies of *lrrtm.* Thus, the first *lrrtm* presumably emerged (by exon shuffling of pre-existing genes containing extracellular LRR, transmembrane, and intracellular domains) in the early vertebrate ancestor. The α-catenin (*ctnna*) gene emerged early in metazoan evolution, presumably from a vinculin-like gene [11]. The first nested *lrrtm/ctnna* gene structure arose presumably by retrotransposition of *lrrtm* into the large intron of the nearby *ctnna* gene.

Since the nested *lrrtm1/ctnna2, lrrtm2/ctnna1,* and *lrrtm3/ctnna3* gene structures are similar in all jawed vertebrates (except for amphibians), they share a single ancestral nested *lrrtm/ctnna* gene structure that was duplicated twice presumably as a part of the two-round whole genome duplications (2R-WGD) at the origin of vertebrates [23]–[25], [27]. The two duplications resulted in four nested *lrrtm/ctnna* pairs followed by a loss of *ctnna* around *lrrtm4*. Presumably, one of the two *lrrtm/ctnna*-ancestral gene structures that emerged from the first *lrrtm/ctnna* duplication diverged to become *lrrtm[1/2]/ctnna[1/2]*-ancestral gene structure and was duplicated again, which resulted in the nested *lrrtm1/ctnna2* and *lrrtm2/ctnna1* gene structures. The other nested gene pair diverged to become *lrrtm[3/4]/ctnna[3/4]-*ancestral gene structure that was duplicated to become *lrrtm3/ctnna3* and *lrrtm4/ctnna4.* The putative *ctnna4* gene was then lost (Fig. 5B). Consistent with this model, LRRTM1 and LRRTM2 amino acid sequences are more closely related to each other than to LRRTM3 or LRRTM4, LRRTM3 shows the highest amino acid sequence identity to LRRTM4, and CTNNA1 and CTNNA2 amino acid sequences are more close to each other than to CTNNA3.

Based on the lack of *ctnna3* orthologs in most teleost fish and clawed frog genomes, previous studies have suggested that the α3-catenin would be amniote-specific [11], [32]. However, clear *ctnna3* (and *lrrtm3*) orthologs are present in the elephant shark, spotted gar, and coelacanth genomes. Thus, the *lrrtm3/ctnna3* locus was lost in the teleost fish lineage after the divergence of the spotted gar but before the teleost-specific whole genome duplication [33], [34]. In a separate event, the *ctnna3* (but not *lrrtm3*) gene was also lost in the early amphibian lineage. The assumption that the amphibians lack *ctnna3* is supported by the lack of sequences corresponding to *ctnna3* in the *X. tropicalis, X. laevis,* and *A. mexicanum* databases, our synteny analysis (Fig. 3), and PCR data (Fig. 4). Most likely *ctnna3* became non-functional by accumulating mutations and therefore unrecognizable, while *lrrtm3* remained intact. Compared to other α-catenins, the amino acid sequence of α3-catenin has diverged rapidly during vertebrate evolution (as is evident from the branch lengths in the phylogenetic tree). The expression of α3-catenin was probably initially widespread but became more restricted during subsequent vertebrate evolution. Consistent with this, mouse α3-catenin regulates the hybrid adhering junctions in the intercalated disks of the heart, which are unique to amniote vertebrates [32].

The loss of *lrrtm2* in the amphibian lineage may have occurred concomitant with (or before) the deletion of most parts of the large *ctnna1* intron. Loss of one LRRTM family member is not critical for survival in mice under laboratory conditions [2], [10], [35], [36]. We suggest that LRRTM1, which has an overlapping expression and synaptic function with LRRTM2 [1]–[6], was likely able to compensate, at least partially, for the lack of LRRTM2 in amphibians.

The precursor of *lrrtm4*, which is not nested in *ctnna* of any vertebrates, may have been initially nested in a *ctnna* that became inactive and was lost in the early vertebrates (Fig. 5B). In the teleost fish lineage, a local (probably a head-to-tail tandem) duplication followed presumably by the teleost-specific whole genome duplication [33], [34] resulted in four *lrrtm4* orthologs. As LRRTM3 and LRRTM4 proteins show highest amino acid similarity to each other, the extra copies of *lrrtm4* gene may have taken over the lack of *lrrtm3* in teleosts.

No conserved protein-coding nested gene structures were reported in a previous study to have been lost in vertebrates [13]. Therefore, it was rather unexpected that our bioinformatic analysis of 91 protein-coding different strand nested genes conserved between mouse and human [12] (see Table S3) identified 19 protein-coding nested gene structures present also in zebrafish and/or coelacanth but lost in the clawed frog or chicken genomes (Table 1). Interestingly, in 12 out of 19 cases the nested genes encoded LRR superfamily proteins. Therefore, nested LRR superfamily genes may have remained mobile during vertebrate evolution consistent with the idea that many of them have presumably derived *via* retrotransposons [13].

In mice, LRRTMs have been reported to bind specifically those neurexin isoforms that lack the alternatively spliced segment 4 (AS4) [5], [6]. *Nrxn* gene structure, including the AS4 exon, is conserved in jawed vertebrates, and *nrxn1-3* transcripts lacking this segment are expressed in zebrafish [14], [15]. Hence, the alternative splicing mechanism to skip *nrxn* AS4 exon had apparently evolved already prior to the *nrxn* gene duplications in early vertebrates. The corresponding AS4 exon is also present in the lamprey and sea squirt *nrxn* gene orthologs. However, *nrxn* gene orthologs in the fruit fly and nematode, as well as in the urochordate sea urchin, lack the sequence corresponding to the AS4 exon. This indicates that the *nrxn* AS4 exon emerged *de novo* in evolution of the chordate lineage. Recently, cerebellin (Cbln) family proteins were identified as novel neurexin ligands that may directly bind the AS4 loop [37], [38]. Interestingly, putative cerebellin gene orthologs are annotated in vertebrates, as well as in the sea squirt (*C. intestinalis*) but not in the fruit fly or nematode genomes (www.ensembl.org/Homo_sapiens/Gene/Compara_Tree?db=core;g=ENSG00000102924). We speculate that the *nrxn* AS4 exon appeared *de novo* at the same time as the gene for its new binding partner cerebellin emerged (by duplication of a related C1q/TNF-superfamily gene) in early chordates.

Recent studies have identified RNA binding proteins of KHDBRS family as key regulators of *neurexin* AS4 exon splicing in mice [29], [30]. Multiple AU-rich sequence elements in introns preceding and following AS4 exon act as the response elements including UWAA-rich regions closely downstream of AS4 that are conserved in jawed vertebrates [29], [30]. Similar UWAA-rich regions are conserved also in lamprey *neurexin* genes (Fig. S7), and the lamprey genome is known to contain KHDBRS protein orthologs [29]. In contrast, the adjacent short introns in sea squirt *neurexin* contain few UWAA motifs, and all identified *neurexin* EST transcripts from sea squirt retain the AS4 exon sequence, suggesting that the AS4 exon is not skipped in this species. Although additional studies are needed to confirm that the *neurexin* transcripts lacking AS4 are expressed in lamprey, the present evidence suggests that the LRRTMs and the mechanism of alternative splicing that enabled LRRTM binding to neurexins probably both emerged in early vertebrate evolution, before the divergence of jawed vertebrates.

Several synaptic adhesion molecules, such as neurexins, can be found in less complex metazoan organisms with a simple nervous system. However, the number of genes encoding synaptic adhesion proteins, along with other synaptic components, increased dramatically during the evolution of vertebrates [39]. LRRTMs represent an example of such adhesion proteins that are required to fine tune the formation and maintenance of synapses in the vertebrate brain, while simultaneous diversification of neurexin splice variants contributed towards the same task [7].

Recently it has been found that LRRTM4 and possibly LRRTM3 (but not LRRTM1 or LRRTM2) bind heparan sulphate proteoglycan (HSPG) glypican as a presynaptic ligand [9], [10]. We looked at the properties of the vertebrate LRRTM proteins to see if there would be any clues to how the proteins might differ, and when this function might have appeared. We noticed a correlation in total positive charge and the reported HSPG binding function in the LRRTM family: The calculated pI values are higher for the LRR-domains of mouse LRRTM3 and LRRTM4 (pI values of 9.3 and 9.4) than for mouse LRRTM1 and LRRTM2 (pI values of 6.9 and 8.1), resulting in substantial positive charge of LRRTM3 and LRRTM4 that is typical for heparin binding proteins. Similar situation is observed in case of the frog and zebrafish LRRTMs (Table S1). Interestingly, both of the LRRTMs present in lamprey are highly positively charged (pI values of 9.55 and 9.3). Thus, it seems possible that the HSPG-binding function of LRRTMs might have been present prior to their neurexin binding and then later the HSPG-binding might have been lost in the evolution of LRRTM1 and LRRTM2, which specialized to bind only neurexins. Additional experiments comparing the binding of lamprey LRRTMs to neurexins versus HSPGs are necessary to test this hypothesis.

## Conclusions

Our study provides a plausible scenario on how the LRRTMs emerged as new binding partners of neurexins. We show that *lrrtm* became nested in α-catenin gene in the early jawed vertebrates followed by gene duplications that resulted in three nested *lrrtm/ctnna* gene structures in most vertebrates. The clawed frog genome contains a clear *lrrtm3* ortholog but lacks the corresponding host (*ctnna3*) gene. We identified several other protein-coding nested gene structures that are conserved in jawed vertebrates but either the host or the nested gene is missing in the frog or chicken lineages. Interestingly, majority of these nested genes comprise LRR domains.

## Supporting Information

Figure S1 Alignment of LRRTM family protein sequences from selected vertebrates.
(PDF)

Figure S2 Bayesian phylogenetic trees of LRRTM and α-catenin proteins.
(PDF)

Figure S3 Alignment shows that the degenerate *ctnna3* forward (a3-F) and reverse (a3-R) primers have no mismatches with chicken *ctnna3* genomic sequence.
(PDF)

Figure S4 Sequence alignment of the predicted 144 bp *ctnna1* and *ctnna2* PCR fragments.
(PDF)

Figure S5 Paralogous clusters containing α-catenin genes in human genome.
(PDF)

Figure S6 Presence of *neurexin* AS4 exon in selected animal species.
(PDF)

Figure S7 Comparison of UWAA motifs (arrows) within 200 nucleotides (A) upstream and (B) downstream of exon AS4 in human, lamprey and sea squirt *neurexin* genes.
(PDF)

Table S1 Accession numbers, curated LRRTM amino acid sequences and pI values.
(PDF)

Table S2 Accession numbers for the α-catenin sequences used.
(PDF)

Table S3 Conservation of selected human different strand nested gene structures in vertebrate genomes.
(PDF)

Table S4 BLAST analysis of tunicate and lancelet genomes using lamprey LRRTM sequences as query and reciprocal BLAST analysis against vertebrate genomes.
(PDF)

Table S5 Amino acid sequence identity (%) between vertebrate LRRTMs.
(PDF)

Table S6 Alternative splicing of *neurexin* AS4 exon in selected species.
(PDF)
