# Supplementary material for: Origin and Loss of Nested LRRTM/α-Catenin Genes during Vertebrate Evolution
Source: PLoS One. 2014 Feb 24;9(2):e89910. doi: 10.1371/journal.pone.0089910 (PMC3933685; doi:10.1371/journal.pone.0089910)
Supplement: Figure S1 — Alignment of LRRTM family protein sequences from selected vertebrates. (PDF) [file pone.0089910.s001.pdf]

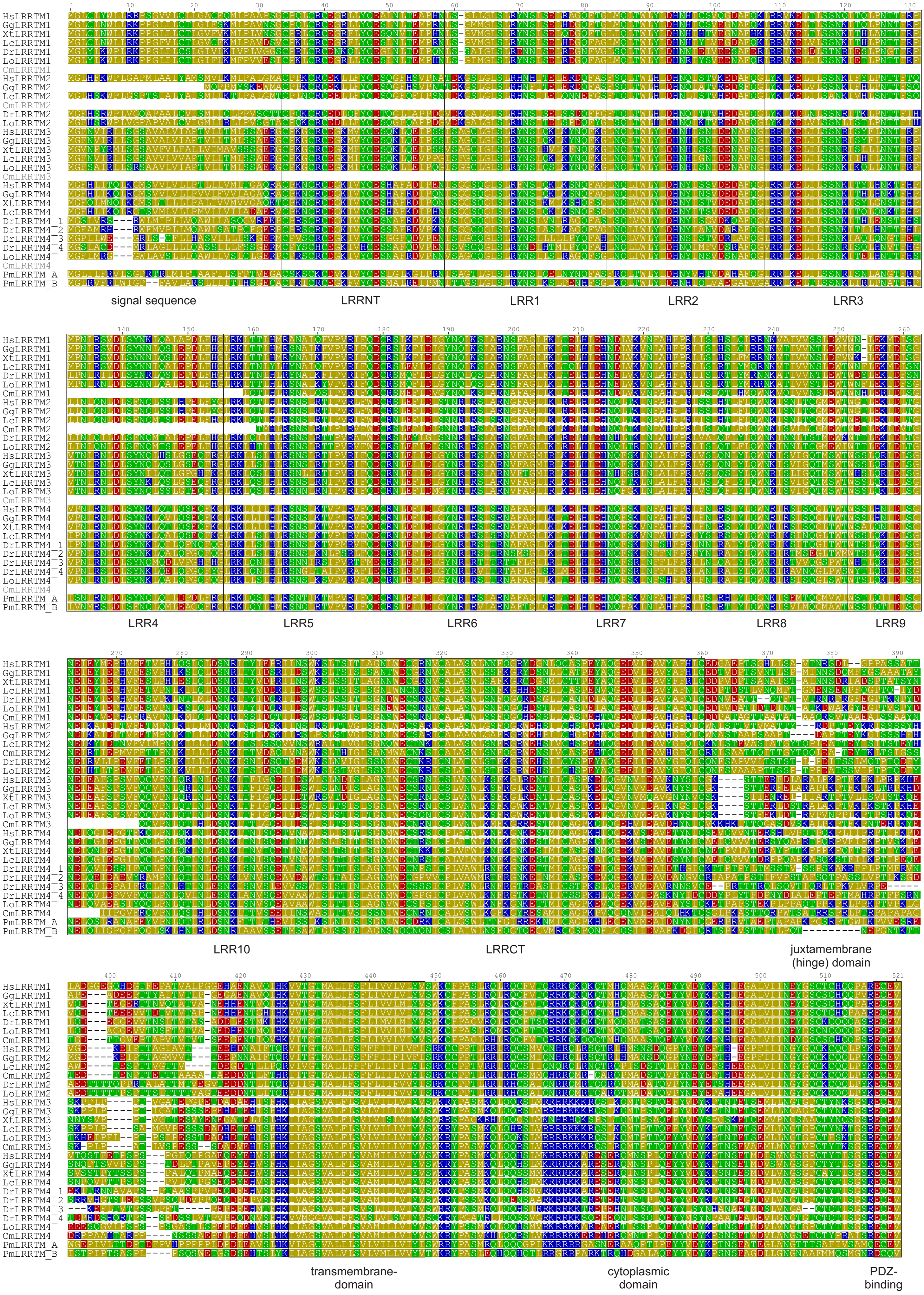

Fig. S1. Alignment of LRRTM family protein sequences from selected vertebrates. Amino acids are colored according to polarity and predicted domains are marked. All LRR motifs fit to the "typical" LRR-class consensus sequence [Kobe B and Kajava AV (2001) Curr. Opin. Struct. Biol. 11: 725-732]. Cm = *Callorhinchus milii* (elephant shark), Dr = *Danio rerio* (zebrafish), Gg = *Gallus gallus* (chicken), Hs = *Homo sapiens*, Lc = *Latimeria chalumnae* (coelacanth), Lo = *Lepisosteus oculatus* (spotted gar), Pm = *Petromyzon marinus* (sea lamprey), Xt = *Xenopus tropicalis* (African clawed frog).
