# Supplementary material for: Origin and Loss of Nested LRRTM/α-Catenin Genes during Vertebrate Evolution
Source: PLoS One. 2014 Feb 24;9(2):e89910. doi: 10.1371/journal.pone.0089910 (PMC3933685; doi:10.1371/journal.pone.0089910)
Supplement: Figure S2 — Bayesian phylogenetic trees of LRRTM and α-catenin proteins. (PDF) [file pone.0089910.s002.pdf]

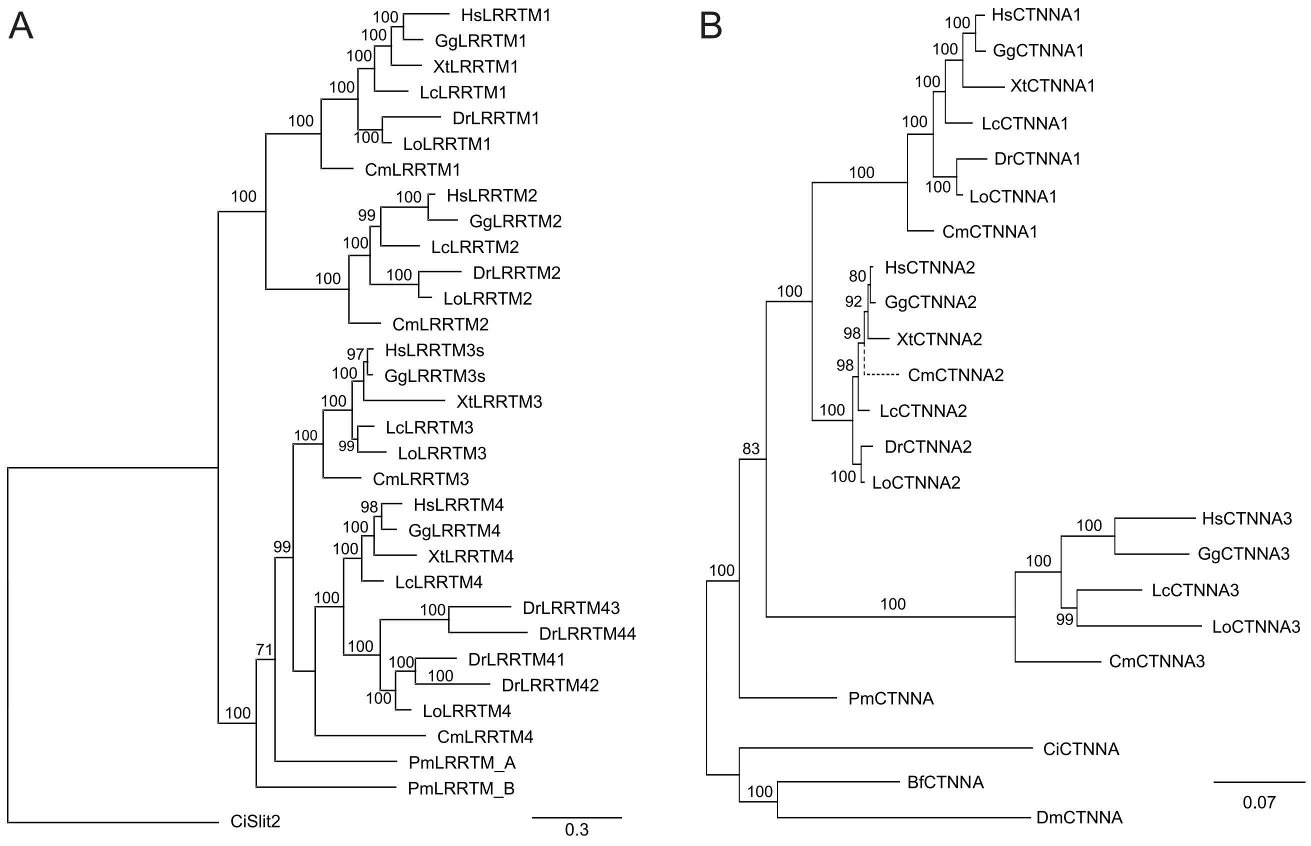

Figure S2. Phylogenetic trees of vertebrate LRRTM and α-catenin proteins inferred by bayesian method. We used MrBayes 3.2 (Ronquist F, Teslenko M, van der Mark P, Ayres DL, Darling A, Höhna S, Larget B, Liu L, Suchard MA, Huelsenbeck JP. Syst Biol. 2012; 61(3):539-42. doi: 10.1093/sysbio/sys029) without metropolis coupling with default parameters and sampling with mixed amino acid matrices. At 200 000 generations, the search had converged based on statistics. (A) A LRRTM tree was inferred using the alignment shown in Fig. S1. CiSlit2 was used to root the tree. Numbers at each branch point represent probability support for that branch. (B) Phylogenetic tree of vertebrate α-catenins using invertebrate α-catenins as outgroup. The amino-acid sequences used are shown in Table S2. The CmCTNNA2 branch (stippled) is apparently misplaced because of incomplete sequence data. Bf = *Branchiostoma floridae* (lancelet), Ci = *Ciona intestinalis* (sea squirt), Cm = *Callorhinchus milii* (elephant shark), Dm = Drosophilia melanogaster (fruit fly), Dr = *Danio rerio* (zebrafish), Gg = *Gallus gallus* (chicken), Hs = *Homo sapiens*, Lc = *Latimeria chalumnae* (coelacanth), Lo = *Lepisosteus oculatus* (spotted gar), Pm = *Petromyzon marinus* (sea lamprey), Xt = *Xenopus tropicalis* (African clawed frog).
