# Supplementary material for: Origin and Loss of Nested LRRTM/α-Catenin Genes during Vertebrate Evolution
Source: PLoS One. 2014 Feb 24;9(2):e89910. doi: 10.1371/journal.pone.0089910 (PMC3933685; doi:10.1371/journal.pone.0089910)
Supplement: Figure S3 — Alignment shows that the degenerate ctnna3 forward (a3-F) and reverse (a3-R) primers have no mismatches with chicken ctnna3 genomic sequence. (PDF) [file pone.0089910.s003.pdf]

**a3-F**		**5′-GGCTGCCAARAAYYTNATGAAYGC-3′**
ctnna	lamprey	GGCAGCCAAGAACCTGATGAACGC
ctnna3	coelaca	GGCTGCCAAGAACTTGATGAATGC
ctnna3	chicken	GGCTGCCAAAAATTTAATGAACGC
Ctnna3	mouse	GGCAGCCAAGAATTTAATGAATGC
ctnna1	xenopus	AGCTGCGAAAAATCTGATGAATGC
ctnna2	xenopus	GGCAGCTAAGAATTTGATGAATGC
ctnna1	chicken	GGCGGCCAAGAACCTGATGAACGC
ctnna2	chicken	GGCAGCTAAAAATCTGATGAACGC

**a3-R**		**5′-GGCTTCTTTKCNGGNGCYTTCAT-3′**
ctnna	lamprey	GGCTTCTTCTCGGGTGCTTTCAT
ctnna3	coelaca	GGCTTTTTTTCTGGAGCCTTCAT
ctnna3	chicken	GGCTTCTTTTCTGGAGCTTTCAT
Ctnna3	mouse	GGCTTCTTAGCCGGAGCCTTCAT
ctnna1	frog	GGTTTCTTTTCTGGAGCCTTCAT
ctnna2	frog	GGTTTCTTTTCAGGTGCTTTCAT
ctnna1	chicken	GGCTTCTTCTCCGGAGCCTTCAT
ctnna2	chicken	GGTTTCTTCTCAGGTGCCTTCAT

Figure S3. Alignment shows that the degenerate *ctnna3* forward (a3-F) and reverse (a3-R) primers have no mismatches with chicken *ctnna3* genomic sequence. Both primers have one mismatch (yellow) with the corresponding region of chicken *ctnna1* and two mismatches with chicken *ctnna2*, suggesting that the primers preferentially amplify *ctnna3,* and *ctnna1* with a higher efficiency than *ctnna2* from chicken genomic DNA.
