# Supplementary material for: Origin and Loss of Nested LRRTM/α-Catenin Genes during Vertebrate Evolution
Source: PLoS One. 2014 Feb 24;9(2):e89910. doi: 10.1371/journal.pone.0089910 (PMC3933685; doi:10.1371/journal.pone.0089910)
Supplement: Figure S4 — Sequence alignment of the predicted 144 bp ctnna1 and ctnna2 PCR fragments. (PDF) [file pone.0089910.s004.pdf]

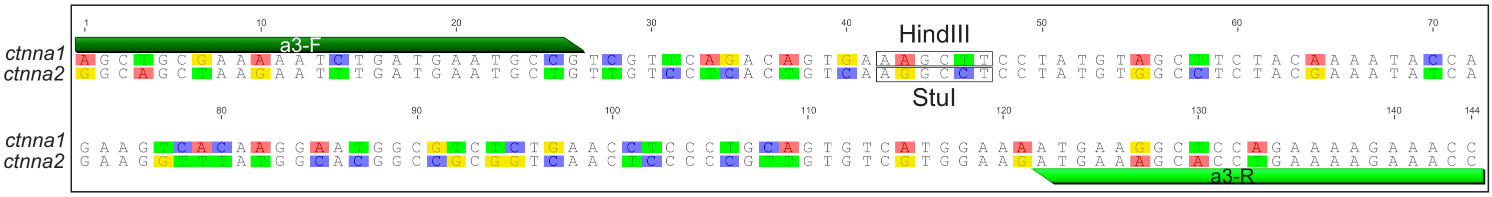


*Xenopus tropicalis*

**Figure S4.** Sequence alignment of the predicted 144 bp *ctnna1* and *ctnna2* PCR fragments (sense strands only) obtained with primers a3-F and a3-R from the frog (*X. tropicalis*) genomic DNA. Positions of the unique *Hind*III and *Stu*I restriction sites are also marked.
