# Supplementary material for: Origin and Loss of Nested LRRTM/α-Catenin Genes during Vertebrate Evolution
Source: PLoS One. 2014 Feb 24;9(2):e89910. doi: 10.1371/journal.pone.0089910 (PMC3933685; doi:10.1371/journal.pone.0089910)
Supplement: Figure S5 — Paralogous clusters containing α-catenin genes in human genome. (PDF) [file pone.0089910.s005.pdf]

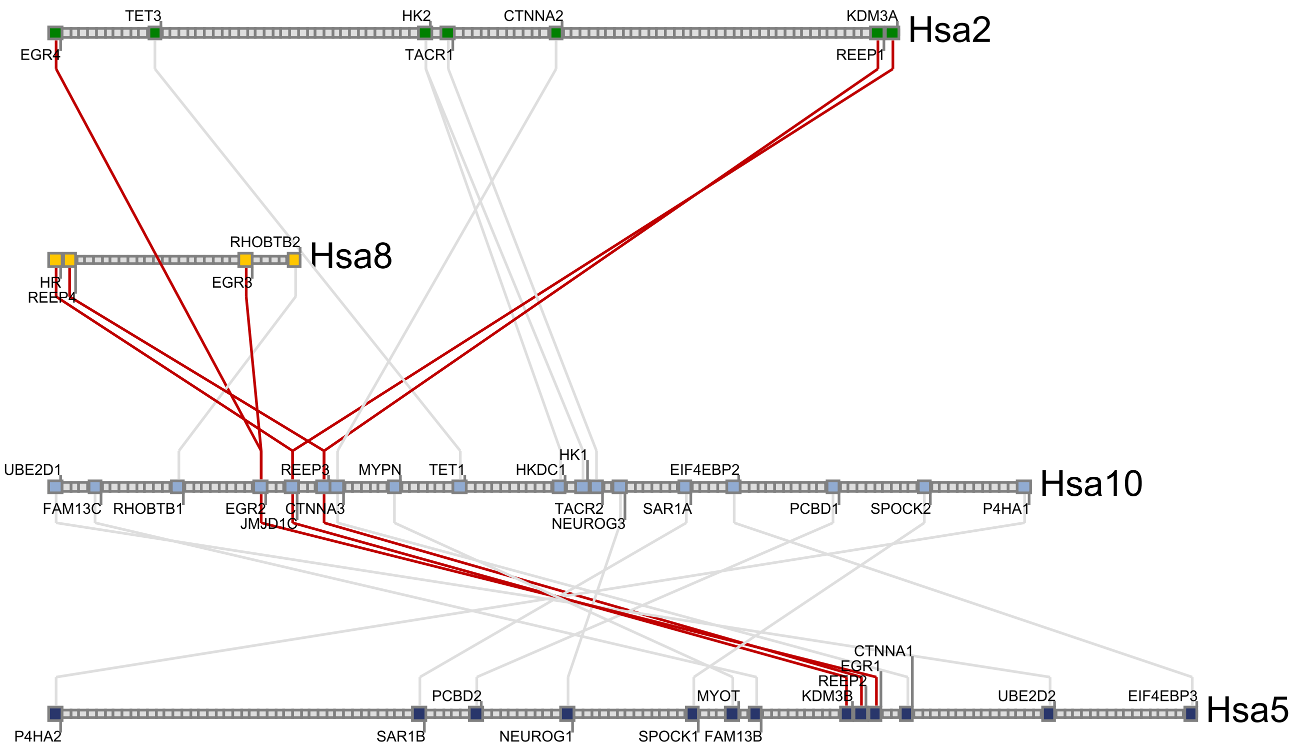

Figure S5. Paralogous clusters containing *CTNNA* genes in the human genome. An example of automatic output from the Synteny Database using *EGR2* as a query, sliding window size = 50 and *Ciona intestinalis* as outgroup. The result shows that a few genes (including *EGR1-4* and *REEP1-4*) that are near *CTNNA* genes have four paralogs in the human genome consistent with the idea that the three *CTNNA*s may have originated by two rounds of whole genome duplications.
