# Supplementary material for: Origin and Loss of Nested LRRTM/α-Catenin Genes during Vertebrate Evolution
Source: PLoS One. 2014 Feb 24;9(2):e89910. doi: 10.1371/journal.pone.0089910 (PMC3933685; doi:10.1371/journal.pone.0089910)
Supplement: Figure S6 — Presence of neurexin AS4 exon in selected animal species. (PDF) [file pone.0089910.s006.pdf]

Figure S6.

Shown are best hits (accession numbers) from a BLASTP search against (PEP_ALL) of the selected animals using the following 160 residue fragment of mouse NRXN1 (Refseq NP_064648.3, residues 1184-1343) as a query:

```
QGKIGVKFNVGTDDIAIEESNAIINDGKYHVVRFTRSGGNATLQVDSWPVIERYPAGNNDNERLAIARQRIPYRLGRVVD
EWLLDKGRQLTIFNSQATIIIGGKEQGQPFQGQLSGLYYNGLKVLNMAAENDANIAIVGNVRLVGEVPSSMTTESTATAM
```

(Amino acid residues encoded by the AS4 exon are highlighted in yellow and residues encoded by the AS4-flanking exons are in blue and green)

Drosophila melanogaster (Dm-Nrxn FBtr0301485)

```
Query  1     QGKIGVKFNVGTDDIAIEESNAIINDGKYHVVRFTRSGGNATLQVDSWPVIERYPAGNND  60
             +G I + +N+G+ D+ + E    +ND  YHVVRF R GGNATLQ+D + V    P
Sbjct  1257  EGNIFMVYNIGSVDLPLGEIGTKVNDNAYHVVRFQRKGGNATLQLDDYNVQALTPQ----  1306

Query  61    NERLAIARQRIPYRLGRVVDEWLLDKGRQLTIFNSQATIIIGGKEQ-------GQPFQGQ  115
                                           T+FN+ + + +GGK          +PF G
Sbjct  1307  --------------------------SHHSTVFNTMSNVQVGGKFSRNGRNRIERPFAGV  1346
```

Note that the AS4-flanking exons of mouse *Nrxn1* are homologous to exons in Dm-*nrxn* gene that are separated by a short (76 bp) intron!

Strongylocentrotus purpuratus (Sp-Neurx SPU_024416)

```
Query  3     GKIGVKFNVGTDDIAIEESNAIINDGKYHVVRFTRSGGNATLQVDSWPVIERYPAGNNDN  62
             G +   +N G+ D  I +    INDG YHV+ F+R+G NA+LQ+D +  I + P
Sbjct  1144  GYLWTMYNFGSADHMISDDTNRINDGSYHVIHFSRTGANASLQIDQYARIHKTP------  1197

Query  63    ERLAIARQRIPYRLGRVVDEWLLDKGRQLTIFNSQATIIIGGKEQ--------------G  108
                                     KG+Q T F+ QA I +GG+ +
Sbjct  1198  ------------------------KGKQSTFFDDQAIINLGGRGEMSDSKRKRRRRAPAS  1233
```

Sp-*neurexin* gene has exons that are homologous to the AS4 flanking exons of mouse *Nrxn1.* The intron in Sp-*Neurx* between the exons is ~500 bp. Genescan analysis of this intron failed to find any putative exons (= ORFs between symmetric splice sites in the correct reading frame).

Ciona intestinalis (nrxn ENSCINT00000010501)

```
Query:    3 GKIGVKFNVGTDDIAIEESNAIINDGKYHVVRFTRSGGNATLQVDSWPVIERYPA---GN 59
            G++ ++FN+G  D+ ++E + + +DG+YH VR  R   NATL+VD+WP  ER      G+
Sbjct: 1181 GRVTLRFNIGNSDVKLQEHSPV-SDGQYHTVRVRRKWMNATLRVDNWP--ERTATTTGGS 1237

Query:   60 NDNERLAIARQRIPYRLGRVVDEWLLDKGRQLTIFNSQATIIIGGK-EQGQP-FQGQLSG 117
             D  R A+A+  IP  +   V+ WL  K    T+FNSQ+ I IGG  +Q  P F+GQ+ G
Sbjct: 1238 RD--RFALAKPSIPTSVLSTVETWLRSKEYSNTVFNSQSVIQIGGLLDQSSPYFHGQMMG 1295
```

Petromyzon marinus (nrxn1a ENSPMAP00000006258)

```
Query:   3 GKIGVKFNVGTDDIAIEESNAIINDGKYHVVRFTRSGGNATLQVDSWPVIERYPAGNNDN 62
           GKIGVKFNVGTDDIA+EE+N+++NDGKYHVVRF+RSGGNATLQ+D+ PV ER+P+GN DN
Sbjct:  64 GKIGVKFNVGTDDIAVEEANSLVNDGKYHVVRFSRSGGNATLQLDNLPVNERFPSGNIDN 123

Query:  63 ERLAIARQRIPYRLGRVVDEWLLDKGRQLTIFNSQATIIIGGKEQGQPFQGQLSGLYYNG 122
           ERLA+ARQRIPYRLGRVVD+WLLDKGRQLTIFNSQATI IGGK+  +PFQGQLSG+YYNG
Sbjct: 124 ERLALARQRIPYRLGRVVDDWLLDKGRQLTIFNSQATIKIGGKDNNKPFQGQLSGIYYNG 183
```
