# Supplementary material for: Origin and Loss of Nested LRRTM/α-Catenin Genes during Vertebrate Evolution
Source: PLoS One. 2014 Feb 24;9(2):e89910. doi: 10.1371/journal.pone.0089910 (PMC3933685; doi:10.1371/journal.pone.0089910)
Supplement: Figure S7 — Comparison of UWAA motifs (arrows) within 200 nucleotides (A) upstream and (B) downstream of exon AS4 in human, lamprey and sea squirt neurexin genes. (PDF) [file pone.0089910.s007.pdf]

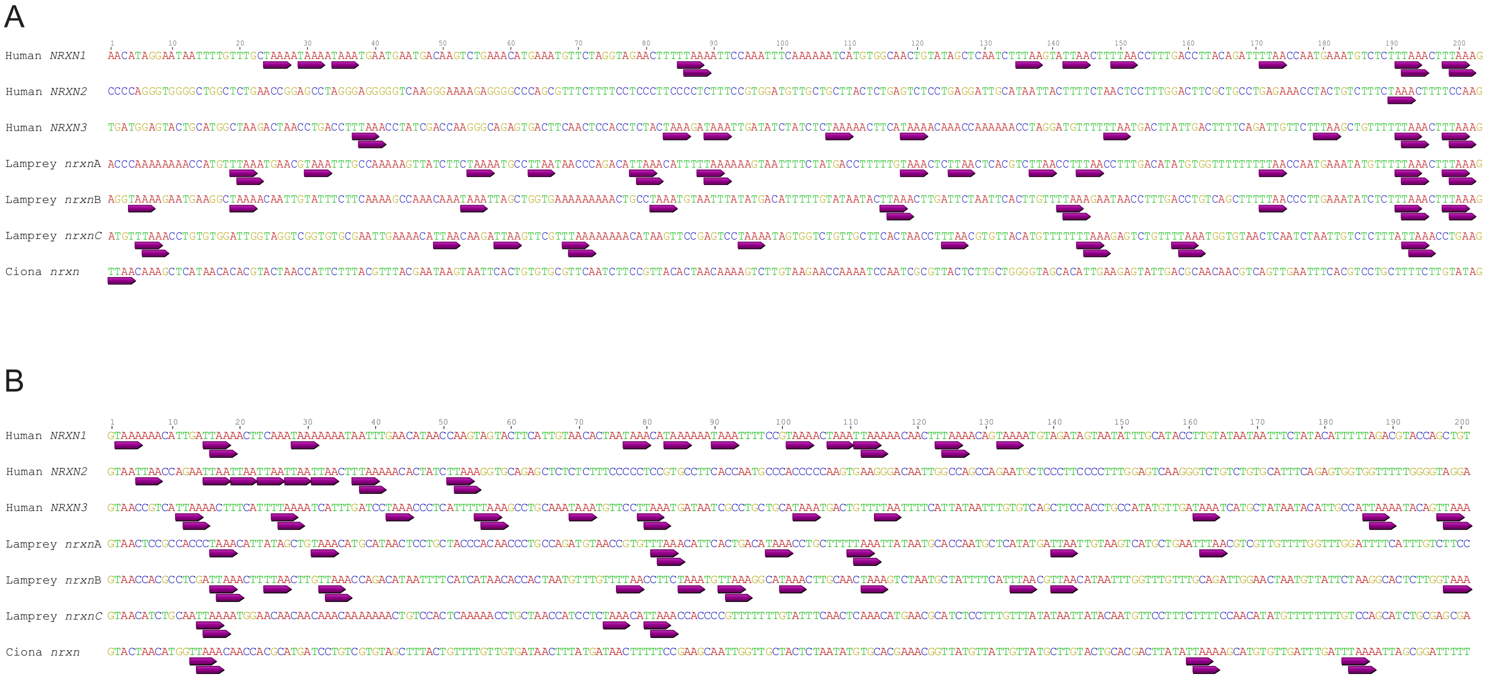

Figure S7. Comparison of UWAA motifs (arrows) within 200 nucleotides (A) upstream and (B) downstream of exon AS4 in human, sea lamprey and sea squirt (*Ciona intestinalis*) *neurexin* genes. The number and pattern of the motifs in lamprey *nrxn* genes is more similar to that in human *NRXN1* and *NRXN3* as compared to that in human *NRXN2*. In contrast, the corresponding upstream and downstream intronic regions in sea squirt *nrxn* contain few UWAA motifs.
Upstream sequences: *C. intestinalis nrxn* (Chr3:963351-963149); *H. sapiens*: *NRXN1* (Chr2:50282183-50282385), *NRXN2* (Chr11: 64394227-64394025), *NRXN3* (Chr14:80158313-80158515); *P. marinus*: *nrxnA* (GL476386:445307-445509), *nrxnB* (GL476911:123470-123672), *nrxnC* (GL477834: 72483-72281).
Downstream sequences: *C. intestinalis nrxn* (Chr3:963061-962861); *H. sapiens*: *NRXN1* (Chr2:50281892-50282092), *NRXN2* (Chr11:64393934-64393734), *NRXN3* (Chr14:80158606-80158806); *P. marinus*: *nrxnA* (GL476386:445600-445800), *nrxnB* (GL476911:123763-123963), *nrxnC* (GL477834:72184-71984).
