# Supplementary material for: Origin and Loss of Nested LRRTM/α-Catenin Genes during Vertebrate Evolution
Source: PLoS One. 2014 Feb 24;9(2):e89910. doi: 10.1371/journal.pone.0089910 (PMC3933685; doi:10.1371/journal.pone.0089910)
Supplement: Table S1 — Accession numbers, curated LRRTM amino acid sequences and pI values. (PDF) [file pone.0089910.s008.pdf]

Table S1. LRRTM protein accession numbers, pI values of LRR domains, and manually curated amino-acid sequences used for the alignment.

Protein	pI value*	Refseq	Ensembl	Elephant shark genome
HsLRRTM1	7.5	NP_849161.2		
GgLRRTM1	7.5	XP_004936330.1		
XtLRRTM1	7.8	XP_002935525.1		
LcLRRTM1	6.5	XP_006014691.1		
DrLRRTM1	5.4	XP_005160139.1		
LoLRRTM1	6.2		ENSLACP00000013386_1	
CmLRRTM1	(5.9)			AAVX01003276.1
HsLRRTM2	7.8	NP_056379.1		
GgLRRTM2	7.9	XP_004944826.1		
LcLRRTM2	7.2	XP_006003760.1		
DrLRRTM2	7.3	XP_003201382.1		
LoLRRTM2	6.8		ENSLOCP00000022413	
CmLRRTM2	(8.1)			AAVX01197253.1
HsLRRTM3	9.6	NP_821079.3		
GgLRRTM3	9.6		ENSGALP00000033454	
XtLRRTM3	9.8	XP_002936907.2		
LcLRRTM3	9.7	XP_006003251.1	ENSLACP00000012957	
LoLRRTM3	9.5		ENSLOCP00000014278	
CmLRRTM3	(7.9)			AAVX01197253.1
HsLRRTM4	9.5	NP_079269.4		
GgLRRTM4	9.6	XP_417690.3		
XtLRRTM4	9.6	XP_002932606.1		
LcLRRTM4	9.3	XP_006004503.1		
DrLRRTM4-1	9.7	XP_005172519.1	ENSDARP00000077281	
DrLRRTM4-2	9.2	XP_692159.4	ENSDARP00000097998	
DrLRRTM4-3	8.9	XP_697133.6	ENSDARP00000100212	
DrLRRTM4-4	8.2	XP_001340869.4	ENSDARP00000101739	
LoLRRTM4	8.9		ENSLOCP00000019846	
CmLRRTM4	(8.2)			AAVX01198974.1
PmLRRTM_A	9.2		ENSPMAP00000011013	
PmLRRTM_B	9.6		ENSPMAP00000007010	

*The pI values are for the extracellular parts without the signal sequence and hinge domains. (Values for the partial shark sequences are not comparable).

```
>HsLRRTM1
MDFLLLGLCLYWLLRRPSGVVLCLLGACFQMLPAAPSGCPQLCRCEGRLLYCEALNLTEAPHNLSGLLGLSLRYNSLSELRAGQFTGLMQL
TWLYLDHNHICSVQGDAFQKLRRVKELTLSSNQITQLPNTTFRPMPNLRSVDLSYNKLQALAPDLFHGLRKLTTLHMRANAIQFVPVRIFQ
DCRSLKFLDIGYNQLKSLARNSFAGLFKLTELHLEHNDLVKVNFAHFPRLISLHSLCLRRNKVAIVVSSLDWVWNLEKMDLSGNEIEYMEP
HVFETVPHLQSLQLDSNRLTYIEPRILNSWKSLTSITLAGNLWDCGRNVCALASWLNNFQGRYDGNLQCASPEYAQGEDVLDAVYAFHLCE
DGAEPTSGHLLSAVTNRSDLGPPASSATTLADGGEGQHDGTFEPATVALPGGEHAENAVQIHKVVTGTMALIFSFLIVVLVLYVSWKCFPA
SLRQLRQCFVTQRRKQKQKQTMHQMAAMSAQEYYVDYKPNHIEGALVIINEYGSCTCHQQPARECEV

>GgLRRTM1
MDFLLIGLCLNWLLRKPPGLILCTLGIFSKMLPAVNSGCPQLCRCEGRLLYCESLNLTEMPRNLSGMMGLSLRYNSLSELHDGQFTGLMQL
TWLYLDHNHICSVEGNAFQKLRRVKELTLSSNKITQLPNNTFRPMPNLRSVDLSYNNLQSLEPDLFHGLRKLTTLHMRSNAIKFVPVRIFQ
DCRSLKFLDIGYNQLKSLARNSFAGLFKLTELHLEHNDLVKVNLAHFPRLISLHSLCLRRNKVTIVVNTLDWIWQLEKLDLSGNEIEYIEP
HVFESVPHLKSLQLDSNRLTYIDSRILDSWKSLTSISLSANTWDCGRNVCALASWLSSFKGRYDSNLLCATPEYAQGEDVLDAVYAFHLCE
DTVDPTSVNTLSPMINNSDQMFGYGSAAATAYAPEADEEPTTYAITVTLPGEGAENAVQIHKVVTGTMALIFSFLIVVLVLYVSWKCFPAS
IRQLRQCFVTQRRKQKQKQTMHQMAAMSAQEYYVDYKPNHIEGALVIINEYGSCSCHQQPVRECEV
```

```
>XtLRRTM1
MDFLLIGLCLNWLLRKPPGLILCTLGVFVKMLPLVNSGCPRLCRCEGRFLYCESQNVTEIPHNLSGVMGLSLRYNSLSELQDGQFTGLIQL
TWLYLDHNHIHTVEGNAFHKLRRVKELTLSSNKITHLANTTFRPMPNLRSVDLSNNNLQSLEADLFHGLRKLTTLHMRYNAIKFVPVRIFQ
DCRSLKFLDLGYNQLKSLARNSFAGLFKLTELHLEHNDLVKVNLAHFPRLLSLHSLFMRRNKVTIVVNSLDFVWKLEKMDLSGNEIEYIEP
HVFESLPHLESLQLDSNRLTYVDPRILNSWKSLSSITLAGNNWDCGRNVCALASWLSAFKGRCDGNMLCTTPEYAQGEDVLDAVYFFRLCD
DPVDPTSANAISTALNNSDRIAIDSPTATSYNVQDTEGERTTNVQTATVANEHHENTVQIHKVVTGTMALIFSFLIVVLMLYVSWKCFPAS
LRQLRQCFVTQRRKQKQKQTMHQMASMSAQEYYVDYKPNHIEGALVIINEYGSCSCHQQPARECEV

>LcLRRTM1
MDFLLLGLCLNWLLRKPPGFVLCTLGVCFKMLPAVDSVCPKLCRCEGRFLYCESLNLTEMPRNLSGVMGLSLRYNSLSELHDGQFAGLMQL
TWLYLDHNHIYLVEGDAFQKLRRVKELVLSSNKITHLPNTTFRPMPNLRSVDLSYNNIQALEPDLFHGLRKLTNLYLHYNAIQFVPVRIFQ
DCRSLEVLDLGYNQLKSLARNSFAGLFKLTQLHLEHNDLVKVNLAHFPRLLSLRTLYMQRNKVTIVVNSLDWTWNFLEKMDLSNNEIEYIE
PHVFETVPNLKILQLDSNRLTYVDDRILDSWSSLTSISLSGNIWNCNRNVCALASWLSNFKGHHDSSLLCASPENVQGEDILDAVYAFNLC
EDPTDSTLVNQMPTGMENSENPFDQGPTQIYDVQDTEEEVVTDTVTVTAPNEHHENTGHIHKVVTGTMALIFSFLIVVLVLYVSWKCFPAS
LRQLRQCFVTQRRKQKQKQTMHQMAAMSAQEYYVDYKPNHIEGALVIINEYGSCTCHQPAARECEV

>DrLRRTM1
MDFLLIGLYLKWPLRKPPGLILCSLGIVLKIVPLVGGSCPRLCRCDNKQLYCEGLNLTDIPQNLSSAIGLSLRENNISELREGNFVGLSQL
TWLYLDHNNIEIVEESAFERLRRIKELDLSTNRIESLPNGTFRPLPNLRILDLSYNRLQSLEPDLFHGLRKLTNLHLRYNALKFIPVRIFQ
DCRSMQFLDLGYNQLQSLARNSFAGLFKLTELHLEHNELVKVNLAHFPRLISLRTLYMRNNKATIVVNTLEWTWDYLEKIDFSNNEIEYIE
PHVFESVPKLNTLMLDSNKLTYIDQRILDSWTSLSSITLSGNDWECSRNVCALASWLSNFQGQRDSGLLCANPDIAQGEDILDAVYAFQLC
EDNVEATTQTFTVTRNRPRGFIYEGPTKNPYDLQDMEGGEVVTNSLTVTASADDLESTMKIHKVVTGTMALIFSFLIMVLMLYVSWKCFPA
GVRQLRQCFTSQRRKQKQKQTMQQMATTSASEYYVDYKPNHIDGGLVIINEYGSCKCQQQASRECEV

>LoLRRTM1
MDFLLIGLYLKWLLRKPPGLILCTLGIFLKMFPVVESICPKLCRCDGRLLYCESLNLTEMPHNLSGIMGLSLRYNSLSELRDGQFAGLMQL
TWLYLDHNRIYSVEENAFQRLRRVKELILSSNKIETLPNTTFRPMPNLRNLDLSYNNLQALEPDLFHGLRKLTTLHLRSNALKYVPVRIFQ
DCRSMQFLDLGYNQLQSLARNSFAGLLKLTELHLEHNELVKVNLAHFPRLISLRTLYMRRNKATIVVSTLEWTWNFLEKIDFSGNEIEYIE
PHVFESVPNLKSLQLDSNRLTYIDQRILDSWTSLTSITLSGNEWECSRNVCALASWLSNFQGQHDSTLLCASPEIAQGEDVLDAIYAFQLC
EDPVDATIDTDINTATKDWAKGYSKEGPTVSPYDLQDAGGEVVTNSFTVTVANEDHENTMQIHKVVTGTMALIFSFLIVVLMLYVSWKCFP
ASLRQMRQCFTTQRRKQKQKQTMQQMATMSAQEYYVDYKPNHIEGALVIINEYGSCNCQQQPSRECEV

>CmLRRTM1
LQTLHLRSNALQSIPVRIFQDCRSMEFLDVGYNQLKSLARNAFAGLLKLTELHLEHNDLVKVNFAHFPRLLSLQTLHMQWNKVQIVVNSLE
WTWNHLERLDLSGNEIEYVEPHAFRAVPNLKMLQLDSNRLSSIDQTILDSWSSLTSISLSGNSWECSRNICALATWLSSFQGQHESSLLCA
SPEHTQGEDVLDAVHGFHICDDLDTSTPATAGTTAATATAAQRSVTAAGEALSSAAYNTGDTTGVEPTAPVTVTVTSEEGSPEPRENTIQV
HKVITGTMALLFSFLIVVLVLYVSWKCFPAGLRHLRQCFVTQRRKQKQQQTMHQMAAMSTQEYYVDYKPNHIEGALVIINDYGSCSCHQQP
SRECEV

>HsLRRTM2
MGLHFKWPLGAPMLAAIYAMSMVLKMLPALGMACPPKCRCEKLLFYCDSQGFHSVPNATDKGSLGLSLRHNHITELERDQFASFSQLTWLH
LDHNQISTVKEDAFQGLYKLKELILSSNKIFYLPNTTFTQLINLQNLDLSFNQLSSLHPELFYGLRKLQTLHLRSNSLRTIPVRLFWDCRS
LEFLDLSTNRLRSLARNGFAGLIKLRELHLEHNQLTKINFAHFLRLSSLHTLFLQWNKISNLTCGMEWTWGTLEKLDLTGNEIKAIDLTVF
ETMPNLKILLMDNNKLNSLDSKILNSLRSLTTVGLSGNLWECSARICALASWLGSFQGRWEHSILCHSPDHTQGEDILDAVHGFQLCWNLS
TTVTVMATTYRDPTTEYTKRISSSSYHVGDKEIPTTAGIAVTTEEHFPEPDNAIFTQRVITGTMALLFSFFFIIFIVFISRKCCPPTLRRI
RQCSMVQNHRQLRSQTRLHMSNMSDQGPYNEYEPTHEGPFIIINGYGQCKCQQLPYKECEV

>GgLRRTM2
MQPPMYSKEWMACPPKCRCEKLLFYCDSQGFHSVPNTTEKGSLGLSLRHNFITELERDQFASFSQLTWLHLDHNQIATVREDSFQGLYKLK
ELVLSSNKIFHLPNTTFSQLLNLQNLDLSFNQLSSLHPELLYGLRKLQTLHLRSNSLRTIPVRLFWDCRSLEFLDLSTNRLRSLARNGFAG
LIKLRELHLEHNQLTKINFAHFLRLSSLHTLFLQWNKISNLTCGMEWTWGTLEKLDLTGNDIKAIDLTVFETMPNLKTLLLDNNKLSTLDS
KILRSLPSLSTVGLSGNLWECSARICALAAWLGAFRGRWEHPILCHGPDHAQGEDILDAVHGFQLCWNASTAAPSAAPTDAPTTEYTKGIS
SSHFHMGDKEIPTTAGMVVTTEEPFPEPNNAIFTQRVITGTMALLFSFFFIIFIVFISRKCCPPTLRRIRQCSMIQNHRQLRSQTRLHMAN
MSDQGPYNEYEPTHEGPFIIINGYGQCKCQQLPYKECEV

>LcLRRTM2
MGLHSKWPLGSPTSIALYAISMLLKTLPALGMTCPLNCRCEELLFYCDSQGFQFIPSNIDKGSLGLSLRHNSILELQNNEFGSFTQLTWLH
LDHNLISTVHEDAFQGLYKLKELILSANKIVHLSNTTFSQLINLQNLDLSFNQMTSLELELFHGLRKLQTLHLRSNSLRTIPVRIFWDCRN
LEFLDLSNNRLRSLSRNGFAGLIKLKELHLEHNQLTKINFAHFPRLISLHTLFLQWNKISNLTCGMEWTWETLEKLDLTGNEIKYIDTNVF
VIMPNLKILLLDINKLTSLSSQVVNSWRALTIVGLSGNQWECTNKICALASWLSNFRGRWEHSIVCHSPEHTQGEDILDAVYGFQLCKNLS
TEVASIATGYTDATQPLEATTEYEISITSTEYHAWDTESPTTVGTTVTDEGSLEPDTTLLPQRVIVGTMALLFFFFLIIFILYLSRKCCPP
TVRRIRQCSIIQNQRQLRNQTRQPISDLSTQLPYNEYEPSHEEGALVIINGYGQCKCQQLPYKECEV

>DrLRRTM2
MGFHSRWPLVGQAPAALCVISMLLCLPPVSCTTCPQKCRCEDLQFYCDTQGLLAPPDGVDKGALGLSLRHNSISELSSDQFFGFTQLTWLH
LDHNQITTVHEDAFQGLYKLKDLNLSSNRITKLPNTTFIHLINLQILDLSFNQMTALEPELFHGLRKLQILHLRSNSLRTTPVRAFWDCRS
LEYLGLSNNRLRSLARNGFAGLIKLRELHLEHNHLTKINLAHFPRLVALQFLYLQWNKINNLTSTMEWKWTTLEKLDLTGNEIRVLIPEVF
ETLPSLKILLLDNNKLSNLDSQTMDMWKSLNVIGLSSNLWECTKRICNLATWLSTFKGRWEHSILCYSPEYAQGEEILDAVYGFQLCQNFS
APVVLTSTSTEAMLPDITSSLFGNMQTPTQDFYAEDFGSFTIVTTTTTTTQPPRTALATTMTVEGADVTEDYSAMDNTLLTQRVIIGTMAL
LFSFFLIIFVVYISRKCCPPTLRRIRHCSAIQNRRQMRTQQRQPMADLATQVPYNEYETSHEEGALVIINGYGQCKCQQLPYKECEV

>LoLRRTM2
MGFHSRWPLVGPAPVALCVIGMLLRLLPVSGTACPQKCRCEDLLFYCDSQGFQAPPDGVDKGALGLSLRHNSITELSPDQFYGFSQLTWLH
LDHNQIKTVREDAFQGLYKLKELVLSSNLISTLPNTTFIHLINLQNLDLSFNQMTSIEPELFHGLRKLHTLHLRSNSLRTTPVRVFWDCRS
LEFLDLSNNRLRSLARNGFAGLIKLRELHLEHNQLTRINLAHFPRLISLQTLFLQWNKINNLTCGMEWIWTTLEKLDLTGNEIHTLTPDVF
ETLPNLKVLLLDNNKLTSLDSQILDMWKSLSTVGLSSNLWECTKRICSLATWLSTFRGRWEHSILCHSPEYAQGEEILDAVYGFQLCQNFS
```

VAAAPTTSSPKATTPPEATSSVFGIIPTPTQDFYAEDITTITTTTTPSPTTSLTTTTVAGEPVTEEDFLEMDNTLLTQRVIIGTMALLFSF
FLIIFVVYISRKCCPPTLRRIRHCSALQNRRQMRTQARQTMADLATQVPYNEYEPAHEEGALVIINGYGQCKCQQLPYKECEV

>CmLRRTM2
xxxxxxxxxxxxxxxxxxxxxxxxxxxxxxxxxxxxxxxxxxxxxxxxxxxxxxxxxxxxTMHLRSNSLRTIPIRVFWDCRSLEFLDIS
NNRLRSLARNGFAGLIKLKELHLEHNQLTKINFAHFPRLISLQTLFLQWNKISVLLCGMEWTWDTLEKLDVTGNEIRTIEPMVFFTTPNLK
ILLLDSNKLTTVDAQIVNAWKSLTHIGLSANMWACNKSICALASWLSNFQGRWENSIVCASPEHTQGEDILDAVHGFQICRNLSAATTQIT
TGYTDATQQLEATEYVTKLTSIGSSTEDTENPTTETTTAAAATAEDIPEFDNTLFTHRIITGTMALLFSFFLIILVVYISRKCCPPTLRRI
RHCSMMQHRRQMRQARQPMADLSTQVPYNEYEPTHDEGALVIINGYGQCKCQQLPYKECEV

PLGGPTAVALCVMSMLLKMPPGSGTACPPKCRCEEMLFYCDSQGLQRVPNNIARGSIGLSLRHNSFVELQNDQFASFGQLVWLYLDHNQIS
VVQEDSFQGLFKLKELILSYNKIVRLPNTTFSQLINLQNLDVSFNQITSLQPEQFHSLRKLQTMHLRSNSLRTIPIRVFWDCRSLEFLDIS
NNRLRSLARNGFAGLIKLKELHLEHNQLTKINFAHFPRLISLQTLFLQWNKISVLLCGMEWTWDTLEKLDVTGNEIRTIEPMVFFTTPNLK
ILLLDSNKLTTVDAQIVNAWKSLTHIGLSANMWACNKSICALASWLSNFQGRWENSIVCASPEHTQGEDILDAVHGFQICRNLSAATTQIT
TGYTDATQQLEATEYVTKLTSIGSSTEDTENPTTETTTAAAATAEDIPEFDNTLFTHRIITGTMALLFSFFLIILVVYISRKCCPPTLRRI
RHCSMMQHRRQMRQARQPMADLSTQVPYNEYEPTHDEGALVIINGYGQCKCQQLPYKECEV

>HsLRRTM3
MGFNVIRLLSGSAVALVIAPTVLLTMLSSAERGCPKGCRCEGKMVYCESQKLQEIPSSISAGCLGLSLRYNSLQKLKYNQFKGLNQLTWLY
LDHNHISNIDENAFNGIRRLKELILSSNRISYFLNNTFRPVTNLRNLDLSYNQLHSLGSEQFRGLRKLLSLHLRSNSLRTIPVRIFQDCRN
LELLDLGYNRIRSLARNVFAGMIRLKELHLEHNQFSKLNLALFPRLVSLQNLYLQWNKISVIGQTMSWTWSSLQRLDLSGNEIEAFSGPSV
FQCVPNLQRLNLDSNKLTFIGQEILDSWISLNDISLAGNIWECSRNICSLVNWLKSFKGLRENTIICASPKELQGVNVIDAVKNYSICGKS
TTERFDLARALPKPTFKPKLPRPKHESKPPLPPTVGATEPGPETDADAEHISFHKIIAGSVALFLSVLVILLVIYVSWKRYPASMKQLQQR
SLMRRHRKKKRQSLKQMTPSTQEFYVDYKPTNTETSEMLLNGTGPCTYNKSGSRECEV

>GgLRRTM3
MGFNVIRLLSGSAVALVIAPTVLLTMLSSAERGCPKGCRCEGKMVYCESQKLQEIPSSISAGCLGLSLRYNSLQKLKYNQFKGLNQLTWLY
LDHNHISNIDENAFNGIRRLKELILSSNRISYFLNNTFRPVTNLRNLDLSYNQLQSLGSEQFRGLRKLLSLHLRSNSLRTIPVRIFQDCRN
LELLDLGYNRIRSLARNVFAGMIRLKELHLEHNQFSKLNLALFPRLVSLQNLYLQWNKISVIGQTMSWTWSSLQRLDLSGNEIEAFSGPSV
FQCVPNLQRLNLDSNKLTFIGQEILDSWISLNDISLAGNIWECSRNICSLVNWLKSFRGLRENTIICASPKELQGVNVVDAVKNYSICGKS
TTERFELARAPPKPTFKPKLTRPKHDSKPPLPPTIGATESSSEPEHDTEHISFHKIIAGSVALFLSVLVILLVIYVSWKRYPASMKQLQQR
SLMRRHRKKKRQSLKQMTPSTQEFYVDYKPTNTETSEMLLNGAGPCTYNKSGSRECEV

>XtLRRTM3
MGVNPYRMLSGSAVFVLFALLVIMAVSSSGERGCPKGCRCDGKMFYCESQKLQEIPSSVSAGCIGLSLRYNSLHVLKFNQFKGLNQLTWLY
LDHNHISSIDENAFNGIRRLKELILSSNRISLLLNNTFRPVTNLRNLDLSYNILQTLGPGHFKGLRKLQSLHIRSNLLRTIPVRIFQDCRN
LEILDLGYNRIRSLARNVFTGMIRLKELHLEHNHFSRLNLALFPRLVSLQSLYLQWNRISLIGQTMSWTWHSLQRLDLSGNEIEAFSGPSV
FQCVPNLQRLNLDSNKLTFIGQDILDTWRSVTDIGLAGNIWECTRNICSLVNWLKSFKGLRDNTILCASPKELQGVNVMQAVGNYNVCSKS
IIENRELIIARTPTVLLSVISTKQENNYSVPPTVGTTESVYESNEGATELISLHKIIAGTVALFLSVIVISLVIYVSWRRSPAGKRHLQQG
SLMKNHRHQKSPSLTQMTPSAQEFYVDYSPGSKETCEMVPNGSAACTYNQSGSRECEV

>LcLRRTM3
MGFNVIRLLSGSAVVLVVAPTVLLTMLSSAERGCPKGCRCEGKMIYCESQKLQEVPPGISFGCLGLSLRYNSLQKLKYNQFKGLNQLTWLY
LDHNHIGNIDENAFNGIRRLKELILSSNKIIHILNNTFRPVTNLRNLDLSYNKLQSLGSEQFRGLRKLQSLHLRSNSLRNIPVRIFQDCRN
LEFLDLGYNRIRSLARNVFAGLIRLKELHLEHNQFTKLNLALFPRLLSLQFLYLQWNKISVIGQTMSWTWSSLQKLDLSGNEIEAFSGPSV
FQCVPNLQTLNLDSNKLTFIGQEILDSWISLTSISLSGNIWECSRNICSLVNWLKSFKGWKENTVICASPKELQGVNVIDAIKNGSICGKS
TTERLDSTRALAKPTVKPKSTKPKHESKPPLPSAMGVPESSSDADHETEHISFHKIIAGSVALFLSVLVILLVIYVSWKRYPASMKQLQHR
SLVRRRRKKKRQSLKQMTPTTQEFYVDYKPTNTETSEMLLNGTGPCTYNKTGSRECEV

>LoLRRTM3
MGFSAIRLLSRSAAVLVIAPTVLLTMLSSAERGCPKGCRCEGKMIYCESQKLLEIPQGISGGCLGLSLRYNSLQSLKYNQFKGLNQLTWLY
LDHNHISNIDENAFNGIRRLKELILSSNKIKQLLNNTFRPVTNLRNLDISYNQLSSLGTEQFRGLRKLQSLHLRSNNLRNIPVRIFQDCRN
LEFLDMGYNRIRSLARNVFAGLIRLKELHLEHNQFGKLNLALFPRLVSLQFLYLQWNKISVIGQTMSWTWSSLQKLDLSGNEIEAFSGPSV
FQCVPNLQTLNLDSNKLTFIGQEIVDSWISLSTISLTGNIWECSQNICSLVNWLKSFRGWRENTIICASPKELQGVNVMEAVKNYSICSKS
TFEKLDTRVPVNPPLKPTPSRPKQETKHEIPFLPTPSIPESSTDADHDTEHISFHKIIAGSVALFLSVLVILLVIYVSWKRYPASMKQLQQ
KSLMRRRRKKKRQSLKQMTPTTQEYYVDYKPTNTETSEMLLNGTGPCTFSKTGSRECEV

>CmLRRTM3
QCVPNLQTLHLDSNKLTTLGQEILDSFISLTSISLSGNIWECNRNICSLVNWLKMFKGRRESTMICAGPKQLQGEHVLEAVDHYNICVKKH
KITTQGASDVSKALPKPTFKPNTERPKYESKPLPATLVSPESSSDADVVEHISFHKIIAGSVALFLSVLVILLVIYVSWKRYPASVKQLQQ
RSLVRRRRKKKRQSLRQMTPTTQEYYVDYKPTNSEVAEMLMNGTVPCTYTKSGSRECEV

>HsLRRTM4
MGFHLITQLKGMSVVLVLLPTLLLVMLTGAQRACPKNCRCDGKIVYCESHAFADIPENISGGSQGLSLRFNSIQKLKSNQFAGLNQLIWLY
LDHNYISSVDEDAFQGIRRLKELILSSNKITYLHNKTFHPVPNLRNLDLSYNKLQTLQSEQFKGLRKLIILHLRSNSLKTVPIRVFQDCRN
LDFLDLGYNRLRSLSRNAFAGLLKLKELHLEHNQFSKINFAHFPRLFNLRSIYLQWNRIRSISQGLTWTWSSLHNLDLSGNDIQGIEPGTF
KCLPNLQKLNLDSNKLTNISQETVNAWISLISITLSGNMWECSRSICPLFYWLKNFKGNKESTMICAGPKHIQGEKVSDAVETYNICSEVQ
VVNTERSHLVPQTPQKPLIIPRPTIFKPDVTQSTFETPSPSPGFQIPGAEQEYEHVSFHKIIAGSVALFLSVAMILLVIYVSWKRYPASMK
QLQQHSLMKRRRKKARESERQMNSPLQEYYVDYKPTNSETMDISVNGSGPCTYTISGSRECEV

>GgLRRTM4
MGFHLIKQLRGMSVVLVLLPVVLFVVLAGAQRACPKNCRCDGKIVYCESHAFRDIPQNISGGSQGLSLRYNSIQKLKSNQFAGLNQLIWLY
LDHNYINSVDEDAFQGIRRLKELILSSNKITHLHNKTFHPVPNLRNLDLSYNKLQVLQSEQFKGLRKLLILHLRSNSLKTVPIRVFQDCRN
LDFLDLGYNRLRSLSRNAFAGLLKLTELHLEHNQFSKINFAHFPRLFNLRSIYLQWNRIRSISQGLTWTWSSLHNLDLSGNDITGIEPGTF
QCLPNLQKLNLDSNKLTNISQETINTWISLISITLSGNMWECTRSICPLFTWLKNFKGNKESTMICAGPKQIQGEKVSDAVETYNICAEIQ

VVVTERSYQAPRTPQRPVFIPKPTVSKLESNQPTSVVPSPSTDLPTPAVEPEYEHVSFHKIIAGSVALFLSVAMILLVIYVSWKRYPASMK
QLQQHSLMKRRRKKARESERQMNSPLQEYYVDYKPTNSETMDVSVNGSGPCTYTISGSRECEV

>XtLRRTM4
MGFQLMNQLKGMSLTLLLLPALIVVMVVGAQKTCPKNCRCDGKIVYCESHAFRDIPQNISGGSQGLSLRYNSIKMLKSHQFSGLNQLVWLY
LDHNYISTVDEDAFQGIRRLKELILSSNKISYLGNSTFHPVPNLRNLDLSYNKLQTLQSEQFKGLRKLLILHLRSNSLKTVPVRVFQDCRN
LDFLDLGYNRLRSLSRNAFAGLLKLKELHLEHNQFSKVNFAHFPRLFNLRSLYLQWNKIRSISQGLTWTWSALQNLDLSGNDIQNLEPGTF
QCLPNLQKLNLDSNKLTNVTQETLNAWISLTSITLSGNIWECTKKICPLVFWLKNFKGNKESIMICASPKNMQGEKVTDAVETYNICNETP
VLATERAYPTTKPPPKPQFTPKPTIYKLESVSSTPYTSSPSPALQTPMAEQEYEHVSFHKIIAGSVALFLSVAMILLVIYVSWKRYPASMK
QLQQSSMMKRRRKKARESERQINSPLQEYYVDYKPSNTESMDVLVNGSGPCAYTISGSRECEV

>LcLRRTM4
MGFHLIKQLRGTSVMLVLLPMVLLVILADAERVCPKNCRCDGKIVYCESHAFKDIPHNISGGSQGLSLRYNSIQKLKSNQFSGLNQLVWLY
LDHNYITSVDEDAFQGIRRLKELILSSNKITQLQNKTFHPIPNLRNLDLSYNKLQALQSEQFKGLRKLLSLHLRSNSLKTVPIRVFQDCRN
LEFLDLGYNRLRSLSRNAFAGLLKLTELHLEHNQFSKVNFAHFPRLYSLRSLYLQWNRIKSINQGLTWTWSSLQKLDLSGNDIQGIEPGIF
QCLPNLQTLNLDSNKLTNISQETVNAWISLTTISLSGNVWECNRSICALVLWLKNFKGNKESTMICAGPKAIQGEKVMEAVDSYNICAEIQ
VVVTDRPPQAPKASQKSTFILKPTIPEQESNPQVPTTPSPSPVLQTPGSEQEYEHVSFHKIIAGSVALFLSVAMILLVIYVSWKRYPASMK
QLQQHSLMKRRRKKARESERQMSSPLQEYYVDYKPTNSETMDVLVNGSGPCTYTISGSRECEV

>DrLRRTM4_1
MGSLVRSRWLMIPLLVQAWLLASQGRVREKPCPRSCRCDGKIVYCESNAFRDVPKNVSIGCQGLSLRYNSLVNLRAGQFASLSQLVWLYLD
HNYISGVDKQAFQGVRRLKELILSSNKITHLENSTFHSIPNLRNLDLSYNKLQILQPNQFQGLRKLLSLHIRSNSLKTVPMRVFQDCRNLE
FLDLGYNRLRSLTRNAFAGLLKLTELHLEHNQFSKINFSHFPRLNNLRALYLQWNRIKSITQGITWTWTSLQKLDLSGNDLQVLDSSIFQC
LPNLQTLNLDSNKLSNVSQEAISAWISLTTISLAGNIWECNPSLCPLVAWLRNFKGNKETTMICAGPKEIQGEKVIEAIDTHGICKTTPTP
YLTMSSTVSSPSKAKRFPLPTMFRVNEKITRNNAVAPSPTAASPPVPEQDFEHVSFHKIIAGSVALFLSVAMILLVIYVSWKRYPSSMKQL
QQHSMVRKRRKKARETERTLSSPLQEYYVDYKPTNSETMDVLVNGTGPCTYTISGSRECEV

>DrLRRTM4_2
MGFAMRHRWFMVPLLMQAWLSATPCFGERPCPRSCRCDGKIVYCESSAFRDVPKNLSGGCQGLSLRYNSLASLKAGQFVGLNQLIWLYLDH
NYIANVDARAFQGIRRLKELILSSNKITLLHNTTFHLVPNLRNLDLSYNKLQALQPGQFQGLRKLLSLHMRSNSLKNLPSRLFQDCRNLEF
LDLGYNRLRSITRNSMSGLLKLTELHIEHNQFSKINFFNFPRFYNLRALYLQWNRIRTMSEGLTWMWTSLQKLDLSGNDLQEIDAEVYRAM
PNLQTLNLDSNKLVNVSQEAVDAWTSLTAISLAGNMWDCGPVVCPLVAWMRTFRGNKEINMICASPKEVQGEKVLDAVDANGVCRIAPATP
STIFVPSTPFVAFSQTPASSLVPTLKSGDSRRVHGTSLEGSTSSVPSQPDVPPQEQDFEPVSFHKIVAGSVALFLSVAMILLVIYVSWKRY
PSSVKQLQENSAAARKRRKKSRETERTLSTPLQEYYVDYKPNNTEGTDVLVNGTGPCTYTISGSRECEV

>DrLRRTM4_3
MGPLLWEGRLSCFLLHASVLLLLSKGERMCPVSCRCEGKIVYCESGGFQDVPENISLGCQGLSLRYNSLLLLLPYQFAHLNQLVWLYLDHN
SISTVDRLAFQGLRRLKELILSSNKIAQLQNGTFETVPNLRNLDLSYNQMQDLVPGHFHGLRKLQNLHLRSNNIRSIPVRTFMECRSLEFL
DLGYNRLRTITRTTFLGLLRLTELHLEHNQFSRINFFLFPRLLNLQALYLQWNRIRTVVQGSPWTWHTLQKLDLSGNEIQILDPAIFRCLP
NLHTLNLESNKISNVSLEVVSSWISISTINLAGNIWDCSSSICPLVSWLRNFRGTRDASIICSTPKSLQGERVMDAVRNNSVCEPRTTTRQ
LSQVTHIQRLTPKVLLPVRPEKEFLTVTPSSLPFTPEPEFEHMTFHKIIAGSVALFLSVSLILLVIYVSWRRYPNSMRQLQQHSIRRKRRK
KTREPEHNINSQLQEYYLSYNHANAETMDSLVNGACTCTISGSRECEV

>DrLRRTM4_4
MGSLACDMRLAGLLLLQASSLLLFSSGERMCPYSCHCEGKIVHCESSAFQDVPENISVSCQGLSLRYNDLHTMLPYQFAHLNQLLWLYLDH
NQIMFVDSRAFQGVRRLKELILSSNRISQLHNVTFHGVPNLRSLDLSYNKLQELQPGQFYGLRKLQNLHLRSNGLTAIPVRAFLECRSLEF
LDLGYNRLRVLTRTAFLGLSRLMELHLEHNQFSRINFFLFPRLANLRALYLQWNRIRAVNQGLPWSWYTLQRLDISGNEIQVLDPVVFQCL
PNLQVLNLESNKLANVSQEVVAAWISLTTISLAGNMWDCGPGICPLVVWLKNFRGTKDTNIICSSPKHLQGEKVIEASKNYIDCDDYEITA
QSPLYLQTFDPNYDLALEPTEPTMAPTTPPPPLPSPASEAPFPPPQPRPPHRPTFPSRTNTDPRDSHQRTPSSFDSSLVTPVPEQDNVSFH
KVVMGGVALFFSVSLVLSVIYVSCKRYPGATRLLQQSSVGRKRRKKSQEPEQNLSSQLQEYYMSYNPAATPEALEVLGNGTGTCTCTISGS
RECEV

>LoLRRTM4
MGFLMRGGWLAVSLLLQAWLLVSLCLGERPCPRSCRCDGKIVYCESNAFRDVPNNVSAGCQGLSLRYNSLLSLRAGQFSGLNQLIWLYLDH
NYINSVDSNAFQGIRRLKELILSSNKITELHNTTFHSVPNLRNLDLSYNKLQALQPGQFQGLRKLLSLHLRSNSLKSVPVRVFQDCRNLEF
LDLGYNRLRSLTRNAFAGLLKLTELHLEHNQFSKINFSHFPRLFNLRALYLQWNRIRSISQGLSWTWTSLQKLDLSGNDIQVAEASIYQCL
PNLQTLNLDSNKLSNVSQEAVAAWISLTTISLAGNVWDCSPSICPLVSWLKNFKGNKETTMICAGPKEVQGEKVMDAVEAYSICKETPAPS
TEKPLPPAQTPPKPRLPPKPTVPKLGEEESQGVLPSPSPSGASTLSPEPEFEHVSFHKIIAGSVALFLSVAMILLVIYVSWKRYPSSMKQL
QQHSMVRKRRKKAREAERTLSSPLQEYYVDYKPTNSETMDVLVNGTGPCTYTISGSRECEV

>CmLRRTM4
IIPGVFRCVPNLQSLHLDSNKLTTVSEEILNSWASLTTVSLSSNIWECNRNICALVSWLKGFKGYRESAMICAGPKPVQGQNVLEAIQLHK
TCSGLPKASTTQRPWTSQATRRSPRLPTRPAPAPAGDRPPAAHTARPPNSSSAEPELDFEHVSLHKIVAGSAALFLSVAMILLVIYVSWKR
YPASVKHLQQRSLVKRRRKKEREHERQMNTPLQEYYVDYKPSNSETVDVLANGSETCTYPRAISRECEV

xxxxIGVSLSLSLSPNTGPWVLSVASLGMLATISVAARVCPRTCRCDGKIVYCESQALLEIPPNISSGSLGLSLRYNSIKSLRPSQFSGLN
QLTWLYLDHNHLQGVDREAFQGIRRLKELILSSNKIARLHNVTFHPVPNLRNLDLSYNKLHLLQPQQFKGLRKLQSLHLRSNSLKVIPVRL
FQDCRNLEFLDLGYNRLRNLARNVFAGLMKLTELHLEHNQFSKINLSHFPRLSSLRTLYLQWNRIRVFSQGMSWTWSSLQKLDLSGNEIQA
IIPGVFRCVPNLQSLHLDSNKLTTVSEEILNSWASLTTVSLSSNIWECNRNICALVSWLKGFKGYRESAMICAGPKPVQGQNVLEAIQLHK
TCSGLPKASTTQRPWTSQATRRSPRLPTRPAPAPAGDRPPAAHTARPPNSSSAEPELDFEHVSLHKIVAGSAALFLSVAMILLVIYVSWKR
YPASVKHLQQRSLVKRRRKKEREHERQMNTPLQEYYVDYKPSNSETVDVLANGSETCTYPRAISRECEV

>PmLRRTM_A
MGLILFRVLSGPRTRLMIFTAAFILSFPTVEGACSKSCKCDVKIVYCESLGLKGLPRNISAGTLGLSLRYNSLQELNYNQFASFRQLTWLY
LDHNYVHTVDAHAFQGIRRLKELILSSNRISNLANGTFRPISNLRNLDLSYNQLQILDPDLFRGLRKLQSLHLRSNTIKTMPVRIFQDCRN

LEFLDLGYNRLRSLARNAFAGLTRLTELHMEHNQFSKVNFAHFPRLMSLRHLYLQGNKISEMTQGMVWIWSTLQILDLSGNELQSLRANAF
EYVPNLQTLRLDSNKFTTLTHEVLSSLKSLNLISLSGNLWECDRKLCALGSWLTTFGIRREKNMICAGPKHLEGENVMEAIDNYNICGERP
RVTAEPTAPAPKPGSRPTGWLKPVVESRIEWGEPIVLTTDPTADFPVHPPPPPLDPDLEAVSLHKVIAGCVALILSVLVILLVIYISWRRY
PASMRQLQQQGPLAHHRRQKKRRRRGASNERAAQPTIPLEYYVDYKPTNSEATEGLANGTTTHCSAFIVSAMQECEV

>PmLRRTM_B
MGLRVFRLWIGPFAVLARSLLLITLHSGECACPRICRCEGKFVYCESMALREIPMNITTGSLGLSLRYNSLKSLPENHFSGLKQLTWLYLD
HNHIQLVAEGAFVGARRLKELTLSSNKLIRLPNATFHPLVNMRSLDISFNQLQMLEAGQFRGLRKLQYLHMRSNQLRTVPVRIFQDCRSIE
LLDLGYNRLRVLARNAFTGLGRLTELHLEHNQFAKLNLAHFPRLISLRTLYLQWNRISVLMQGMAWTWSSLQTLDLSGNEIQLIGPGFFQG
LSKLHNLRLDSNRLAAVSSETMSAWTGLSSLNLAGNSWQCNQNICSIAIWLNSFQGTQEGVMRCGSPQNFLGQSIIDVAFKDGICRTSTVS
AAEIPKVSTTITLPQTNRAANNEPGNTKTTISTPIPTSASPLPSQSWETGSDSEHTSLYKILAGSVALVLSVLVMLLVVYVTWKRYPASLG
QEQREQHQQHQTLQGRRGRRPARKIEGKATRQHDGAILAQEYYVDYKPSNSEAGDLLGNGNAAFMMQSMGNRDCQV
