# Supplementary material for: Origin and Loss of Nested LRRTM/α-Catenin Genes during Vertebrate Evolution
Source: PLoS One. 2014 Feb 24;9(2):e89910. doi: 10.1371/journal.pone.0089910 (PMC3933685; doi:10.1371/journal.pone.0089910)
Supplement: Table S2 — Accession numbers for the α-catenin sequences used. (PDF) [file pone.0089910.s009.pdf]

Table S2. Accession numbers and assembled amino-acid sequences of α-catenins.

Protein	Refseq	Ensembl	Elefant shark genome
HsCTNNA1	NP_001894.2		
GgCTNNA1	XP_414513.1		
XtCTNNA1		ENSXETP00000018423	
LcCTNNA1	XP_006003759.1		
DrCTNNA1	NP_571531.1		
LoCTNNA1		ENSLOCP00000014576	
CmCTNNA1			AAVX01038267.1<br>AAVX01215805.1<br>AAVX01270210.1<br>AAVX01038266.1<br>AAVX01071410.1
HsCTNNA2	NP_004380.2		
GgCTNNA2	NP_990467.1		
XtCTNNA2	NP_001090774.1		
LcCTNNA2	XP_006004641.1<br>XP_005998291.1		
DrCTNNA2	XP_005160129.1		
LoCTNNA2		ENSLOCG00000002534<br>ENSLOCT00000003019<br>LG4:6180092:6180172	
CmCTNNA2			AAVX01050520.1<br>AAVX01159160.1<br>AAVX01263085.1<br>AAVX01077370.1
HsCTNNA3	XP_005269774.1		
GgCTNNA3	NP_001186465.1		
LcCTNNA3	XP_006003252.1<br>XP_006010356.1<br>NW_005819914.1<br>NW_005821299.1		
LoCTNNA3		ENSLOCP00000014271<br>ENSLOCP00000014285	
CmCTNNA3			AAVX01289477.1<br>AAVX01235657.1<br>AAVX01368489.1<br>AAVX01228089.1<br>AAVX01238450.1<br>AAVX01238496.1<br>AAVX01060192.1
PmCTNNA			ENSPMAP00000002805<br>ENSPMAP00000002805
BfCTNNA	XP_002608335.1		
CiCTNNA	XP_002131748.1		
DmCTNNA	NP_524219.1		

```
>HsCTNNA1
MTAVHAGNINFKWDPKSLEIRTLAVERLLEPLVTQVTTLVNTNSKGPSNKKRGRSKKAHVLAASVEQATENFLEKGDKIAKESQFLKE
ELVAAVEDVRKQGDLMKAAAGEFADDPCSSVKRGNMVRAARALLSAVTRLLILADMADVYKLLVQLKVVEDGILKLRNAGNEQDLGIQ
YKALKPEVDKLNIMAAKRQQELKDVGHRDQMAAARGILQKNVPILYTASQACLQHPDVAAYKANRDLIYKQLQQAVTGISNAAQATAS
DDASQHQGGGGGELAYALNNFDKQIIVDPLSFSEERFRPSLEERLESIISGAALMADSSCTRDDRRERIVAECNAVRQALQDLLSEYM
GNAGRKERSDALNSAIDKMTKKTRDLRRQLRKAVMDHVSDSFLETNVPLLVLIEAAKNGNEKEVKEYAQVFREHANKLIEVANLACSI
SNNEEGVKLVRMSASQLEALCPQVINAALALAAKPQSKLAQENMDLFKEQWEKQVRVLTDAVDDITSIDDFLAVSENHILEDVNKCVI
ALQEKDVDGLDRTAGAIRGRAARVIHVVTSEMDNYEPGVYTEKVLEATKLLSNTVMPRFTEQVEAAVEALSSDPAQPMDENEFIDASR
LVYDGIRDIRKAVLMIRTPEELDDSDFETEDFDVRSRTSVQTEDDQLIAGQSARAIMAQLPQEQKAKIAEQVASFQEEKSKLDAEVSK
WDDSGNDIIVLAKQMCMIMMEMTDFTRGKGPLKNTSDVISAAKKIAEAGSRMDKLGRTIADHCPDSACKQDLLAYLQRIALYCHQLNI
```

CSKVKAEVQNLGGELVVSGVDSAMSLIQAAKNLMNAVVQTVKASYVASTKYQKSQGMASLNLPAVSWKMKAPEKKPLVKREKQDETQT
KIKRASQKKHVNPVQALSEFKAMDSI

>GgCTNNA1
MTAVTAGNVNFRWDPKSLEIRTLAVERLLEPLVTQVTTLVNTSNKGPSNKKRGRSKKAHVLAASVEQATENFLDKGDKIAKESQFLKE
ELVAAVEDVRKQGDLMKSASGEFADDPCSSVKRGNMVRAARALLSAVTRLLILADMADVYKLLVQLKVVEEGILKLRNAGTEQDLGIQ
YKALKPEVDKLNIMAAKRQQELKDVGHRDQMAAARGILQKNVPILYTASQACLQHPDVAAYKANRDLIYKQLQQAVTGISNAAQATAS
DDAAQQQGGGGELAYALNNFDKQIIVDPSTFSEERFRPSLEERLESIISGAALMADSSCTRDDRRERIVAECNAVRQALQDLLSEYMG
NAGRKERSDALNSAIDKMTKKTRDLRRQLRKAVMDHVSDSFLETNVPLLVLIEAARNGNEKEVKEYAQVFREHANKLIEVANLACSIS
NNEEGVKLVRMSASQLEALCPQVINAALALAAKPQSKLAQENMELFKEQWEKQVRVLTDAVDDITSIDDFLAVSENHILEDVNKCVIA
LQEKDVDGLDRTAGAIRGRAARVIHVVTSEMDNYEPGVYTEKVLEATKLLSNTVMPRFTEQVEAAVEALSSDPAQPMDENEFIDASRL
VYDGIRDIRKAVLMIRTPEELDDSDFETEDFDVRSRTSIQTEDDQLIAGQSARAIMAQLPQEQKAKIAEQVASFQEEKSKLDAEVSKW
DDSGNDIIVLAKQMCMIMMEMTDFTRGKGPLKNTSDVISAAKKIAEAGSRMDKLGRTIADHCPDSACKQDLLAYLQRIALYCHQLNIC
SKVKAEVQNLGGELVVSGVDSAMSLIQAAKNLMNAVVQTVKASYVASTKYQKSQGMASLNLPAVSWKMKAPEKKPLVKREKQDETQTK
IKRASQKKHVNPVQALSEFKAMESI

>XtCTNNA1
MTLNTGNINFKWDPKSLEIRTLAVERLLEPLVTQVTTLVNSSNKGPSNKKKGRSKKAHVLAASVEQATQNFLDKGDKIAKESQFLKEE
LIAAVEDVRKQGEQMRSASGEFADDPCSSVKRGNMVRAARALLSAVTRLLILADMADVYRLLVQLKVVEEGIVKLRNAGTEQDLGIQY
KALKAEVDKLNVMTAKRQQELKDIGHRDQMAAARGILQKSIPILYTASQACLQHPDVAAFKANRDLIYKQLQQAVTGISNAAQATSSE
ETAHQQGGGELAVALNNFDKQIIVDPLGFSEERFRPSLEERLESIISGAALMADSSCTRDDRRERIVAECNSVRQALQDLLSEYMGNT
GRKERSDALNSAIDKMTRKTRDLRRQLRKAVMDHVSDSFLETNVPLLVLIEAAKNGNEKEVKEYAQVFREHANKLIEVANLACSISNN
EEGVKLVRISAGHXESMCPQVINAALALAAKPHSKMAQETMDLFKEQWERQVRVLTDAVDDITSIDDFLAVSENHILEDVNKCVIALQ
EKDVDGLDRTAGAIRGRAARVIHVVTSEMDNYEPGVYTEKVLEATKLLSNTGIKRIEMLCNTPEELDDSDFETEDFDVRSRTSVQTED
DQLIAGQSARAIMAQLPQEQKAKIAEQVASFQEEKSKLDAEVSKWDDNGNDIIVLAKQMCMIMMEMTDFTRGKGPLKNTSDVISAAKK
IAEAGSRMDKLGRTIADHCPDSACKQDLLAYLQRIALYCHQLNICSKVKAEVQNLGGELVVSGVDSAMSLIQAAKNLMNAVVQTVKAS
YVASTKYQKSQGMASLNLPAVSWKMKAPEKKPLVKREKQDETQTKIKRASQKKHVNPVQALSEFKAMESI

>LcCTNNA1
MTNVHTPNVNFKWDPRNLEIRTLAVERLLEPLVTQVTTLVNSSNKGPSNKKKGRSKKAHVLAASVEQATQNFLEKGDKIAKETQFLKE
ELTAAVEDVRKQGETMKGASGEFADDPCSSVKRGNMVRAARALLSAVTRLLILADMADVYKLLIQLKVVEDNLLKVRNAGTEQELGIQ
YKALKPEVDKLIAMAAKRQQELKDINHRDQMAAARGVLQKNIPILYTASQACLQHPDVAAYKANRDLIYKQLQQAVTGISNAAQATAS
DDLTVSQQSGGGGGELASALNNFDNQIIVDPLNFSEERFRPSLEERLESIISGAALMADSSCTRDDRRERIVAECNAVRQALQDLLSE
YMGNAGRKERSDALNSAIDKMTKKTRDLRRQLRKAVMDHVSDSFLETNVPLLVLIEAAKNGNEKEVKEYAQVFREHANKLIEVANLAC
SISNNEEGVKLVRMSASQLEALCPQVINAALALAAKPQSKVAQENMDLYKDQWERQVRVLTDAVDDITSIDDFLSVSENHILEDVNKC
VIALQEKDVDGLDRTAGAIRGRAARVIHVVTSEMDNYEPGVYTEKVLEATKLLSNTVMPRFTEQVEAAVEVLSTNPSQPVDENEFIDA
SRLVYDGVRDIRKAVLMIRTPEELDDSDFETEDFDVRSRTSVQTEDDQLIAGQSARAIMAQLPQEQKAKIAEQVAIFEEEKIKLDAEV
SKWDDSGNDIIVLAKQMCMIMMEMTDFTRGKGPLKNTSDVISAAKKIAEAGSRMDKLGRTIADHCPDSACKQDLLAYLQRIALYCHQL
NICSKVKAEVQNLGGELVVSGVDSAMSLIQAAKNLMNAVVQTVKASYVASTKYQKSQGMASLNLPAVSWKMKAPEKKPLVKREKQDEA
QTIIKRSSQKKHVNPVQALSEFKAMDSI

>DrCTNNA1
MTSINTANINFKWDPKSLEIRTLAVERLLEPLVTQVTTLVNSSNKGPSNKKKGRSKKAHVLAASVDEATQNFLEKGEKIAKESQFLKE
ELTAAVEDVRKQGESMKMASGEFAEDPCSSVKRGNMVRAARALLSAVTHLLVLADMADVYQLLLQLKLVEENLMKVRNAGTEQDLGIQ
YKALKPEVDKLNMMAAKRQQELKDVHHKDQMAAARGVLQRNVPMLYTASRACLQHPDVAAYKANRDLIYKQLQHAVSGISNAAQATSS
EDSSFSQTAGGGELAYALNNFDKQIIVDPLSFSEERFRPSLEERLESIISGAALMADSSCTRDDRRERIVAECNSVRQALQDLLSEYM
GNAGRKEKSDALNTAIDRMTKKTRDLRRQLRKAVMDHVSDSFLETNVPLLVLIEAAKNGNEKEVKEYAQVFREHANKLIEVANLACSI
SNNEEGVKLVRMAASQLETLCPQVINAALALAAKPNSKVAQDNMDLFKDQWEKQVRVLTDAVDDITSIDDFLCVSENHILEDVNKCVI
ALQEKDVDGLDRTAGAIRGRAARVVHVVTSEMDNYMPGVYTEKVLEATKLLTETVMPRFTEQVEAAVEALSANNGQPVDENEFIDASR
LVYDGVRDIRKAVLMIRTPEELDDSDFETEDFDSRSRTSVQTEDDQLIAGQSARAIMAQLPQEQKAKIAEQVASFQEEKSKLDAEVSK
WDDSGNDIIVLAKQMCMIMMEMTDFTRGKGPLKNTSDVISAAKKIAEAGSRMDKLGRAIADQCPDSACKQDLLAYLQRIALFCHQLNI
CSKVKAEVQNLGGELVVSGLDSAMSLIQAAKNLMNSVVSTVKASYVASTKYQKSQDMQSLNMPAISWKMKAPEKKPLVKREKLDDGQT
NKVKRSSQKKHINPVQALSEFKAMDSI

>LoCTNNA1
MTSINTANINFKWDPKSLEIRTLAVERLLEPLVTQVTTLVNSSNKGPSNKKKGRSKKAHVLAASVETATQNFLEKGDKIARESQFLKE
ELTAAVEDVRKQGESMKTASGEFADDPCSSVKRGNMVRAARALLSAVTHLLVLADMADVYKLLVQLKLVEENLVKVRNAGTEQELGIQ
YKALKPEVDKLNMMAAKRQQELKDVHHKDQMAAARGVLQRNIPMLYTASQACLQHPDVAAYKANRDLIYKQLQQAVSGISNAAQATAS
EDSALSQPGGGGELAYALNNFDKQIIVDPVNFSEERFRPSLEERLESIISGAALMADSSCTRDDRRERIVAECNSVRQALQDLLSEYM
GNAGRKERSDALNSAIDRMTKKTRDLRRQLRKAVMDHVSDSFLETNVPLLVLIEAAKNGNEKEVKEYAQVFREHANKLIEVANLACSI
SNNEEGVKLVRMAASQLETLCPQVINAALALAAKPNSKVAQDNMDLFKDQWERQVRVLTDAVDDITSIDDFLCVSENHILEDVNKCVI
ALQEKDVDGLDRTAGAIRGRAARVVHVVTSEMDNYEPGVYTEKVLEATKLLTDTVMPRFSEQVEAAVEALSANPAQPVDENEFIDASR
LVYDGVRDIRKAVLMIRTPEELDDSDFETEDFDVRSRTSVQTEDDQLIAGQSARAIMAQLPQEQKAKIAEQVASFQEEKSKLDAEVSK
WDDSGNDIIVLAKQMCMIMMEMTDFTRGKGPLKNTSDVISAAKKIAEAGSRMDKLGRTIADHCPDSACKQDLLAYLQRIALYCHQLNI
CSKVKAEVQNLGGELVVSGVDSAMSLIQAAKNLMNTVVSTVKASYVASTKYQKSQGMQSLNLPAISWKMKAPEKKPLVKREKQDDGQT
NKVKRSSQKKHINPVQALSEFKAMDSI

>CmCTNNA1
NINFKWDPKNLELRTLAVERLLEPXXXXXXXXXXXXXKGPSGKKKGRSKKAHVLAASVEQATQNFLEKGEQIAKESQDLKDELLAAVD
DVRKQGESMRSASSEFADDPCSSMKRGNMVRAARALLSAVTRLLILADMADVYKLLVQLKVVEETLVKVRNANNEQELGIHYKALKPE
VDKLNLMAAKRQQELKDIHHRDQMAAARGVLQKNIPMLYTASQACLQHPDVAAYKANRDLIYKQLTQAVTGISNAAQATASEDAHLGQ
QSSAGELADALNRFDKQIIVDPLSFSEERFRPSLEERLESIISGAALMADSSCTRDDRRERIVAECNAVRQALQDLLSEYMGNAGRKE
RGDALNAAIDKMTKKTRDLRRQLRKAVMDHVSDSFLETNVPLLVLIEAAKNGNEKEVKEYAQVFRDHASKLVEVANLACSISNNEEXV
KLVRMAASQLESLCPQVVINAALALAAKPQSKVAQDNMDLFKELWEKQVRVLTDAVDDITSIDDFLCVSENHILEDVNKCVIALQEKD
VDGLDRTAGAIRGRAARVVHVVTSEMDNYEPGVYTEKVLEATKLLSETVMPRFSEQVEAAVEALSANPAQQVDENEFIDASRLVYDGI
RDIRKAVLMIRTPEELEDDSDFEQEDYDVRSRTSVQTEDDQLIAGQSARAIMAQLPQEQKAKIAEQVASFEEEKIKLDAEVSKWDDSG
NDIIVLAKQMCMIMMEMTDFTRRGKGPLKNTSDVISAAKKIAEAGSRMDKLGRTIADHCPDSSCKQDLLAYLQRIALYCHQLNICSKV

KAEVQNLGGELIVSGVDSAMSLIQAAKNLMNAVVQTVKASYVASTKYQRSQGMAALNMPAVSWKMRAPEKKPLVKREKNDETHKVKRA
SQKKHVSPVQALSEFKAMDSI

>HsCTNNA2
MTSATSPIILKWDPKSLEIRTLTVERLLEPLVTQVTTLVNTSNKGPSGKKKGRSKKAHVLAASVEQATQNFLEKGEQIAKESQDLKEE
LVAAVEDVRKQGETMRIASSEFADDPCSSVKRGTMVRAARALLSAVTRLLILADMADVMRLLSHLKIVEEALEAVKNATNEQDLANRF
KEFGKEMVKLNYVAARRQQELKDPHCRDEMAAARGALKKNATMLYTASQAFLRHPDVAATRANRDYVFKQVQEAIAGISNAAQATSPT
DEAKGHTGIGELAAALNEFDNKIILDPMTFSEARFRPSLEERLESIISGAALMADSSCTRDDRRERIVAECNAVRQALQDLLSEYMNN
TGRKEKGDPLNIAIDKMTKKTRDLRRQLRKAVMDHISDSFLETNVPLLVLIEAAKSGNEKEVKEYAQVFREHANKLVEVANLACSISN
NEEGVKLVRMAATQIDSLCPQVINAALTLAARPQSKVAQDNMDVFKDQWEKQVRVLTEAVDDITSVDDFLSVSENHILEDVNKCVIAL
QEGDVDTLDRTAGAIRGRAARVIHIINAEMENYEAGVYTEKVLEATKLLSETVMPRFAEQVEVAIEALSANVPQPFEENEFIDASRLV
YDGVRDIRKAVLMIRTPEELEDDSDFEQEDYDVRSRTSVQTEDDQLIAGQSARAIMAQLPQEEKAKIAEQVEIFHQEKSKLDAEVAKW
DDSGNDIIVLAKQMCMIMMEMTDFTRGKGPLKNTSDVINAAKKIAEAGSRMDKLARAVADQCPDSACKQDLLAYLQRIALYCHQLNIC
SKVKAEVQNLGGELIVSGLDSATSLIQAAKNLMNAVVLTVKASYVASTKYQKVYGTAAVNSPVVSWKMKAPEKKPLVKREKPEEFQTR
VRRGSQKKHISPVQALSEFKAMDSF

>GgCTNNA2
MTSATSPIILKWDPKSLEIRTLTVERLLEPLVTQVTTLVNTSNKGPSGKKKGRSKKAHVLAASVEQATQNFLEKGDQIAKESQDLKEE
LVAAVEDVRKQGETMRIASSEFADDPCSSVKRGTMVRAARALLSAVTRLLILADMADVMRLLSHLKIVEEALEAVKNATNEQDLANRF
KEFGKEMVKLNYVAARRQQELKDPHCRDEMAAARGALKKNATMLYTASQAFLRHPDVAATRANRDYVFKQVQEAIAGISNAAQATSPT
DENKGHTGIGELAAALNEFDNKIILDPMTFSEARFRPSLEERLESIISGAALMADSSCTRDDRRKRIVAECKRAVRQALQDLLSEYMN
NTGRKEKGDPLNIAIDKMTKKTRDLRRQLRKAVMDHISDSFLETNVPLLVLIEAAKSGNEKEVKEYAQVFREHANKLVEVANLACSIS
NNEEGVKLVRMAATQIDSLCPQVINAALTLAARPQSKVAQDNMDVFKDQWEKQVRVLTEAVDDITSVDDFLSVSENHILEDVNKCVIA
LQEGDVDTLDRTAGAIRGRAARVIHIINAEMENYETGVYTEKVLEATKLLSETVMPRFAEQVEVAIEALSANVPQPFEENEFIDASRL
VYDGVRDIRKAVLMIRTPEELEDDSDFEQEDYDVRSRTSVQTEDDQLIAGQSARAIMAQLPQEEKAKIAEQVEIFHQEKSKLDAEVAK
WDDSGNDIIVLAKQMCMIMMEMTDFTRGKGPLKNTSDVINAAKKIAEAGSRMDKLARAVADQCPDSACKQDLLAYLQRIALYCHQLNI
CSKVKAEVQNLGGELIVSGLDSATSLIQAAKNLMNAVVLTVKASYVASTKYQKVYGTAAVNSPVVSWKMKAPEKKPLVKREKPEEYQT
RVRRGSQKKHISPVQALSEFKAMDSF

>XtCTNNA2
MSSATSPIILKWDPKSLEIRTLTVESLLEPLVTQVTTLVNTSNKGPSGKKKGRSKKAHVLAASVEQATQNFLEKGEQIAKESQDLKEE
LISAVEDVRKQGDTMRITSSEFADDPCSSVKRGTMVRAARALLSAVTRLLILADMADVMRLLIHLKIVEEALESVKNATNEQDLAHRF
KEFGKEMVKLNYVAARRQQELKDPHCRDEMAAARGALKKNATMLYTASQAFLRHPDVAATRANRDYVFKQVQEAIAGIANAAQATSPT
DEKQAHTGIGELAAALNEFDNKIILDPLTFSEARFRPSLEEKLESIISGAALMADSSCTRDDRRERIVAECNSVRQALQDLLSEYMNN
CRYGTWMDESCKSGRKEKGDPLNIAIDKMTKKTRDLRRQLRKAVMDHISDSFLETNVPLLVLIEAAKNGNEKEVKEYAQVFREHANKL
VEVANLACSISNNEEGVKLVRMAATQIDSLCPQVINAALTLAARPQSKVAQDNMDVFKDQWEKQVRVLTEAVDDITSVDDFLSVSENH
ILEDVNKCVIALQEGDVDTLDRTAGAIRGRAARVIHIINAEMENYEAGVYTEKVLDATKLLCETVMPRFAEQVEFAIEALSANIPQPF
EENEFIDASRLVYDGVRDIRKAVLMIRTPEELEDDSDFEQEDYDVRSRTSVQTEDDQLIAGQSARAIMAQLPQEEKAKIAEQVEIFHQ
EKSKLDAEVAKWDDSGNDIIVLAKQMCMIMMEMTDFTRGKGPLKNTSDVINAAKKIAEAGSRMDKLARAVADQCPDSACKQDLIAYLQ
RIALYCHQLNICSKVKAEVQNLGGELIVSGTAVQSTFTTFYEVAGDVIAGGRDSQLSLDLLPSCTEGSLFGSGSRDSTMLDSATSLIQ
AAKNLMNAVVLTVKASYVASTKYQKVYGTAAVNSPVVSWKMKAPEKKPLVKREKPEEYQTRVRRGSQKKHISPVQALSEFKAMDSF

>LcCTNNA2
MTSATSPILLKWDPKSLEIRTLTVERLLEPLVTQVTTLVNTSNKGPSSKKKGRSKKAHVLAASVEQATQNFLEKGEQIAKESLDLKEE
LVVAVEDVRKQGETMKVASSEFADDPCSSVKRGTMVRAARALLSAVTRLLILADMADVMRLLAHLKIVEEALEAVKNATNEQDLANRF
KEFGKEMVKLNYVAARRQQELKDPHSRDEMAAARGALKKNATMLYTASQAFLRHPDVAATRANRDYVFKQVQEAIAGISNAAQAASPT
DDKHGHTGIGELAAALNEFDNKIILDPLTFSEARFRPSLEERLESIISGAALMADSSCTRDDRRERIVAECNSVRQALQDLLTEYMNN
TGRKEKGDPLNSAIDKMTKKTRDLRRQLRKAVMDHISDSFLETNVPLLVLIEAAKSGNEKEVKEYAQVFREHANKLVEVANLACSISN
NEEGVKLVRMAATQIDSLCPQVINAALTLAARPQSKVAQDNMDVFKEQWEKQVRVLTEAVDDITSVDDFLSVSENHILEDVNKCVIAL
QEGDVDTLDRTAGAIRGRAARVIHIINAEMENYEAGVYTERVLEATKLLSETVMPRFAEQVEVAIEALSANVPQPFEENEFIDASRLV
YDGVRDIRKAVLMIRTPEELEDDSDFEQEDYDVRSRTSVQTEDDQLIAGQSARAIMAQLPQEEKAKIAEQVESFHQEKCKLDAEVAKW
DDSGNDIIVLAKQMCMIMMEMTDFTRGKGPLKNTSDVINAAKKIAEAGSRMDKLARAVADQCPDSACKQDLLAYLQRIALYCHQLNIC
SKVKAEVQNLGGELIVSGLDSATSLIQAAKNLMNAVVLTVKASYVASTKYQKVYGTAAVNSPVVSWKMKAPEKKPLVKREKPEDYQTR
VRRGSQKKHISPVQALSEFKAMDSF

>DrCTNNA2
MTSATSPILLKWDPKSLEIRTLTVERLLEPLVTQVTTLVNTSNKGPSSKKKGRSKKAHVLAVSVEQATQNFLEKGEQIAKDSQDLKEE
LIAAVEDVRKQGDTMRVASSEFADDPCSSVKRGTMVRAARALLSAVTRLLILADMADVMRLLAHLKIVEEALEAVKNATNEQDLANRF
KEFGKEMVKLNYVAARRQQELKDPQCRDEMAAARGALKKNATMLYTASQAFLRHPDVAATRANRDYVFKQVQEAIGGISSSAQATSPT
DEKHGHAGIGELAAALNEFDNKIILDPLTFSEARFRPSLEERLESIISGAALMADSSCTRDDRRERIVAECNAVRQALQDLLSEYMNN
TGRKEKGDPLNSAIDKMTKKTRDLRRQLRKAVMDHISDSFLETNVPLLVLIEAAKSGNEKEVKEYAQVFREHANKLVEVANLACSISN
NEEGVKLVRMAATQIDSLCPQVINAALTLAARPQSKVAQDNMDVFKDQWEKQVRILTEAVDDITSVDDFLSVSENHILEDVNKCVIAL
QEGDVDTLDRTAGAIRGRAARVVHIINAEMENYEPGVYTERVLESIKLLSETVMPRFAEQVEVAIEALSTSPPQPFEENEFIDASRLV
YDGVRDIRKAVLMIRTPEELEDDSDFEQEDYDARSRTSIQTEDDQLIAGQSARAIMAQLPQEEKAKIAEQVESFRQEKSKLDAEVAKW
DDNGNDIIVLAKQMCMIMMEMTDFTRGKGPLKNSSDVINAAKKIAEAGSRMDKLARAVADQCPDSACKQDLLAYLQRIALYCHQLNIC
SKVKAEVQNLGGELIVSGLDSATSLIQAAKNLMNAVVLTVKASYVASTKYQKVYGTAAVNSPVVSWRMKAPEKKPLVKREKPEECQTR
VRRGSQKKHISPVQALSEFKAMDSF

>LoCTNNA2
MTTATSPILLKWDPKSLEIRTLTVERLLEPLVTQVTTLVNTSNKGPSSKKKGRSKKAHVLAASVEQATQNFLEKGEQIAKESQDLKEE
LIAAVEDVRKQGETMRVASSEFADDPCSSVKRGTMVRAARALLSAVTRLLILADMADVMRLLAHLKIVEEALEAVKNATNEQDLANRF
KEFGKEMVKLNYVAARRQQELKDPHCRDEMAAARGALKKNATMLYTASQAFLRHPDVAATRANRDYVFKQVQEAIAGISSAAQATSPT
DEKHGHTGIGELAAALNEFDNKIILDPLTFSEARFRPSLEERLESIISGAALMADSSCTRDDRRERIVAECNAVRQALQDLLSEYMNN
TGRKEKGDPLNTAIDKMTKKTRDLRRQLRKAVMDHISDSFLETNVPLLVLIEAAKSGNEKEVKEYAQVFREHANKLVEVANLACSISN
NEEGVKLVRMAATQIDSLCPQVINAALTLAARPQSKVAQDNMDVFKDQWEKQVRVLTEAVDDITSVDDFLSVSENHILEDVNKCVIAL
QEGDVDTLDRTAGAIRGRAARVVHIINAEMENYEPGVYTERVLEAIKLLSETVMPRFAEQVEVAIEALSTNPPQPFEENEFIDASRLV
YDGVRDIRKAVLMIRTPEELEDDSDFEQEDYDARSRTSVQTEDDQLIAGQSARAIMAQLPQEEKAKIAEQVESFRQEKCKLDAEVAKW

DDNGNDIIVLAKQMCMIMMEMTDFTRGKGPLKNSSDVINAAKKIAEAGSRMDKLARAVADQCPDSACKQDLLAYLQRIALYCHQLNIC
SKVKAEVQNLGGELIVSGLDSATSLIQAAKNLMNAVVLTVKASYVASTKYQKVYGTAAVNSPVVSWRMKAPEKKPLVKREKPEECQTR
VRRGSQKKHISPVQALSEFKAMDSF

>CmCTNNA2
MTAATSPILLKWDPKSLEIRTVTVERLLEPLVTQVTTLVNTSSKGPSGKKKGRSKKAHVLAASVEQATQNFLEKGEQIAKESQDLKDE
LLAAVDDVRKQGETMRIASGDFADDPCSSVKRGTMVRAARALLSAVTRLLILADMADVMRLLTHLKIVEESLDAVKNATNEQDLANRF
KEFGKEMVKLNYVAARRQQELKDLHCRDEMAAARGALKKNATMLYTASQAFLRHPDVAATRANRDYVFKQVQEAIAGISNAAQSTSPL
DDKHGHPGIGELAAALNEFDNKIILDPMHFSEQRYRPSLEERLESIISGAALMADSSCTRDDRRERIVAECNAVRQALQDLLTEYMNN
LRKAVMDHISDSFLETNVPLLVLIEAAKSGNEKEVKEYAQVFREHANKLVEVANLACSISNNEEGVKLVRMAATQIDSLCPQENHILE
DVNKCVIALQEGDVDTLDRTAGAIRGRAARVIHIISAEMENYEPGVYTEKVMESTKLLSETGTPEELEDDSDFEQEDYDVRSRTSVQT
EDDQLIAGQSARRGKGPLKNTSDVINAAKKIAEAGSRMDKLARAVADQCPDSSCKQDLLAYLQRIALYCHQLNICSKVKAEVQNLGGE
LIVSG

>HsCTNNA3
MSAETPITLNIDPQDLQVQTFTVEKLLEPLIIQVTTLVNCPQNPSSRKKGRSKRASVLLASVEEATWNLLDKGEKIAQEATVLKDELT
ASLEEVRKESEALKVSAERFTDDPCFLPKREAVVQAARALLAAVTRLLILADMIDVMCLLQHVSAFQRTFESLKNVANKSDLQKTYQK
LGKELENLDYLAFKRQQDLKSPNQRDEIAGARASLKENSPLLHSICSACLEHSDVASLKASKDTVCEEIQNALNVISNASQGIQNMTT
PPEPQAATLGSALDELENLIVLNPLTVTEEEIRPSLEKRLEAIISGAALLADSSCTRDLHRERIIAECNAIRQALQDLLSEYMNNAGK
KERSNTLNIALDNMCKKTRDLRRQLRKAIIDHVSDSFLDTTVPLLVLIEAAKNGREKEIKEYAAIFHEHTSRLVEVANLACSMSTNED
GIKIVKIAANHLETLCPQIINAALALAARPKSQAVKNTMEMYKRTWENHIHVLTEAVDDITSIDDFLAVSESHILEDVNKCIIALRDQ
DADNLDRAAGAIRGRAARVAHIVTGEMDSYEPGAYTEGVMRNVNFLTSTVIPEFVTQVNVALEALSKSSLNVLDDNQFVDISKKIYDT
IHDIRCSVMMIRTPEELEDVSDLEEEHEVRSHTSIQTEGKTDRAKMTQLPEAEKEKIAEQVADFKKVKSKLDAEIEIWDDTSNDIIVL
AKNMCMIMMEMTDFTRGKGPLKHTTDVIYAAKMISESGSRMDVLARQIANQCPDPSCKQDLLAYLEQIKFYSHQLKICSQVKAEIQNL
GGELIMSALDSVTSLIQAAKNLMNAVVQTVKMSYIASTKIIRIQSPAGPRHPVVMWRMKAPAKKPLIKREKPEETCAAVRRGSAKKKI
HPLQVMSEFRGRQIY

>GgCTNNA3
MSTKVPNIKLKIDPQDLQIQTFTVEKLLEPLIIQVTTLVKCPQNPSGKKKGRSKRARILLASVEEATQNLLDKGEKIAKEAVVLKEEL
HAALADVQKESQALKISAEAFTGDPCYLPKRQAVVQTARSLLTAVTRLLILADMVDVAYLLEHLTVFQRTFETLRNVSSKSDLQNTYQ
KFQKDLENLDYLAYKRQQDLKSSDQRDEIAAARATLKENSSLLHSVCSACLEHSDVASLTASKDSICSEIQNALNVISNASQGVGNKK
GQPASYRATLGSALDELEHLIIQDPLVVSEEKIRPSLEKRLEAIISGAALLADSSCTRDFHRERIIAECNAIRQALQDLLSEYINNTD
RKERSDALNTAIDSMCKKTRDLRRQLRKAIIDHISDSFLDTTVPLLVLIEAAKNGREKEIKEYAAIFREHTGRLVEVANLACSLSTNE
DGMKIVQMAANHLETLCPQVINAALALSARPKSQVVKSNMEMYRSIWENHVHVLTEAVDDITSIDDFLAVSESHILEDVNKCIIALRE
QNADNLDRAAGAIRGRASRVAHIVSGEMDNYEPGAYTEGVMKNVQYLTKSVIPEFISQVNIALESLSKNTVHLFDDNQFVDVSKKVYD
TIHTIRCSVMMIRTPEELEDVSDLEDHDTRSRTSIQTEGKTDRAKMTQLPEAEKEKIAEQVADFKKVKSKLDAEIEIWDDTSNDIIVL
AKRMCVIMMEMTDFTRGRGPLKNTSDVINAAKMISESGSRMDVLARLIANQCPDQSCKQDLLAYLEQIKLYSHQLKICSQVRAEIQNL
GGELIVSALDSVTSLIQAAKNLMNAVVQTVKMSYIASTKIIKIQSPTGPRHPVVMWRMKAPEKKPLIKREKPEEIYAAVRKGSAKKRI
HPVQVMSEFRGREIV

>LcCTNNA3
MTSTLPKIQLKINPQDLQVRTFTVEKLLEPLIIQVTTLVNCPKNPSKKKKGCSKRARILLASVEEATWNLLDKGEKIAKDSKILKEEL
TAAVEDVRKESEILRLTAKDFTDDPCCLPKREAVVQAARALLAAVTRLLILADIIDVVYLLQHLTIVQKTFESLRNVSSRAELKKAYE
KFGKDLEALDHLAYKRQQDLKSIDQRDEIAGARASLKQNSTLLYSISTACLEHPDVASLKASKDSVCNGIQNALSVISDASQGIGHEK
TQLPLSSGTLGGALDEFQNWIILDPLTINEEKIRPTLEKRLEGIISGAALLADSSCTRDLHRERIIRECNAIRQALQDLLSEYMNNAG
MKERTNAVNDAIDNMCKRTRELRKQLRKAIIDHVSDSFLDTTVPLLVLIEAAKNGKEKEIKEYAAIFREHANKLVEVANLACSISTNT
DGIKIVQMAANHVETLCPQVINAALALAARPKSQVVKNNMEMYKKAWENHIRLLTEAVDDITRIDDFLAVSESHILEDVNKCIIALRE
RDLDSLDRAAGAIRGRAARVAHIVTGEMDNYESGVYTEGVMKNVQYLKEIAIPEFVSQVNIAIEVLSTDTMNQFDDNQFVDVSKKVYE
AIHDIRCSVLMIRTPEELEDVSDFEEDHETHSHTSGHTEGKTDRAKMTQLPDAEKAKIAEQVADFKKVKSKLDAEIEIWDDTSNDIIV
LAKKMCMIMMEMTDFTRGKGPLKNTSDVINAAKMISEAGSRMDKLARQIANLCPDPSCKQDLLAYLEQIKLYSHQLKICSQVKAEIQN
LGGELIMSALDSVTSLIQAAKNLMNAVVQTVKMSYIASSKIIRIHTTGPRHPVVMWRMKAPEKKPLIKREKPEEVYAAVRKGSAKKKI
HPVKAMSEFRGREVN

>LoCTNNA3
MTLLQNLQLKIDPKDLQIRTITVERLLEPLIIQVTTLVNCPKNPSNKKKGCSKRAKVLLKSVEEATLNLIDKGEMIAKETVVLKDELT
AAVKEVQRESHSLTWTAKEFTDDPCCLSKREAVVQAARTLLAAVTRLLILADIIDVIYLLEHLSTVQKTFEILRTSSNKIDLKKAYQQ
FGKELNVLDNLAHKRQQDLKSPDQRDEMASARGLLKQNSSLLYSTSSACLEHPDVASLKASKDSICNEIQDAFTAISNAAQGIGQAKV
QPMSTSGTLGGALDELESVIVIDPLTVNEDNIRPALEKRLEAIISGAALLADSSCTRDIHRERIITECNVVRQALQELLSEYIGNAGM
KERSNPLNNAIDNMCKRTRDLRRQLRKAIIDHISDSFLDTTVPLLVLIEAAKKGKEKEIKEYAAIFREHANKLMEVANLASSISTNDD
GVRIVQMAASRIETLCPQVINAALALASKPKSQAVKQNMEVYRQAWETHVRLLTEAVDDVTSIDDFLAVSESHILEDVNKCIIALREH
DFENLDRSAGAIRGRAARVAHVVTGEMDNYEPGVYTDGVMKNVNYLTENAIPEFVTQVSKAMDALNTGQMHQFDDNLFVDVSKKVYDS
IHDIRCSVLMIRTPEELEDLSDLEEEYESRSRTSVQTDGKTDRAKMTQLPESEKIKIAEQVADFKKVKSKLDAEIEIWDDTSNDIIVL
AKKMCMIMMEMTDFTRGKGPLKHTSDVINAAMVISEAGCRMDVLARQIANQCPDPSCKHDLLAYLEQIKLYSHQLKICSQVKSEIQSL
GGELITSALDSVTSLIQAAKNLMNAVVQTVKMSYIASTKIIRIHSPTGPRHPVVMWRMKAPEKKPLIKKEKPEEAYAVVRKGSTKKKI
HPVKVMSEFRGRQVQ

>CmCTNNA3
GHKESHSVNAAIDNMCKKTRVLRRQLRKAIIDHVSDSFLDTTVPLLVLIEAARSGKEKDIQEYGTIFREHTNKLIEVANLACSMSTNE
EGIKIVQIATNRIETMCPQVINAALALAAMPKSQTIKTNMEIYKKAWETHVQLLIEAVDDITCIDDFLAISESHILEDVNKCIIALRE
RDVDTLDRAAGAIHGRAARVSHVATAEMENYEPGVYTEGVMKSVQILAESAMPEFIAQVNIALEALSIDSPHQFDDNAFVDAAKKVYD
SVHDVRSAVLMIRTPEELEDVSDFEEDHDIRSRTSVQTEGKTDRAKMTELPEEEKAKIAEQVADFKKVKSKLDAEIEIWDDTSNDIIV
LAKKMCMIMMEMTDFTRGRGPLKHISDVINAAKTISESGSRMDILARQIANQCQDLSCKHDLLAYLEQIKLYSHQLKICSQVKAEIQS
LGGELIVSALDSITSLIHAAKNLMNAVVYTVKMSYIASTKIIRLQSPSGVHHSVVMWRMKAPEKKPLIKREKPEEMSAAVRKGSAKKK
IHPVKAMSEFRGRNVS

>PmCTNNA

MSSITSPNIQFKWDPKTLEIRTLSVEKTLEPLVQQVTTLVNINSKGPSNKKKGRSKKAHVLSASVEKATQNFIDKGDQIARESEYLKD
ELMDAVDQVRTQGETMRSAAGEFADDPCSSMKRGAMVRAARALLTAVTRLLILADMADVMKLLRHLRIVEETLEKVRMAGSQQELANT
FKKFGQEMVTLNHLAARRQQDLKDPRLRDEMAAARGSLKKNATMLYTASQAFLNHPDVAATKANRDYVYKQVQEAIASISNAAQATSV
PSRTQAADNVGELAAALNDFDTKIVLDPNTYNEARMRPSLEERLESIISGAALMADSSCTRDDRRERIVAECNSVRQALQDLLTEYMN
NAGRKEPTDVLNVAIDRMCQKTRDLRRQLRKAVMDHISDSFLETTVPLLVLIEAAKKGNEKEVKEYAQVFREHANKLVEVANLACSLS
SNEEGVKLVRMAAQQIETLCPQVINAALTLAAKPNSKIAQDNMDVFKETWENQVRLLTEAVDDITSIDDFLAVSENHILEDVNKCVMA
LQEGDVDALDRTAGAIRGRAARVVHVVNGEMENYEPGIYTEKVLESTTMLADSVMPRFAEQVENAIDALSSNPSRPVDENEFIDASRL
VYDGIRDIRKAVLMIRTPDELDDSDMEDETEVRSYTSVKTENDQIIEGSARALMAQLPQEEKAKIAEQVERFQEEKSKLDAEVAKWDD
SGNDIIVLSKQMCMIMMEMTDFTRGKGPLKNTSNVINAAKKIADLGSKMDKLARVIADQCPDSASKQDLLAYLQRIALYCHQLNICSK
VKAEVQNISGEVVVSGLDSATSLIQAAKNLMNAVVLTVKASYVASSKFHKVQHTNINSPVVSWKMKAPEKKPLVKRERGDDVSSHVRR
GSQKKHISPVQALSEFKGRETY

>BfCTNNA
MASPGGTLKWDPKNLEIKTHSVEKTLEPLVTQVTTLVNTKGNSGKKKGRSKKAHVLSALVKKATETFIDKGEIIAKDYPDIQDDMMAA
IDDCKKTGDTMYAASQEFADDPCSSMKRGAMVRAARALLSAVTRLLIIADMADVHMLLKHLRIVEESLERVRQAQDQQALNNAFRQYG
KDVVGLNDIAMKRQNDLIDPARRDEMAAARNTLKKNSMMLYTASKAFLRHPDVAAAKANRDYVYKQVCDAVNSLSNTAQASGPADPHP
YEGAGELAAALDEFDQKIVMDPASYNEVRTRPSLEERLESIISGAALMADSSCTRDDRRERIVAECNAVRQALQDLLTEYMNCAGGRK
TKELDEAVDRMHKKTRDLRRQLRKAVVDHVSDSFLETNVPLLVLIEAAKRGNEKEVKEYAQVFREHANKLVEVANLACSMSNNEEGVK
LVRLAANQIENLCPQVINAALTLAARPRSKVAQENMDVFRDQWETQVRLLTEAVDDITSIDDFLAVSEQHILEDVNKCLLARQENDAD
TFDRTAGAIRGRVSRVCNVVNAEMDNYEPGIYTERVREAVVLLGDQVMPAFTECVEIVVDALSQDPPKETDENELIDSSRNVYEGVRD
VRRAVLMIRSPEELETDTEYEDETYETHSRSSRMTDDRELEGSQAGEFGGKSARALMRELPEEEKKKIAHQLEVFRAEKSKLDEEIAK
WDDSGNDIIVLAKQMCMIMMEMTDFTRGHGPLKSTMDVINAAKRIAEAGTKMDKLARAIADQCPESQTKDDLLAYLQRIALFCHQLNI
ASKVKAEVQNISGELIVSGPHWASMLDSATSLITASKNLMNAVVLTVKSCYVASTKYKKAMKNHNVNSPVVLWKMKAPEKKPLVRREK
PEELSAKVRKGSQKRHFSPIQALSEFSSRDTF

>CiCTNNA
MSSVASSNSGQKWNPKNLEIRTLSVEKTLEPLVVQVTTLVNNKGPSNKKKGRSKRPKVLVADVEKATSLFIEHGLEIARDNPELKAEL
EEAVEDTRRDGEVMSKTSHAFADDPCSSMKRGSMVRAARALLSSVTRLLILADMADIYNLLRQLKIARKSVGLVKDSNNPDDLIKNFK
AFGTDLARLNHATMLRQNDLKDLRRRDELAAARADLKKNSMMLYTSSLASIRHPEVAAAKANRDYVYRKVLGAMDRISTAVQATGPSE
PRPEEGAGELAFALDEFLQRVDMEPLDFDEVRHRPSLEERLESIISGAALMADSSCTREDRRERIVAECNAVRQALQDLLQEYLNNAG
RRDKSDDLEAAVDHMMQKTKDLRRQLCKAVIDHVSDSFLDTNIPILVLIEAAQSGDEKQVKEYAQVFREHANKLVEVANLACSMSSNE
EGVKMVKMAAKQIETLCPQVINAALTLAARPSSKIAQDNMNVFKDAWLNQVQTLTEAVDEITTIENFLAVSEAHILEDVTSCVVALQQ
GNPEMLDRKGGDIRGRTARICQVVISEMEHYEPDYYTQKVVDSCEMLKSQVMPKFEARLETTINALAKDPQEDPQENEFIDSSRFVYD
AVREVRRAVLMIKNPEDVDDSDIDAMDGSESGASRLSGKTVETESGPTDQTMMRRLPEAEKAKILQNVQEFVKEKTLLDLEISKWDES
SNDIIVLAKSMCVIMMDMTDFTRGRGRLKNTSDVIDAAREIADAGSRLDKLARAIAEVCPDSSVKQDLLAYLQRIALYCHQLNMCAKV
KAEVQNISGEMIVSGLDSATSLIQAAKNLMNAVVLTVKSSYVGSTKYRKAGTMHTDGILWKMKAPEKKPLIRREIGDDKKAPVRRASQ
KRLVNPAQALSEFKADISF

>DmCTNNA
MLKPDKMGTLTDFGQIALKWDPKNLEIRTMSVEKTLEPLVLQVTTLVNTKGPSKKKKGKSKRASALVAAVEKATENFIQKGEQIAYEN
PDITQEMLTAVDEVKKTGDAMSIAAREFSEDPCSSLKRGNMVRAARNLLSAVTRLLILADMVDVHLLLKSLHIVEDDLNKLKNASSQD
ELMDNMRQFGRNAGELIKQAAKRQQELKDPQLRDDLAAARAMLKKHSTMLLTASKVYVRHPELDLAKVNRDFILKQVCDAVNTISDVA
QGKSSQPTDIYSGAGELAAALDDFDEGIVMDPMTYSEKRSRQLLEERLESIISAAALMADADCTRDERRERIVAECNAVRQALQDLLS
EYMSNMSQKDNSPGLSRAIDQMCRKTRDLRRQLRKAVVDHVSDSFLETTTPLLDLIEAAKSGNEKKVREKSEIFTKHAEKLVEVANLV
CSMSNNEDGVKMVRYAAAQIESLCPQVINAASILTVRPNSKVAQENMTTYRQAWEVQVRILTEAVDDITTIDDFLAVSENHILEDVNK
CVMALQVGDARDLRATAGAIQGRSSRVCNVVEAEMDNYEPCIYTKRVLEAVKVLRDQVMMKFDQRVGAAVGALSNNSNKDVDENDFID
ASRLVYDGVREIRRAVLMNRSSEDLDTDTEFEPVEDLTLETRSRSSAHTGDQTVDEYPDISGICTAREAMRKMTEEDKQKIAQQVELF
RREKLTFDSEVAKWDDTGNDIIFLAKHMCMIMMEMTDFTRGRGPLKTTMDVINAAKKISEAGTKLDKLTREIAEQCPESSTKKDLLAY
LQRIALYCHQIQITSKVKADVQNISGELIVSGLDSATSLIQAAKNLMNAVVLTVKYSYVASTKYTRQGTVSSPIVVWKMKAPEKKPLV
RPEKPEEVRAKVRKGSQKKVQNPIHALSEFQSPADAV
