# Supplementary material for: Origin and Loss of Nested LRRTM/α-Catenin Genes during Vertebrate Evolution
Source: PLoS One. 2014 Feb 24;9(2):e89910. doi: 10.1371/journal.pone.0089910 (PMC3933685; doi:10.1371/journal.pone.0089910)
Supplement: Table S3 — Conservation of selected human different strand nested gene structures in vertebrate genomes. (PDF) [file pone.0089910.s010.pdf]

Table S3. Conservation of selected different strand nested gene structures in vertebrates

The table lists 91 different strand nested (DN) gene structures that were selected from *Ho MR, Tsai KW, Lin WC. (2012) A unified framework of overlapping genes: Towards the origination and endogenic regulation. Genomics 100: 231-239.* We included in the list only (i) clear protein-coding nested gene pairs that are conserved in human and mouse (ii) in which the nested gene is flanked by protein coding exons of the host gene. So we excluded those nested gene structures in which either the host or the nested gene is a RNA gene, pseudogene or a predicted ORF, or if the flanking exon of the host gene is non-coding or first signal sequence exon.

Simple analysis of conservation:
young = nested gene structure is absent (not annotated) in coelacanth and zebrafish genomes (Ensembl v72).
conserved = nested gene structure is present (annotated) in coelacanth and/or zebrafish AND in both *X. tropicalis* and chicken genomes.
not conserved = nested gene structure is present in coelacanth and/or zebrafish genomes BUT is missing or disrupted in either chicken or *X. tropicalis* genomes.
LRR-domain containing genes are underlined.

**Host gene**	**Nested gene**	Conservation	Explanation
ACAD11	*CCRL1*	conserved	
ACSF2	*CHAD*	conserved	
ANO3	*MUC15*	young	
ARHGAP6	*AMELX*	young	
ARL13B	*STX19*	conserved	
ART3	*CXCL10*	young	
ART3	*CXCL11*	young	
ASTN2	***TRIM32***	not conserved	nested gene absent in *X. tropicalis*
BET3L	*FAM26E*	conserved	
BRF1	*BTBD6*	conserved	
CEP85L	*PLN*	young	
CACNA2D3	***LRTM1***	not conserved	both genes absent in *X. tropicalis*
CACNA2D4	*LRTM2*	conserved	
CASK	*GPR34*	conserved	
CASK	***GPR82***	not conserved	nested gene absent in *X. tropicalis*
CCDC146	*FGL2*	conserved	
CDC20B	*GPX8*	conserved	
CDC73	*B3GALT2*	conserved	
CENPP	*ECM2*	not conserved	host and OMD absent in *X. tropicalis*
	ASPN	not conserved	
	OMD	not conserved	
	OGN	not conserved	
COG5	*GPR22*	conserved	
CORO7	*VASN*	conserved	
CSMD2	*HMGB4*	young	
CTNNA1	***LRRTM2***	not conserved	nested gene absent in *X. tropicalis*
CTNNA2	*LRRTM1*	conserved	
CTNNA3	*LRRTM3*	not conserved	host gene absent in *X. tropicalis*
CTPS2	*S100G*	young	
DOCK1	*FAM196A*	conserved	
DOCK2	*FAM196B*	conserved	
DOCK7	*ANGPTL3*	conserved	
ECE2	*CAMK2N2*	conserved	
FAM83E	*SPACA4*	young	
FBXL13	***LRRC17***	not conserved	nested gene absent in *X. tropicalis*
FYCO1	***CXCR6***	not conserved	host gene absent in *X. tropicalis*,<br>nested gene absent in chicken
GFM1	*LXN*	conserved	
GIGYF2	*KCNJ13*	conserved	

GLRA4	*TMEM31*	young	
GTF2F2	*KCTD4*	conserved	
HERC3	*NAP1L5*	young	
IFT140	*TMEM204*	conserved	
IFT74	*LRRC19*	conserved	
IL1RAPL2	*TEX13A*	young	
IMMP2L	*LRRN3*	not conserved	host gene absent in *X. tropicalis*
IQGAP2	*F2RL2*	conserved	
ITGAE	*GSG2*	young	
LIMS2	*GPR17*	conserved	
LRBA	*MAB21L2*	conserved	
MACROD1	*FLRT1*	young	
MACROD2	*FLRT3*	conserved	
MCM9	*ASF1A*	young	
MCPH1	*ANGPT2*	conserved	
	GPR171	young	host gene absent in *X. tropicalis*
	P2RY14	young	
MED12L	*GPR87*	young	
	P2RY13	not conserved	
	P2RY12	not conserved	
METTL9	*IGSF6*	young	
MTOR	*ANGPTL7*	conserved	
NBEA	*MAB21L1*	conserved	
NF1	*EVI2A*	conserved	
NF1	*EVI2B*	conserved	
NF1	*OMG*	conserved	
NT5DC1	*COL10A1*	young	
PC	***LRFN4***	not conserved	both genes absent in chicken
PDGFD	*DDI1*	young	
PI4KA	*SERPIND1*	conserved	
PNKD	*TMBIM1*	young	
PRKG1	*CSTF2T*	young	
PSMD1	*HTR2B*	conserved	
RAB37	*CD300LF*	young	
RALGPS1	*ANGPTL2*	conserved	
RALGPS2	*ANGPTL1*	conserved	
RB1	*LPAR6*	conserved	
RNF123	***AMIGO3***	not conserved	nested gene absent in *X. tropicalis*
RTDR1	*GNAZ*	conserved	
SHC4	*EID1*	young	
SLC5A10	*FAM83G*	conserved	
SND1	*LRRC4*	not conserved	host gene absent in chicken
SORCS2	*PSAPL1*	young	
SYN1	***TIMP1***	not conserved	nested gene absent in *X. tropicalis*
SYN3	*TIMP3*	conserved	
TBCD	*ZNF750*	conserved	
TFB1M	***CLDN20***	not conserved	nested gene absent in *X. tropicalis*
TG	*SLA*	young	
UBAC2	*GPR183*	conserved	
	GPR18	conserved	
VEPH1	*PTX3*	conserved	
WDFY4	*LRRC18*	conserved	
XPNPEP3	*DNAJB7*	young	
