# Supplementary material for: Origin and Loss of Nested LRRTM/α-Catenin Genes during Vertebrate Evolution
Source: PLoS One. 2014 Feb 24;9(2):e89910. doi: 10.1371/journal.pone.0089910 (PMC3933685; doi:10.1371/journal.pone.0089910)
Supplement: Table S4 — BLAST analysis of tunicate and lancelet genomes using lamprey LRRTM sequences as query and reciprocal BLAST analysis against vertebrate genomes. (PDF) [file pone.0089910.s011.pdf]

Table S4. BLASTP analysis of tunicate and lancelet genomes using lamprey LRRTM sequences as query and reciprocal BLASTP analysis against all vertebrate genomes

Query sequence	Best BLASTP hit [*species*]	Accession number	E-value	Best reciprocal BLASTP hit* [*species*]
LRRTM_A	slit homolog protein 2 [*Ciona intestinalis*]	XP_002124095	3e-29	slit homolog 2 [*Melopsittacus undulatus*]
	hypothetical protein ID: 92046 [*Branchiostoma floridae*]	XP_002606179	3e-44	slit homolog 3 [*Sarcophilus harrisii*]
LRRTM_B	vasorin-like [*Ciona intestinalis*]	XP_002128185	2e-29	vasorin precursor [*Xenopus (Silurana) tropicalis*]
	hypothetical protein ID: 88462 [*Branchiostoma floridae*]	XP_002611139	4e-46	leucine-rich repeat-containing protein 15-like [*Maylandia zebra*]

*Reciprocal BLASTP was run against vertebrates (taxid:7742) with default parameters except that low complexity filter was on and compositional adjustments option was off.
