# Supplementary material for: Origin and Loss of Nested LRRTM/α-Catenin Genes during Vertebrate Evolution
Source: PLoS One. 2014 Feb 24;9(2):e89910. doi: 10.1371/journal.pone.0089910 (PMC3933685; doi:10.1371/journal.pone.0089910)
Supplement: Table S5 — Amino acid sequence identity (%) between vertebrate LRRTMs. (PDF) [file pone.0089910.s012.pdf]

Table S5. Amino acid sequence identity (%) between vertebrate LRRTMs.

	LRRTM1							LRRTM2						LRRTM3						LRRTM4										Lamprey	
	Hs	Gg	Xt	Lc	Dr	Lo	Cm	Hs	Gg	Lc	Dr	Lo	Cm	Hs	Gg	Xt	Lc	Lo	Cm	Hs	Gg	Xt	Lc	Dr_1	Dr_2	Dr_3	Dr_4	Lo	Cm	A	B
HsLRRTM1		82	78	76	65	73	67	47	46	46	49	47	48	46	46	45	46	46	37	46	47	46	48	43	45	42	42	46	38	45	42
GgLRRTM1	82		83	80	70	78	69	48	47	48	49	48	50	46	46	46	47	47	38	45	46	46	47	43	44	41	41	45	39	46	42
XtLRRTM1	78	83		79	69	77	68	47	47	48	48	46	48	44	45	44	45	45	36	45	45	46	46	43	44	41	40	44	37	43	42
LcLRRTM1	76	80	79		71	79	71	48	47	48	50	47	48	46	46	45	47	46	39	45	46	46	47	44	44	42	40	45	39	44	43
DrLRRTM1	65	70	69	71		80	66	48	47	48	49	46	47	44	45	43	45	45	38	45	45	45	45	41	42	40	39	43	38	43	41
LoLRRTM1	73	78	77	79	80		72	50	49	50	51	48	50	47	47	46	48	47	38	48	48	48	49	45	46	42	42	47	39	46	42
CmLRRTM1	67	69	68	71	66	72		54	53	52	55	49	51	48	48	45	49	48	42	44	44	46	47	42	41	40	41	43	40	41	39
HsLRRTM2	47	48	47	48	48	50	54		89	76	70	65	69	44	44	42	44	44	30	42	41	42	42	41	38	41	38	41	29	42	40
GgLRRTM2	46	47	47	47	47	49	53	89		71	67	64	70	43	43	41	43	43	30	42	41	41	42	42	39	40	38	41	28	42	38
LcLRRTM2	46	48	48	48	48	50	52	76	71		74	67	71	44	44	42	44	43	30	43	43	44	44	41	39	42	39	41	28	42	41
DrLRRTM2	49	49	48	50	49	51	55	70	67	74		67	72	45	44	41	44	44	34	44	43	44	45	40	38	39	39	43	34	39	39
LoLRRTM2	47	48	46	47	46	48	49	65	64	67	67		84	42	42	41	42	42	29	41	42	42	41	40	38	39	38	40	26	39	38
CmLRRTM2	48	50	48	48	47	50	51	69	70	71	72	84		44	44	42	43	44	30	42	43	43	43	41	39	41	39	42	30	42	40
HsLRRTM3	46	46	44	46	44	47	48	44	43	44	45	42	44		97	76	89	85	74	62	63	62	64	56	53	53	52	60	53	56	53
GgLRRTM3	46	46	45	46	45	47	48	44	43	44	44	42	44	97		76	89	85	72	63	63	62	64	57	53	54	52	61	54	55	53
XtLRRTM3	45	46	44	45	43	46	45	42	41	42	41	41	42	76	76		71	70	54	54	55	54	56	50	49	51	49	53	46	50	47
LcLRRTM3	46	47	45	47	45	48	49	44	43	44	44	42	43	89	89	71		87	74	62	63	61	64	57	54	53	51	61	53	54	53
LoLRRTM3	46	47	45	46	45	47	48	44	43	43	44	42	44	85	85	70	87		70	61	61	60	63	56	53	54	52	60	53	54	53
CmLRRTM3	37	38	36	39	38	38	42	30	30	30	34	29	30	74	72	54	74	70		57	59	59	62	56	49	45	43	57	57	50	38
HsLRRTM4	46	45	45	45	45	48	44	42	42	43	44	41	42	62	63	54	62	61	57		90	83	84	68	62	57	55	73	57	53	50
GgLRRTM4	47	46	45	46	45	48	44	41	41	43	43	42	43	63	63	55	63	61	59	90		82	86	68	63	56	55	74	56	54	50
XtLRRTM4	46	46	46	46	45	48	46	42	41	44	44	42	43	62	62	54	61	60	59	83	82		80	68	63	57	56	72	56	55	51
LcLRRTM4	48	47	46	47	45	49	47	42	42	44	45	41	43	64	64	56	64	63	62	84	86	80		71	64	58	57	74	59	57	52
DrLRRTM4_1	43	43	43	44	41	45	42	41	42	41	40	40	41	56	57	50	57	56	56	68	68	68	71		71	61	60	80	54	54	49
DrLRRTM4_2	45	44	44	44	42	46	41	38	39	39	38	38	39	53	53	49	54	53	49	62	63	63	64	71		58	58	74	50	50	47
DrLRRTM4_3	42	41	41	42	40	42	40	41	40	42	39	39	41	53	54	51	53	54	45	57	56	57	58	61	58		65	63	45	50	45
DrLRRTM4_4	42	41	40	40	39	42	41	38	38	39	39	38	39	52	52	49	51	52	43	55	55	56	57	60	58	65		63	43	48	44
LoLRRTM4	46	45	44	45	43	47	43	41	41	41	43	40	42	60	61	53	61	60	57	73	74	72	74	80	74	63	63		56	54	51
CmLRRTM4	38	39	37	39	38	39	40	29	28	28	34	26	30	53	54	46	53	53	57	57	56	56	59	54	50	45	43	56		44	40
PmLRRTM_A	45	46	43	44	43	46	41	42	42	42	39	39	42	56	55	50	54	54	50	53	54	55	57	54	50	50	48	54	44		47
PmLRRTM_B	42	42	42	43	41	42	39	40	38	41	39	38	40	53	53	47	53	53	38	50	50	51	52	49	47	45	44	51	40	47	

The sequence identity values were calculated from the alignment shown in Fig. S1. Cm = *Callorhinchus milii* (elephant shark), Dr = *Danio rerio* (zebrafish), Gg = *Gallus gallus* (chicken), Hs = *Homo sapiens*, Lc = *Latimeria chalumnae* (coelacanth), Lo = *Lepisosteus oculatus* (spotted gar), Pm = *Petromyzon marinus* (sea lamprey), Xt = *Xenopus tropicalis* (African clawed frog). Note that values corresponding to the shark orthologs are based on partial sequence data.
