# Supplementary material for: Origin and Loss of Nested LRRTM/α-Catenin Genes during Vertebrate Evolution
Source: PLoS One. 2014 Feb 24;9(2):e89910. doi: 10.1371/journal.pone.0089910 (PMC3933685; doi:10.1371/journal.pone.0089910)
Supplement: Table S6 — Alternative splicing of neurexin AS4 exon in selected species. (PDF) [file pone.0089910.s013.pdf]

Table S6. Neurexin AS4 dbEST TBLASTN search results, alignment score table and list of validated hits for human, mouse, *Xenopus*, zebrafish, sea lamprey and *Ciona* ESTs.
In parentheses are given the number of AS4 splice variants *vs.* number of hits fulfilling the search criteria and containing at least part of this sequence area. The AS4 exon sequence is highlighted in yellow. The aligments were generated using a 160 residue mouse neurexin-1 fragment as a query (described in Fig. S5) using BLOSUM62 matrix and default Entrez BLAST settings.

HUMAN (9/23 splice variants)

```
Sequences producing significant alignments:                       (Bits)  Value

emb|BX431035.2|  BX431035 Homo sapiens FETAL BRAIN Homo sapien...    216   8e-70
gb|CN262436.1|  17000600092091 GRN_PRENEU Homo sapiens cDNA 5'...    206   2e-66
dbj|DB171492.1|  DB171492 TKIDN2 Homo sapiens cDNA clone TKIDN...    198   3e-62
dbj|DB488484.1|  DB488484 RIKEN full-length enriched human cDN...    196   6e-62
dbj|DB218625.1|  DB218625 TRACH3 Homo sapiens cDNA clone TRACH...    183   3e-56
dbj|DB462876.1|  DB462876 RIKEN full-length enriched human cDN...    178   1e-54
emb|BX431034.2|  BX431034 Homo sapiens FETAL BRAIN Homo sapien...    177   2e-54
dbj|HY001490.1|  HY001490 RIKEN full-length enriched human cDN...    177   4e-54
dbj|DB476019.1|  DB476019 RIKEN full-length enriched human cDN...    172   2e-52
gb|CB154877.1|  K-EST0212952 B2N807043 Homo sapiens cDNA clone...    166   7e-50
gb|BF345183.1|  602018394F1 NCI_CGAP_Brn67 Homo sapiens cDNA c...    161   1e-46
dbj|DA747306.1|  DA747306 NT2RP7 Homo sapiens cDNA clone NT2RP...    157   5e-46
dbj|HY037592.1|  HY037592 RIKEN full-length enriched human cDN...    144   2e-41
dbj|DB465352.1|  DB465352 RIKEN full-length enriched human cDN...    139   8e-40
gb|BF529228.1|  602041680F1 NCI_CGAP_Brn67 Homo sapiens cDNA c...  94.7    1e-37
dbj|HY044110.1|  HY044110 RIKEN full-length enriched human cDN...    134   2e-37
gb|BQ723058.1|  AGENCOURT_8109177 Lupski_sympathetic_trunk Hom...    127   2e-33
gb|BG913108.1|  602811713F1 NCI_CGAP_Brn67 Homo sapiens cDNA c...    120   9e-31
dbj|HY135994.1|  HY135994 RIKEN full-length enriched human cDN...    116   2e-30
dbj|DA690044.1|  DA690044 NT2NE2 Homo sapiens cDNA clone NT2NE...    105   3e-26
gb|AW894889.1|  CM1-NN0033-110400-181-e04 NN0033 Homo sapiens ...    100   2e-24
gb|BQ879316.1|  AGENCOURT_8113386 Lupski_dorsal_root_ganglion ...  99.8    3e-23
dbj|DA745012.1|  DA745012 NT2RP7 Homo sapiens cDNA clone NT2RP...  94.4    8e-22
gb|AA984057.1|  am76b03.s1 Stratagene schizo brain S11 Homo sa...  63.9    4e-21
gb|CB991846.1|  AGENCOURT_13618102 NIH_MGC_148 Homo sapiens cD...  92.8    7e-21
gb|CB994026.1|  AGENCOURT_13617369 NIH_MGC_148 Homo sapiens cD...  93.2    8e-21
gb|EL737097.1|  14205 Full Length cDNA from the Mammalian Gene...  91.7    9e-21
gb|CB994400.1|  AGENCOURT_13642788 NIH_MGC_148 Homo sapiens cD...  92.4    1e-20
gb|BM724595.1|  UI-E-EO1-aiz-l-10-0-UI.r1 UI-E-EO1 Homo sapien...  88.6    4e-20
gb|BG820026.1|  602782573F1 NCI_CGAP_Brn67 Homo sapiens cDNA c...  88.2    6e-20
gb|BM801146.1|  AGENCOURT_6422532 NIH_MGC_92 Homo sapiens cDNA...  85.5    6e-18
dbj|DB464606.2|  DB464606 RIKEN full-length enriched human cDN...  82.4    3e-17
gb|BF346913.1|  602021742F1 NCI_CGAP_Brn67 Homo sapiens cDNA c...  71.2    9e-14
```

```
ALIGNMENTS
>emb|BX431035.2| BX431035 Homo sapiens FETAL BRAIN Homo sapiens cDNA clone
CS0DF015YG21 5-PRIME, mRNA sequence. Length=451

 Score =   216 bits (551),  Expect = 8e-70, Method: Compositional matrix adjust.
 Identities = 114/148 (77%), Positives = 117/148 (79%), Gaps = 30/148 (20%)
 Frame = +1

Query  1    QGKIGVKFNVGTDDIAIEESNAIINDGKYHVVRFTRSGGNATLQVDSWPVIERYPAGNND  60
            QGKIGVKFNVGTDDIAIEESNAIINDGKYHVVRFTRSGGNATLQVDSWPVIERYPA
Sbjct  4    QGKIGVKFNVGTDDIAIEESNAIINDGKYHVVRFTRSGGNATLQVDSWPVIERYPA----  171

Query  61   NERLAIARQRIPYRLGRVVDEWLLDKGRQLTIFNSQATIIIGGKEQGQPFQGQLSGLYYN  120
                                      GRQLTIFNSQATIIIGGKEQGQPFQGQLSGLYYN
Sbjct  172  --------------------------GRQLTIFNSQATIIIGGKEQGQPFQGQLSGLYYN  273
```

```
Query  121  GLKVLNMAAENDANIAIVGNVRLVGEVP  148
            GL+ L+MAAENDA+IAIVGNVRLVGEVP
Sbjct  274  GLESLDMAAENDAHIAIVGNVRLVGEVP  357
```

>gb|CN262436.1| 17000600092091 GRN_PRENEU Homo sapiens cDNA 5', mRNA sequence. Length=307

```
 Score =   206 bits (525),  Expect = 2e-66, Method: Compositional matrix adjust.
 Identities = 101/101 (100%), Positives = 101/101 (100%), Gaps = 0/101 (0%)
 Frame = +1

Query  1    QGKIGVKFNVGTDDIAIEESNAIINDGKYHVVRFTRSGGNATLQVDSWPVIERYPAGNND  60
            QGKIGVKFNVGTDDIAIEESNAIINDGKYHVVRFTRSGGNATLQVDSWPVIERYPAGNND
Sbjct  4    QGKIGVKFNVGTDDIAIEESNAIINDGKYHVVRFTRSGGNATLQVDSWPVIERYPAGNND  183

Query  61   NERLAIARQRIPYRLGRVVDEWLLDKGRQLTIFNSQATIII  101
            NERLAIARQRIPYRLGRVVDEWLLDKGRQLTIFNSQATIII
Sbjct  184  NERLAIARQRIPYRLGRVVDEWLLDKGRQLTIFNSQATIII  306
```

>dbj|DB171492.1| DB171492 TKIDN2 Homo sapiens cDNA clone TKIDN2006442 5', mRNA sequence. Length=569

```
 Score =   198 bits (504),  Expect = 3e-62, Method: Compositional matrix adjust.
 Identities = 92/131 (70%), Positives = 110/131 (84%), Gaps = 0/131 (0%)
 Frame = +2

Query  18   EESNAIINDGKYHVVRFTRSGGNATLQVDSWPVIERYPAGNNDNERLAIARQRIPYRLGR  77
            +E    +NDGKYHVVRFTR+GGNATLQVD+WPV E YP GN DNER  + +Q+IP++  R
Sbjct  2    KEERTPVNDGKYHVVRFTRNGGNATLQVDNWPVNEHYPTGNTDNERFQMVKQKIPFKYNR  181

Query  78   VVDEWLLDKGRQLTIFNSQATIIIGGKEQGQPFQGQLSGLYYNGLKVLNMAAENDANIAI  137
             V+EWL +KGRQLTIFN+QA I IGGK++G+ FQGQLSGLYY+GLKVLNMAAEN+ NI I
Sbjct  182  PVEEWLQEKGRQLTIFNTQAQIAIGGKDKGRLFQGQLSGLYYDGLKVLNMAAENNPNIKI  361

Query  138  VGNVRLVGEVP  148
             G+VRLVGEVP
Sbjct  362  NGSVRLVGEVP  394
```

>dbj|DB488484.1| DB488484 RIKEN full-length enriched human cDNA library, hypothalamus Homo sapiens cDNA clone H033022N07 5', mRNA sequence. Length=453

```
 Score =   196 bits (499),  Expect = 6e-62, Method: Compositional matrix adjust.
 Identities = 94/94 (100%), Positives = 94/94 (100%), Gaps = 0/94 (0%)
 Frame = +1

Query  54   YPAGNNDNERLAIARQRIPYRLGRVVDEWLLDKGRQLTIFNSQATIIIGGKEQGQPFQGQ  113
            YPAGNNDNERLAIARQRIPYRLGRVVDEWLLDKGRQLTIFNSQATIIIGGKEQGQPFQGQ
Sbjct  1    YPAGNNDNERLAIARQRIPYRLGRVVDEWLLDKGRQLTIFNSQATIIIGGKEQGQPFQGQ  180

Query  114  LSGLYYNGLKVLNMAAENDANIAIVGNVRLVGEV  147
            LSGLYYNGLKVLNMAAENDANIAIVGNVRLVGEV
Sbjct  181  LSGLYYNGLKVLNMAAENDANIAIVGNVRLVGEV  282
```

>dbj|DB218625.1| DB218625 TRACH3 Homo sapiens cDNA clone TRACH3008421 5', mRNA sequence. Length=560

```
 Score =   183 bits (465),  Expect = 3e-56, Method: Compositional matrix adjust.
 Identities = 95/125 (76%), Positives = 95/125 (76%), Gaps = 30/125 (24%)
```

```
 Frame = +1

Query  24    INDGKYHVVRFTRSGGNATLQVDSWPVIERYPAGNNDNERLAIARQRIPYRLGRVVDEWL  83
             INDGKYHVVRFTRSGGNATLQVDSWPVIERYPAG
Sbjct  1     INDGKYHVVRFTRSGGNATLQVDSWPVIERYPAG--------------------------  102

Query  84    LDKGRQLTIFNSQATIIIGGKEQGQPFQGQLSGLYYNGLKVLNMAAENDANIAIVGNVRL  143
                 RQLTIFNSQATIIIGGKEQGQPFQGQLSGLYYNGLKVLNMAAENDANIAIVGNVRL
Sbjct  103   ----RQLTIFNSQATIIIGGKEQGQPFQGQLSGLYYNGLKVLNMAAENDANIAIVGNVRL  270

Query  144   VGEVP  148
             VGEVP
Sbjct  271   VGEVP  285


>dbj|DB462876.1| DB462876 RIKEN full-length enriched human cDNA library, hippocampus
Homo sapiens cDNA clone H023002C01 5', mRNA sequence. Length=480

 Score =   178 bits (451),  Expect = 1e-54, Method: Compositional matrix adjust.
 Identities = 85/85 (100%), Positives = 85/85 (100%), Gaps = 0/85 (0%)
 Frame = +3

Query  64    LAIARQRIPYRLGRVVDEWLLDKGRQLTIFNSQATIIIGGKEQGQPFQGQLSGLYYNGLK  123
             LAIARQRIPYRLGRVVDEWLLDKGRQLTIFNSQATIIIGGKEQGQPFQGQLSGLYYNGLK
Sbjct  3     LAIARQRIPYRLGRVVDEWLLDKGRQLTIFNSQATIIIGGKEQGQPFQGQLSGLYYNGLK  182

Query  124   VLNMAAENDANIAIVGNVRLVGEVP  148
             VLNMAAENDANIAIVGNVRLVGEVP
Sbjct  183   VLNMAAENDANIAIVGNVRLVGEVP  257


>emb|BX431034.2| BX431034 Homo sapiens FETAL BRAIN Homo sapiens cDNA clone
CS0DF015YG21 5-PRIME, mRNA sequence. Length=420

 Score =   177 bits (448),  Expect = 2e-54, Method: Compositional matrix adjust.
 Identities = 93/129 (72%), Positives = 97/129 (75%), Gaps = 30/129 (23%)
 Frame = +1

Query  1     QGKIGVKFNVGTDDIAIEESNAIINDGKYHVVRFTRSGGNATLQVDSWPVIERYPAGNND  60
             QGKIGVKFNVGTDDIAIEESNAIINDGKYHVVRFTRSGGNATLQVDSWPVIERYPA
Sbjct  4     QGKIGVKFNVGTDDIAIEESNAIINDGKYHVVRFTRSGGNATLQVDSWPVIERYPA----  171

Query  61    NERLAIARQRIPYRLGRVVDEWLLDKGRQLTIFNSQATIIIGGKEQGQPFQGQLSGLYYN  120
                                       GRQLTIFNSQA IIIGGKEQGQPFQGQLSGLYYN
Sbjct  172   --------------------------GRQLTIFNSQAPIIIGGKEQGQPFQGQLSGLYYN  273

Query  121   GLKVLNMAA  129
             GL+VL++ +
Sbjct  274   GLEVLDLGS  300


>dbj|HY001490.1| HY001490 RIKEN full-length enriched human cDNA library, testis
Homo sapiens cDNA clone H04D005J11, mRNA sequence. Length=529

 Score =   177 bits (449),  Expect = 4e-54, Method: Compositional matrix adjust.
 Identities = 85/95 (89%), Positives = 89/95 (94%), Gaps = 0/95 (0%)
 Frame = +2

Query  1     QGKIGVKFNVGTDDIAIEESNAIINDGKYHVVRFTRSGGNATLQVDSWPVIERYPAGNND  60
             QG +GV FNVGTDDI I+E NAI++DGKYHVVRFTRSGGNATLQVDSWPV ERYPAGN D
Sbjct  239   QGTVGVIFNVGTDDITIDEPNAIVSDGKYHVVRFTRSGGNATLQVDSWPVNERYPAGNFD  418
```

```
Query  61   NERLAIARQRIPYRLGRVVDEWLLDKGRQLTIFNS  95
            NERLAIARQRIPYRLGRVVDEWLLDKGRQLTIFNS
Sbjct  419  NERLAIARQRIPYRLGRVVDEWLLDKGRQLTIFNS  523
```

```
>dbj|DB476019.1| DB476019 RIKEN full-length enriched human cDNA library, hippocampus
Homo sapiens cDNA clone H023069F15 5', mRNA sequence. Length=487

 Score =   172 bits (437),  Expect = 2e-52, Method: Compositional matrix adjust.
 Identities = 83/85 (98%), Positives = 83/85 (98%), Gaps = 0/85 (0%)
 Frame = +3

Query  64   LAIARQRIPYRLGRVVDEWLLDKGRQLTIFNSQATIIIGGKEQGQPFQGQLSGLYYNGLK  123
            L IARQRIPYRLGRVVDEWLLDKGRQ TIFNSQATIIIGGKEQGQPFQGQLSGLYYNGLK
Sbjct  3    LPIARQRIPYRLGRVVDEWLLDKGRQXTIFNSQATIIIGGKEQGQPFQGQLSGLYYNGLK  182

Query  124  VLNMAAENDANIAIVGNVRLVGEVP  148
            VLNMAAENDANIAIVGNVRLVGEVP
Sbjct  183  VLNMAAENDANIAIVGNVRLVGEVP  257
```

```
>gb|CB154877.1| K-EST0212952 B2N807043 Homo sapiens cDNA clone B2N807043-26-H08
5', mRNA sequence. Length=465

 Score =   166 bits (419),  Expect = 7e-50, Method: Compositional matrix adjust.
 Identities = 85/135 (63%), Positives = 96/135 (71%), Gaps = 30/135 (22%)
 Frame = +3


Query  1    QGKIGVKFNVGTDDIAIEESNAIINDGKYHVVRFTRSGGNATLQVDSWPVIERYPAGNND  60
            QG +GV FNVGTDDI I+E NAI++DGKYHVVRFTRSGGNATLQVDSWPV ERYPA
Sbjct  144  QGTVGVIFNVGTDDITIDEPNAIVSDGKYHVVRFTRSGGNATLQVDSWPVNERYPA----  311

Query  61   NERLAIARQRIPYRLGRVVDEWLLDKGRQLTIFNSQATIIIGGKEQGQPFQGQLSGLYYN  120
                                      GRQLTIFNSQA I IGG++QG+PFQGQ+SGLYYN
Sbjct  312  --------------------------GRQLTIFNSQAAIKIGGRDQGRPFQGQVSGLYYN  413

Query  121  GLKVLNMAAENDANI  135
            GLKVL +AAE+D N+
Sbjct  414  GLKVLALAAESDPNV  458
```

```
>gb|BF345183.1| 602018394F1 NCI_CGAP_Brn67 Homo sapiens cDNA clone IMAGE:4153894
5', mRNA sequence. Length=826

 Score =   161 bits (408),  Expect = 1e-46, Method: Compositional matrix adjust.
 Identities = 88/149 (59%), Positives = 99/149 (66%), Gaps = 31/149 (21%)
 Frame = +3

Query  1    QGKIGVKFNVGTDDIAIEESNAIINDGKYHVVRFTRSGGNATLQVDSWPVIERYPAGNND  60
            QG +GV FNVGTDDI I+E NAI++DGKYHVVRFTRSGGNATLQVDSWPV ERYPA
Sbjct  300  QGTVGVIFNVGTDDITIDEPNAIVSDGKYHVVRFTRSGGNATLQVDSWPVNERYPA----  467

Query  61   NERLAIARQRIPYRLGRVVDEWLLDKGRQLTIFNSQATIIIGGKEQGQPFQGQLSGLYYN  120
                                      GRQLTIFNSQA I IGG++QG+PFQGQ+SGLYYN
Sbjct  468  --------------------------GRQLTIFNSQAAIKIGGRDQGRPFQGQVSGLYYN  569

Query  121  GLKVLNMA-AENDANIAIVGNVRLVGEVP  148
            GLKVL      E    +   G++RLVGE P
Sbjct  570  GLKVLAAGRRERPQMLRTEGHLRLVGEGP  656
```

```
>dbj|DA747306.1| DA747306 NT2RP7 Homo sapiens cDNA clone NT2RP7012472 5', mRNA
sequence. Length=554

 Score =   157 bits (396),  Expect = 5e-46, Method: Compositional matrix adjust.
 Identities = 72/110 (65%), Positives = 88/110 (80%), Gaps = 0/110 (0%)
 Frame = +3

Query  1    QGKIGVKFNVGTDDIAIEESNAIINDGKYHVVRFTRSGGNATLQVDSWPVIERYPAGNND  60
            QGKIGV FN+GT DI+I+E    +NDGKYHVVRFTR+GGNATLQVD+WPV E YP GN D
Sbjct  225  QGKIGVVFNIGTVDISIKEERTPVNDGKYHVVRFTRNGGNATLQVDNWPVNEHYPTGNTD  404

Query  61   NERLAIARQRIPYRLGRVVDEWLLDKGRQLTIFNSQATIIIGGKEQGQPF  110
            NER  + +Q+IP++  R V+EWL +KGRQLTIFN+QA I IGGK++  P
Sbjct  405  NERFQMVKQKIPFKYNRPVEEWLQEKGRQLTIFNTQAQIAIGGKDKRTPL  554


>dbj|HY037592.1| HY037592 RIKEN full-length enriched human cDNA library, testis
Homo sapiens cDNA clone H04D131C20, mRNA sequence. Length=467

 Score =   144 bits (363),  Expect = 2e-41, Method: Compositional matrix adjust.
 Identities = 69/79 (87%), Positives = 73/79 (92%), Gaps = 0/79 (0%)
 Frame = +1

Query  1    QGKIGVKFNVGTDDIAIEESNAIINDGKYHVVRFTRSGGNATLQVDSWPVIERYPAGNND  60
            QG +GV FNVGTDDI I+E NAI++DGKYHVVRFTRSGGNATLQVDSWPV ERYPAGN D
Sbjct  229  QGTVGVIFNVGTDDITIDEPNAIVSDGKYHVVRFTRSGGNATLQVDSWPVNERYPAGNFD  408

Query  61   NERLAIARQRIPYRLGRVV  79
            NERLAIARQRIPYRLGRVV
Sbjct  409  NERLAIARQRIPYRLGRVV  465


>dbj|DB465352.1| DB465352 RIKEN full-length enriched human cDNA library, hippocampus
Homo sapiens cDNA clone H023016C09 5', mRNA sequence. Length=429

 Score =   139 bits (351),  Expect = 8e-40, Method: Compositional matrix adjust.
 Identities = 69/70 (99%), Positives = 69/70 (99%), Gaps = 0/70 (0%)
 Frame = +1

Query  79   VDEWLLDKGRQLTIFNSQATIIIGGKEQGQPFQGQLSGLYYNGLKVLNMAAENDANIAIV  138
            VDEWLLDKGRQLTIFNSQATIIIGGKEQGQPFQGQLSGLYYNGLKVLNMAAENDA IAIV
Sbjct  1    VDEWLLDKGRQLTIFNSQATIIIGGKEQGQPFQGQLSGLYYNGLKVLNMAAENDAKIAIV  180

Query  139  GNVRLVGEVP  148
            GNVRLVGEVP
Sbjct  181  GNVRLVGEVP  210


>gb|BF529228.1| 602041680F1 NCI_CGAP_Brn67 Homo sapiens cDNA clone IMAGE:4179690
5', mRNA sequence. Length=627

 Score = 94.7 bits (234),  Expect(2) = 1e-37, Method: Compositional matrix adjust.
 Identities = 51/102 (50%), Positives = 61/102 (60%), Gaps = 30/102 (29%)
 Frame = +1

Query  47   SWPVIERYPAGNNDNERLAIARQRIPYRLGRVVDEWLLDKGRQLTIFNSQATIIIGGKEQ  106
            +WPV ERYPAG                              RQLTIFNSQA I IGG++Q
Sbjct  193  AWPVNERYPAG------------------------------RQLTIFNSQAAIKIGGRDQ  282

Query  107  GQPFQGQLSGLYYNGLKVLNMAAENDANIAIVGNVRLVGEVP  148
            G+PFQGQ+SGLYYNGLKVL + AE++ N+   G +RLVGE P
Sbjct  283  GRPFQGQVSGLYYNGLKVLALDAESEPNVRTEGQLRLVGEGP  408
```

```
>dbj|HY044110.1| HY044110 RIKEN full-length enriched human cDNA library, testis
Homo sapiens cDNA clone H04D155A10, mRNA sequence. Length=462

 Score =   134 bits (336),  Expect = 2e-37, Method: Compositional matrix adjust.
 Identities = 63/73 (86%), Positives = 67/73 (92%), Gaps = 0/73 (0%)
 Frame = +3

Query  1    QGKIGVKFNVGTDDIAIEESNAIINDGKYHVVRFTRSGGNATLQVDSWPVIERYPAGNND  60
            QG +GV FNVGTDDI I+E NAI++DGKYHVVRFTRSGGNATLQVDSWPV ERYPAGN D
Sbjct  243  QGTVGVIFNVGTDDITIDEPNAIVSDGKYHVVRFTRSGGNATLQVDSWPVNERYPAGNFD  422

Query  61   NERLAIARQRIPY  73
            NERLAIARQRIPY
Sbjct  423  NERLAIARQRIPY  461


>gb|BQ723058.1| AGENCOURT_8109177 Lupski_sympathetic_trunk Homo sapiens cDNA
clone IMAGE:6189515 5', mRNA sequence. Length=921

 Score =   127 bits (319),  Expect = 2e-33, Method: Compositional matrix adjust.
 Identities = 67/115 (58%), Positives = 76/115 (66%), Gaps = 30/115 (26%)
 Frame = +1

Query  34   FTRSGGNATLQVDSWPVIERYPAGNNDNERLAIARQRIPYRLGRVVDEWLLDKGRQLTIF  93
            FTRSGGNATLQVDSWPV ERYPA                               GRQLTIF
Sbjct  1    FTRSGGNATLQVDSWPVNERYPA-------------------------------GRQLTIF  90

Query  94   NSQATIIIGGKEQGQPFQGQLSGLYYNGLKVLNMAAENDANIAIVGNVRLVGEVP  148
            NSQA I IGG++QG+PFQGQ+SGLYYNGLKVL +AAE+D N+   G++RLVGE P
Sbjct  91   NSQAAIKIGGRDQGRPFQGQVSGLYYNGLKVLALAAESDPNVRTEGHLRLVGEGP  255


>gb|BG913108.1| 602811713F1 NCI_CGAP_Brn67 Homo sapiens cDNA clone IMAGE:4943715
5', mRNA sequence. Length=1007

 Score =   120 bits (302),  Expect = 9e-31, Method: Compositional matrix adjust.
 Identities = 55/72 (76%), Positives = 64/72 (89%), Gaps = 0/72 (0%)
 Frame = +1

Query  77   RVVDEWLLDKGRQLTIFNSQATIIIGGKEQGQPFQGQLSGLYYNGLKVLNMAAENDANIA  136
            RVVDEWLLDKGRQLTIFNSQA I IGG++QG+PFQGQ+SGLYYNGLKVL +AAE+D N+
Sbjct  1    RVVDEWLLDKGRQLTIFNSQAAIKIGGRDQGRPFQGQVSGLYYNGLKVLALAAESDPNVR  180

Query  137  IVGNVRLVGEVP  148
              G++RLVGE P
Sbjct  181  TEGHLRLVGEGP  216


>dbj|HY135994.1| HY135994 RIKEN full-length enriched human cDNA library, brain
Homo sapiens cDNA clone H06D115F11, mRNA sequence. Length=480

 Score =   116 bits (290),  Expect = 2e-30, Method: Compositional matrix adjust.
 Identities = 54/71 (76%), Positives = 63/71 (89%), Gaps = 0/71 (0%)
 Frame = +1

Query  78   VVDEWLLDKGRQLTIFNSQATIIIGGKEQGQPFQGQLSGLYYNGLKVLNMAAENDANIAI  137
            VVDEWLLDKGRQLTIFNSQA I IGG++QG+PFQGQ+SGLYYNGLKVL +AAE+D N+
Sbjct  1    VVDEWLLDKGRQLTIFNSQAAIKIGGRDQGRPFQGQVSGLYYNGLKVLALAAESDPNVRT  180

Query  138  VGNVRLVGEVP  148
             G++RLVGE P
Sbjct  181  EGHLRLVGEGP  213
```

```
>gb|AW894889.1| CM1-NN0033-110400-181-e04 NN0033 Homo sapiens cDNA, mRNA sequence.
Length=437

 Score =   100 bits (248),  Expect = 2e-24, Method: Compositional matrix adjust.
 Identities = 46/67 (69%), Positives = 58/67 (87%), Gaps = 0/67 (0%)
 Frame = +1

Query  66   IARQRIPYRLGRVVDEWLLDKGRQLTIFNSQATIIIGGKEQGQPFQGQLSGLYYNGLKVL  125
            +  Q+IP++  R V+EWL +KGRQLTIFN+QA I IGGK++G+ FQGQLSGLYY+GLKVL
Sbjct  118  MVEQKIPFKYNRPVEEWLQEKGRQLTIFNTQAQIAIGGKDKGRLFQGQLSGLYYDGLKVL  297

Query  126  NMAAEND  132
            NMAAEN+
Sbjct  298  NMAAENN  318


>gb|BQ879316.1| AGENCOURT_8113386 Lupski_dorsal_root_ganglion Homo sapiens cDNA
clone IMAGE:6178941 5', mRNA sequence. Length=940

 Score = 99.8 bits (247),  Expect = 3e-23, Method: Compositional matrix adjust.
 Identities = 51/76 (67%), Positives = 57/76 (75%), Gaps = 3/76 (4%)
 Frame = +1

Query  1    QGKIGVKFNVGTDDIAIEESNAIINDGKYHVVRFTRSGGNATLQVDSWPVIERYPAGNND  60
            QG +GV FNVGTDDI I+E NAI++DGKYHVVRFTRSGGNATLQ DSW V ERYP G
Sbjct  334  QGTVGVIFNVGTDDITIDEPNAIVSDGKYHVVRFTRSGGNATLQADSWTVNERYP-GQPP  510

Query  61   NERLAIARQRIPYRLG  76
             E L   + R+P R G
Sbjct  511  AEHL--QQPRLPSRSG  552


>dbj|DA745012.1| DA745012 NT2RP7 Homo sapiens cDNA clone NT2RP7009828 5', mRNA
sequence. Length=564

 Score = 94.4 bits (233),  Expect = 8e-22, Method: Compositional matrix adjust.
 Identities = 51/97 (53%), Positives = 58/97 (60%), Gaps = 30/97 (31%)
 Frame = +2

Query  1    QGKIGVKFNVGTDDIAIEESNAIINDGKYHVVRFTRSGGNATLQVDSWPVIERYPAGNND  60
            QGKIGV FN+GT DI+I+E    +NDGKYHVVRFTR+GGNATLQVD+WPV E YP
Sbjct  362  QGKIGVVFNIGTVDISIKEERTPVNDGKYHVVRFTRNGGNATLQVDNWPVNEHYPT----  529

Query  61   NERLAIARQRIPYRLGRVVDEWLLDKGRQLTIFNSQA  97
                                      GRQLTIFN+QA
Sbjct  530  --------------------------GRQLTIFNTQA  562
>gb|AA984057.1| am76b03.s1 Stratagene schizo brain S11 Homo sapiens cDNA clone
IMAGE:1616333 3' similar to TR:Q63376 Q63376 NEUREXIN II-BETA-A
PRECURSOR. ;, mRNA sequence. Length=728

 Score = 63.9 bits (154),  Expect(2) = 4e-21, Method: Compositional matrix adjust.
 Identities = 42/109 (39%), Positives = 51/109 (47%), Gaps = 31/109 (28%)
 Frame = -2

Query  40   NATLQVDSWPVIERYPAGNNDNERLAIARQRIPYRLGRVVDEWLLDKGRQLTIFNSQATI  99
            N TLQVDSWPV ERY AG                              RQLTIFNSQA I
Sbjct  508  NRTLQVDSWPVNERYAAG------------------------------RQLTIFNSQAAI  419

Query  100  IIGGKEQGQPFQGQLSGLYYNGLKVLNMAAENDANIAIVGNVRLVGEVP  148
             I G++QG+P +         G +      AE+D N+    ++RLVGE P
Sbjct  418  KIVGRDQGRPSRPVCPASTTMGSRCC-AGAESDPNVRTECHLRLVGEGP  275
```

Mus Musculus (4/27 splice variants)

```
                                                                 Score     E
Sequences producing significant alignments:                      (Bits)  Value

gb|CF553803.1|  AGENCOURT_15611286 Organ of Corti Mus musculus...    283   7e-94
gb|CB249743.1|  UI-M-EX0-byl-b-10-0-UI.r1 NIH_BMAP_EX0 Mus mus...    253   1e-82
gb|BM944179.1|  UI-M-EH0p-bvq-g-21-0-UI.r1 NIH_BMAP_EH0p Mus m...    249   8e-82
gb|BM950483.1|  UI-M-EH0p-buu-o-21-0-UI.r1 NIH_BMAP_EH0p Mus m...    244   1e-80
gb|DT927517.1|  PCRS30340 Hematopoietic stem cells Mus musculu...    241   1e-78
gb|CX208193.1|  MNS13294 Mouse Neurosphere Normalized cDNA lib...    239   4e-78
gb|CX217204.1|  MNS31265 Mouse Neurosphere Normalized cDNA lib...    236   5e-77
gb|DT924042.1|  PCRS10316 Hematopoietic stem cells Mus musculu...    233   4e-76
gb|DT925356.1|  PCRS50408 Hematopoietic stem cells Mus musculu...    233   7e-76
gb|BU056634.1|  UI-M-FO0-cac-b-20-0-UI.r1 NIH_BMAP_FO0 Mus mus...    229   7e-74
gb|CX206076.1|  MNS10169 Mouse Neurosphere Normalized cDNA lib...    226   3e-73
gb|CF743000.1|  UI-M-GV0-cln-a-15-0-UI.r1 NIH_BMAP_GV0 Mus mus...    226   9e-73
gb|CF743053.1|  UI-M-GV0-cln-k-17-0-UI.r1 NIH_BMAP_GV0 Mus mus...    226   3e-72
gb|CX202063.1|  MNS03719 Mouse Neurosphere Normalized cDNA lib...    221   3e-71
gb|CF743182.1|  UI-M-GV0-clo-c-19-0-UI.r1 NIH_BMAP_GV0 Mus mus...    199   2e-61
gb|CX217588.1|  MNS31680 Mouse Neurosphere Normalized cDNA lib...    153   2e-60
gb|CA328232.1|  UI-M-FY0-cdb-m-23-0-UI.r1 NIH_BMAP_FY0 Mus mus...    195   3e-60
gb|CX215555.1|  MNS25539 Mouse Neurosphere Normalized cDNA lib...    187   1e-58
gb|CX223578.1|  MNS41923 Mouse Neurosphere Normalized cDNA lib...    184   3e-57
gb|CX199726.1|  MNS08623 Mouse Neurosphere Normalized cDNA lib...    184   3e-56
gb|CX216431.1|  MNS29780 Mouse Neurosphere Normalized cDNA lib...    182   5e-56
gb|DN173237.1|  NMB07261 Mus Musculus Lateral Ventricle Wall C...    182   6e-56
gb|CA327544.1|  UI-M-FY0-ccz-i-02-0-UI.r1 NIH_BMAP_FY0 Mus mus...    178   7e-54
dbj|BB858994.1|  BB858994 RIKEN full-length enriched, brain CR...    145   5e-42
gb|DT904887.1|  BMSB13606 Hematopoietic stem cells Mus musculu...    144   8e-41
gb|CX220555.1|  MNS36241 Mouse Neurosphere Normalized cDNA lib...    100   2e-37
gb|CX212965.1|  MNS18219 Mouse Neurosphere Normalized cDNA lib...    129   2e-35
gb|CX203319.1|  MNS05584 Mouse Neurosphere Normalized cDNA lib...    125   1e-33
dbj|BP427400.1|  BP427400 Mus musculus cerebellum E18-P56 Mus ...    120   2e-32
dbj|BB692115.1|  BB692115 RIKEN full-length enriched, 2 days n...    119   7e-32
dbj|BB695296.1|  BB695296 RIKEN full-length enriched, 2 days n...    115   2e-30
dbj|BB130803.1|  BB130803 RIKEN full-length enriched, 16 days ...    109   4e-29
gb|DT915246.1|  BMSB12312 Hematopoietic stem cells Mus musculu...    111   9e-28
dbj|BY632421.1|  BY632421 RIKEN full-length enriched, visual c...    105   1e-26
gb|DT906469.1|  PCRS50341 Hematopoietic stem cells Mus musculu...    100   5e-25

ALIGNMENTS
>gb|CF553803.1| AGENCOURT_15611286 Organ of Corti Mus musculus cDNA clone
IMAGE:30519294 5', mRNA sequence. Length=925

 Score =   283 bits (723),  Expect = 7e-94, Method: Compositional matrix adjust.
 Identities = 135/136 (99%), Positives = 136/136 (100%), Gaps = 0/136 (0%)
 Frame = +2

Query  13   DDIAIEESNAIINDGKYHVVRFTRSGGNATLQVDSWPVIERYPAGNNDNERLAIARQRIP  72
            DDIAIEESNAIINDGKYHVVRFTRSGGNATLQVDSWPVIERYPAGNNDNERLAIARQRIP
Sbjct  8    DDIAIEESNAIINDGKYHVVRFTRSGGNATLQVDSWPVIERYPAGNNDNERLAIARQRIP  187

Query  73   YRLGRVVDEWLLDKGRQLTIFNSQATIIIGGKEQGQPFQGQLSGLYYNGLKVLNMAAEND  132
            YRLGRVVDEWLLDKGRQLTIFNSQATIIIGGKEQGQPFQGQLSGLYYNGLKVLNMAAEND
Sbjct  188  YRLGRVVDEWLLDKGRQLTIFNSQATIIIGGKEQGQPFQGQLSGLYYNGLKVLNMAAEND  367

Query  133  ANIAIVGNVRLVGEVP  148
            ANIAIVGNV+LVGEVP
Sbjct  368  ANIAIVGNVKLVGEVP  415
```

```
>gb|CB249743.1| UI-M-EX0-byl-b-10-0-UI.r1 NIH_BMAP_EX0 Mus musculus cDNA clone
IMAGE:5719593 5', mRNA sequence. Length=881

 Score =   253 bits (647),  Expect = 1e-82, Method: Compositional matrix adjust.
 Identities = 121/148 (82%), Positives = 134/148 (91%), Gaps = 0/148 (0%)
 Frame = +3

Query  1    QGKIGVKFNVGTDDIAIEESNAIINDGKYHVVRFTRSGGNATLQVDSWPVIERYPAGNND  60
            QG +GV FNVGTDDI I+E NAI++DGKYHVVRFTRSGGNATLQVDSWPV ERYPAGN D
Sbjct  69   QGTVGVIFNVGTDDITIDEPNAIVSDGKYHVVRFTRSGGNATLQVDSWPVNERYPAGNFD  248

Query  61   NERLAIARQRIPYRLGRVVDEWLLDKGRQLTIFNSQATIIIGGKEQGQPFQGQLSGLYYN  120
            NERLAIARQRIPYRLGRVVDEWLLDKGRQLTIFNSQA I IGG++QG+PFQGQ+SGLYYN
Sbjct  249  NERLAIARQRIPYRLGRVVDEWLLDKGRQLTIFNSQAAIKIGGRDQGRPFQGQVSGLYYN  428

Query  121  GLKVLNMAAENDANIAIVGNVRLVGEVP  148
            GLKVL +AAE+D N+   G++RLVGE P
Sbjct  429  GLKVLALAAESDPNVRTEGHLRLVGEGP  512


>gb|BM944179.1| UI-M-EH0p-bvq-g-21-0-UI.r1 NIH_BMAP_EH0p Mus musculus cDNA clone
IMAGE:5695532 5', mRNA sequence. Length=711

 Score =   249 bits (636),  Expect = 8e-82, Method: Compositional matrix adjust.
 Identities = 119/145 (82%), Positives = 132/145 (91%), Gaps = 0/145 (0%)
 Frame = +2

Query  4    IGVKFNVGTDDIAIEESNAIINDGKYHVVRFTRSGGNATLQVDSWPVIERYPAGNNDNER  63
            +GV FNVGTDDI I+E NAI++DGKYHVVRFTRSGGNATLQVDSWPV ERYPAGN DNER
Sbjct  8    VGVIFNVGTDDITIDEPNAIVSDGKYHVVRFTRSGGNATLQVDSWPVNERYPAGNFDNER  187

Query  64   LAIARQRIPYRLGRVVDEWLLDKGRQLTIFNSQATIIIGGKEQGQPFQGQLSGLYYNGLK  123
            LAIARQRIPYRLGRVVDEWLLDKGRQLTIFNSQA I IGG++QG+PFQGQ+SGLYYNGLK
Sbjct  188  LAIARQRIPYRLGRVVDEWLLDKGRQLTIFNSQAAIKIGGRDQGRPFQGQVSGLYYNGLK  367

Query  124  VLNMAAENDANIAIVGNVRLVGEVP  148
            VL +AAE+D N+   G++RLVGE P
Sbjct  368  VLALAAESDPNVRTEGHLRLVGEGP  442


>gb|BM950483.1| UI-M-EH0p-buu-o-21-0-UI.r1 NIH_BMAP_EH0p Mus musculus cDNA clone
IMAGE:5687276 5', mRNA sequence. Length=590

 Score =   244 bits (624),  Expect = 1e-80, Method: Compositional matrix adjust.
 Identities = 118/145 (81%), Positives = 131/145 (90%), Gaps = 0/145 (0%)
 Frame = +1

Query  4    IGVKFNVGTDDIAIEESNAIINDGKYHVVRFTRSGGNATLQVDSWPVIERYPAGNNDNER  63
            +GV FNVGTDDI I+E NAI++DGKYHVVRFTRSGGNATLQVDSWPV ERYPAGN DNER
Sbjct  46   LGVIFNVGTDDITIDEPNAIVSDGKYHVVRFTRSGGNATLQVDSWPVNERYPAGNFDNER  225

Query  64   LAIARQRIPYRLGRVVDEWLLDKGRQLTIFNSQATIIIGGKEQGQPFQGQLSGLYYNGLK  123
            LAIARQRIPYRLGRVVDEWLLDKGRQLTIFNSQA I IGG++QG+PFQGQ+SGLYYNGLK
Sbjct  226  LAIARQRIPYRLGRVVDEWLLDKGRQLTIFNSQAAIKIGGRDQGRPFQGQVSGLYYNGLK  405

Query  124  VLNMAAENDANIAIVGNVRLVGEVP  148
            VL +AAE+D N+    ++RLVGE P
Sbjct  406  VLALAAESDPNVRTECHLRLVGEGP  480
```

```
>gb|DT927517.1| PCRS30340 Hematopoietic stem cells Mus musculus cDNA, mRNA sequence.
Length=699

 Score =   241 bits (615),  Expect = 1e-78, Method: Compositional matrix adjust.
 Identities = 116/116 (100%), Positives = 116/116 (100%), Gaps = 0/116 (0%)
 Frame = +2

Query  33   RFTRSGGNATLQVDSWPVIERYPAGNNDNERLAIARQRIPYRLGRVVDEWLLDKGRQLTI  92
            RFTRSGGNATLQVDSWPVIERYPAGNNDNERLAIARQRIPYRLGRVVDEWLLDKGRQLTI
Sbjct  2    RFTRSGGNATLQVDSWPVIERYPAGNNDNERLAIARQRIPYRLGRVVDEWLLDKGRQLTI  181

Query  93   FNSQATIIIGGKEQGQPFQGQLSGLYYNGLKVLNMAAENDANIAIVGNVRLVGEVP  148
            FNSQATIIIGGKEQGQPFQGQLSGLYYNGLKVLNMAAENDANIAIVGNVRLVGEVP
Sbjct  182  FNSQATIIIGGKEQGQPFQGQLSGLYYNGLKVLNMAAENDANIAIVGNVRLVGEVP  349


>gb|CX208193.1| MNS13294 Mouse Neurosphere Normalized cDNA library Mus musculus
cDNA 5', mRNA sequence. Length=670

 Score =   239 bits (611),  Expect = 4e-78, Method: Compositional matrix adjust.
 Identities = 114/135 (84%), Positives = 125/135 (93%), Gaps = 0/135 (0%)
 Frame = +2

Query  1    QGKIGVKFNVGTDDIAIEESNAIINDGKYHVVRFTRSGGNATLQVDSWPVIERYPAGNND  60
            QG +GV FNVGTDDI I+E NAI++DGKYHVVRFTRSGGNATLQVDSWPV ERYPAGN D
Sbjct  257  QGTVGVIFNVGTDDITIDEPNAIVSDGKYHVVRFTRSGGNATLQVDSWPVNERYPAGNFD  436

Query  61   NERLAIARQRIPYRLGRVVDEWLLDKGRQLTIFNSQATIIIGGKEQGQPFQGQLSGLYYN  120
            NERLAIARQRIPYRLGRVVDEWLLDKGRQLTIFNSQA I IGG++QG+PFQGQ+SGLYYN
Sbjct  437  NERLAIARQRIPYRLGRVVDEWLLDKGRQLTIFNSQAAIKIGGRDQGRPFQGQVSGLYYN  616

Query  121  GLKVLNMAAENDANI  135
            GLKVL +AAE+D N+
Sbjct  617  GLKVLALAAESDPNV  661


>gb|CX217204.1| MNS31265 Mouse Neurosphere Normalized cDNA library Mus musculus
cDNA 5', mRNA sequence. Length=642

 Score =   236 bits (602),  Expect = 5e-77, Method: Compositional matrix adjust.
 Identities = 112/135 (83%), Positives = 123/135 (91%), Gaps = 0/135 (0%)
 Frame = +1

Query  1    QGKIGVKFNVGTDDIAIEESNAIINDGKYHVVRFTRSGGNATLQVDSWPVIERYPAGNND  60
             G +GV FNVGTDDI I+E NAI++DGKYHVVRFTRSGGNATLQVDSWPV ERYPAGN D
Sbjct  220  HGTVGVIFNVGTDDITIDEPNAIVSDGKYHVVRFTRSGGNATLQVDSWPVNERYPAGNFD  399

Query  61   NERLAIARQRIPYRLGRVVDEWLLDKGRQLTIFNSQATIIIGGKEQGQPFQGQLSGLYYN  120
            NERLAIARQRIPYRLGRVVDEWLLDKGRQLTIFNSQA I IGG++ G+PFQGQ+SGLYYN
Sbjct  400  NERLAIARQRIPYRLGRVVDEWLLDKGRQLTIFNSQAAIKIGGRDHGRPFQGQVSGLYYN  579

Query  121  GLKVLNMAAENDANI  135
            GLKVL +AAE+D N+
Sbjct  580  GLKVLALAAESDPNV  624


>gb|DT924042.1| PCRS10316 Hematopoietic stem cells Mus musculus cDNA, mRNA sequence.
Length=563

 Score =   233 bits (594),  Expect = 4e-76, Method: Compositional matrix adjust.
 Identities = 113/116 (97%), Positives = 113/116 (97%), Gaps = 0/116 (0%)
 Frame = +2
```

```
Query  33   RFTRSGGNATLQVDSWPVIERYPAGNNDNERLAIARQRIPYRLGRVVDEWLLDKGRQLTI  92
            RFTRSGGNATLQVDSWPVIERYPAGNNDNE  AIARQR PYRLGRVVDEWLLDKGRQLTI
Sbjct  2    RFTRSGGNATLQVDSWPVIERYPAGNNDNECPAIARQRFPYRLGRVVDEWLLDKGRQLTI  181

Query  93   FNSQATIIIGGKEQGQPFQGQLSGLYYNGLKVLNMAAENDANIAIVGNVRLVGEVP  148
            FNSQATIIIGGKEQGQPFQGQLSGLYYNGLKVLNMAAENDANIAIVGNVRLVGEVP
Sbjct  182  FNSQATIIIGGKEQGQPFQGQLSGLYYNGLKVLNMAAENDANIAIVGNVRLVGEVP  349


>gb|DT925356.1| PCRS50408 Hematopoietic stem cells Mus musculus cDNA, mRNA sequence.
Length=635

 Score =  233 bits (595),  Expect = 7e-76, Method: Compositional matrix adjust.
 Identities = 113/116 (97%), Positives = 115/116 (99%), Gaps = 0/116 (0%)
 Frame = +2

Query  33   RFTRSGGNATLQVDSWPVIERYPAGNNDNERLAIARQRIPYRLGRVVDEWLLDKGRQLTI  92
            RFTRSGGNATLQVDSWP IERYPAGNNDNERLA+ARQRIPYRLGRVVDEWLLD+GRQLTI
Sbjct  2    RFTRSGGNATLQVDSWPFIERYPAGNNDNERLAMARQRIPYRLGRVVDEWLLDEGRQLTI  181

Query  93   FNSQATIIIGGKEQGQPFQGQLSGLYYNGLKVLNMAAENDANIAIVGNVRLVGEVP  148
            FNSQATIIIGGKEQGQPFQGQLSGLYYNGLKVLNMAAENDANIAIVGNVRLVGEVP
Sbjct  182  FNSQATIIIGGKEQGQPFQGQLSGLYYNGLKVLNMAAENDANIAIVGNVRLVGEVP  349


>gb|BU056634.1| UI-M-FO0-cac-b-20-0-UI.r1 NIH_BMAP_FO0 Mus musculus cDNA clone
IMAGE:6409267 5', mRNA sequence. Length=710

 Score =  229 bits (584),  Expect = 7e-74, Method: Compositional matrix adjust.
 Identities = 109/132 (83%), Positives = 119/132 (90%), Gaps = 0/132 (0%)
 Frame = +2

Query  1    QGKIGVKFNVGTDDIAIEESNAIINDGKYHVVRFTRSGGNATLQVDSWPVIERYPAGNND  60
            QG +GV FNVGTDDI I+E NAI++DGKYHVVRFTRSGGNATLQVDSWPV ERYPAGN D
Sbjct  314  QGTVGVIFNVGTDDITIDEPNAIVSDGKYHVVRFTRSGGNATLQVDSWPVNERYPAGNFD  493

Query  61   NERLAIARQRIPYRLGRVVDEWLLDKGRQLTIFNSQATIIIGGKEQGQPFQGQLSGLYYN  120
            NERLAIARQRI YRLGRVVDEWLLDKGRQLTIFNSQA I IGG++QG+PF GQ+SGLYY
Sbjct  494  NERLAIARQRISYRLGRVVDEWLLDKGRQLTIFNSQAAIKIGGRDQGRPFHGQVSGLYYT  673

Query  121  GLKVLNMAAEND  132
            GL VL +AAE+D
Sbjct  674  GLTVLALAAESD  709


>gb|CX206076.1| MNS10169 Mouse Neurosphere Normalized cDNA library Mus musculus
cDNA 5', mRNA sequence. Length=621

 Score =  226 bits (577),  Expect = 3e-73, Method: Compositional matrix adjust.
 Identities = 109/125 (87%), Positives = 117/125 (94%), Gaps = 0/125 (0%)
 Frame = +3

Query  1    QGKIGVKFNVGTDDIAIEESNAIINDGKYHVVRFTRSGGNATLQVDSWPVIERYPAGNND  60
            QG +GV FNVGTDDI I+E NAI++DGKYHVVRFTRSGGNATLQVDSWPV ERYPAGN D
Sbjct  222  QGTVGVIFNVGTDDITIDEPNAIVSDGKYHVVRFTRSGGNATLQVDSWPVNERYPAGNFD  401

Query  61   NERLAIARQRIPYRLGRVVDEWLLDKGRQLTIFNSQATIIIGGKEQGQPFQGQLSGLYYN  120
            NERLAIARQRIPYRLGRVVDEWLLDKGRQLTIFNSQA I IGG++QG+PFQGQ+SGLYYN
Sbjct  402  NERLAIARQRIPYRLGRVVDEWLLDKGRQLTIFNSQAAIKIGGRDQGRPFQGQVSGLYYN  581

Query  121  GLKVL  125
```

```
            GLKVL
Sbjct  582  GLKVL  596


>gb|CF743000.1| UI-M-GV0-cln-a-15-0-UI.r1 NIH_BMAP_GV0 Mus musculus cDNA clone
IMAGE:30616238 5', mRNA sequence. Length=734

 Score =   226 bits (577),  Expect = 9e-73, Method: Compositional matrix adjust.
 Identities = 118/148 (80%), Positives = 118/148 (80%), Gaps = 30/148 (20%)
 Frame = +2

Query  1    QGKIGVKFNVGTDDIAIEESNAIINDGKYHVVRFTRSGGNATLQVDSWPVIERYPAGNND  60
            QGKIGVKFNVGTDDIAIEESNAIINDGKYHVVRFTRSGGNATLQVDSWPVIERYPAG
Sbjct  296  QGKIGVKFNVGTDDIAIEESNAIINDGKYHVVRFTRSGGNATLQVDSWPVIERYPAG---  466

Query  61   NERLAIARQRIPYRLGRVVDEWLLDKGRQLTIFNSQATIIIGGKEQGQPFQGQLSGLYYN  120
                                       RQLTIFNSQATIIIGGKEQGQPFQGQLSGLYYN
Sbjct  467  ---------------------------RQLTIFNSQATIIIGGKEQGQPFQGQLSGLYYN  565

Query  121  GLKVLNMAAENDANIAIVGNVRLVGEVP  148
            GLKVLNMAAENDANIAIVGNVRLVGEVP
Sbjct  566  GLKVLNMAAENDANIAIVGNVRLVGEVP  649


>gb|CF743053.1| UI-M-GV0-cln-k-17-0-UI.r1 NIH_BMAP_GV0 Mus musculus cDNA clone
IMAGE:30616480 5', mRNA sequence. Length=751

 Score =   226 bits (575),  Expect = 3e-72, Method: Compositional matrix adjust.
 Identities = 118/148 (80%), Positives = 118/148 (80%), Gaps = 30/148 (20%)
 Frame = +2

Query  1    QGKIGVKFNVGTDDIAIEESNAIINDGKYHVVRFTRSGGNATLQVDSWPVIERYPAGNND  60
            QGKIGVKFNVGTDDIAIEESNAIINDGKYHVVRFTRSGGNATLQVDSWPVIERYPAG
Sbjct  296  QGKIGVKFNVGTDDIAIEESNAIINDGKYHVVRFTRSGGNATLQVDSWPVIERYPAG---  466

Query  61   NERLAIARQRIPYRLGRVVDEWLLDKGRQLTIFNSQATIIIGGKEQGQPFQGQLSGLYYN  120
                                       RQLTIFNSQATIIIGGKEQGQPFQGQLSGLYYN
Sbjct  467  ---------------------------RQLTIFNSQATIIIGGKEQGQPFQGQLSGLYYN  565

Query  121  GLKVLNMAAENDANIAIVGNVRLVGEVP  148
            GLKVLNMAAENDANIAIVGNVRLVGEVP
Sbjct  566  GLKVLNMAAENDANIAIVGNVRLVGEVP  649


>gb|CX202063.1| MNS03719 Mouse Neurosphere Normalized cDNA library Mus musculus
cDNA 5', mRNA sequence. Length=627

 Score =   221 bits (564),  Expect = 3e-71, Method: Compositional matrix adjust.
 Identities = 106/123 (86%), Positives = 115/123 (93%), Gaps = 0/123 (0%)
 Frame = +2

Query  1    QGKIGVKFNVGTDDIAIEESNAIINDGKYHVVRFTRSGGNATLQVDSWPVIERYPAGNND  60
            QG +GV FNVGTDDI I+E NAI++DGKYHVVRFTRSGGNATLQVDSWPV ERYPAGN D
Sbjct  227  QGTVGVIFNVGTDDITIDEPNAIVSDGKYHVVRFTRSGGNATLQVDSWPVNERYPAGNFD  406

Query  61   NERLAIARQRIPYRLGRVVDEWLLDKGRQLTIFNSQATIIIGGKEQGQPFQGQLSGLYYN  120
            NERLAIARQRIPYRLGRVV+EWLLDKGRQLTIFNSQA I IGG++QG+PFQGQ+SGLYYN
Sbjct  407  NERLAIARQRIPYRLGRVVNEWLLDKGRQLTIFNSQAAIKIGGRDQGRPFQGQVSGLYYN  586

Query  121  GLK  123
            GLK
Sbjct  587  GLK  595
```

```
>gb|CF743182.1| UI-M-GV0-clo-c-19-0-UI.r1 NIH_BMAP_GV0 Mus musculus cDNA clone
IMAGE:30621282 5', mRNA sequence. Length=833

 Score =   199 bits (505),  Expect = 2e-61, Method: Compositional matrix adjust.
 Identities = 93/130 (72%), Positives = 111/130 (85%), Gaps = 0/130 (0%)
 Frame = +3

Query  1    QGKIGVKFNVGTDDIAIEESNAIINDGKYHVVRFTRSGGNATLQVDSWPVIERYPAGNND  60
            QGKIGV FN+GT DI+I+E    +NDGKYHVVRFTR+GGNATLQVD+WPV E YP GN D
Sbjct  300  QGKIGVVFNIGTVDISIKEERTPVNDGKYHVVRFTRNGGNATLQVDNWPVNEHYPTGNTD  479

Query  61   NERLAIARQRIPYRLGRVVDEWLLDKGRQLTIFNSQATIIIGGKEQGQPFQGQLSGLYYN  120
            NERL + +Q+IP++  R V+EWL +KGRQLTIFN+QA I IGGK++G+ FQGQLSGLYY+
Sbjct  480  NERLQMVKQKIPFKYNRPVEEWLQEKGRQLTIFNTQAQIAIGGKDKGRLFQGQLSGLYYD  659

Query  121  GLKVLNMAAE  130
            GLKVLNMAAE
Sbjct  660  GLKVLNMAAE  689


>gb|CX217588.1| MNS31680 Mouse Neurosphere Normalized cDNA library Mus musculus
cDNA 5', mRNA sequence. Length=765

 Score =   153 bits (386),  Expect(2) = 2e-60, Method: Compositional matrix adjust.
 Identities = 72/82 (88%), Positives = 76/82 (93%), Gaps = 0/82 (0%)
 Frame = +3

Query  1    QGKIGVKFNVGTDDIAIEESNAIINDGKYHVVRFTRSGGNATLQVDSWPVIERYPAGNND  60
            QG +GV FNVGTDDI I+E NAI++DGKYHVVRFTRSGGNATLQVDSWPV ERYPAGN D
Sbjct  42   QGTVGVIFNVGTDDITIDEPNAIVSDGKYHVVRFTRSGGNATLQVDSWPVNERYPAGNFD  221

Query  61   NERLAIARQRIPYRLGRVVDEW  82
            NERLAIARQRIPYRLGRVVDEW
Sbjct  222  NERLAIARQRIPYRLGRVVDEW  287


>gb|CA328232.1| UI-M-FY0-cdb-m-23-0-UI.r1 NIH_BMAP_FY0 Mus musculus cDNA clone
IMAGE:6827424 5', mRNA sequence. Length=790

 Score =   195 bits (496),  Expect = 3e-60, Method: Compositional matrix adjust.
 Identities = 93/109 (85%), Positives = 100/109 (92%), Gaps = 0/109 (0%)
 Frame = +1

Query  1    QGKIGVKFNVGTDDIAIEESNAIINDGKYHVVRFTRSGGNATLQVDSWPVIERYPAGNND  60
            QG +GV FNVGTDDI I+E NAI++DGKYHVVRFTRSGGNATLQVDSWPV ERYPAGN D
Sbjct  382  QGTVGVIFNVGTDDITIDEPNAIVSDGKYHVVRFTRSGGNATLQVDSWPVNERYPAGNFD  561

Query  61   NERLAIARQRIPYRLGRVVDEWLLDKGRQLTIFNSQATIIIGGKEQGQP  109
            NERLAIARQRIPYRLGRVV EWLLDKGRQLTIFNSQA I IGG++QG+P
Sbjct  562  NERLAIARQRIPYRLGRVVXEWLLDKGRQLTIFNSQAAIKIGGRDQGRP  708


>gb|CX215555.1| MNS25539 Mouse Neurosphere Normalized cDNA library Mus musculus
cDNA 5', mRNA sequence. Length=401

 Score =   187 bits (474),  Expect = 1e-58, Method: Compositional matrix adjust.
 Identities = 90/90 (100%), Positives = 90/90 (100%), Gaps = 0/90 (0%)
 Frame = +1

Query  1    QGKIGVKFNVGTDDIAIEESNAIINDGKYHVVRFTRSGGNATLQVDSWPVIERYPAGNND  60
```

```
                QGKIGVKFNVGTDDIAIEESNAIINDGKYHVVRFTRSGGNATLQVDSWPVIERYPAGNND
Sbjct  130   QGKIGVKFNVGTDDIAIEESNAIINDGKYHVVRFTRSGGNATLQVDSWPVIERYPAGNND  309

Query  61    NERLAIARQRIPYRLGRVVDEWLLDKGRQL  90
             NERLAIARQRIPYRLGRVVDEWLLDKGRQL
Sbjct  310   NERLAIARQRIPYRLGRVVDEWLLDKGRQL  399


>gb|CX223578.1| MNS41923 Mouse Neurosphere Normalized cDNA library Mus musculus
cDNA 5', mRNA sequence. Length=503

 Score =   184 bits (467),  Expect = 3e-57, Method: Compositional matrix adjust.
 Identities = 89/101 (88%), Positives = 93/101 (92%), Gaps = 0/101 (0%)
 Frame = +1

Query  1     QGKIGVKFNVGTDDIAIEESNAIINDGKYHVVRFTRSGGNATLQVDSWPVIERYPAGNND  60
             QG +GV FNVGTDDI I+E NAI++DGKYHVVRFTRSGGNATLQVDSWPV ERYPAGN D
Sbjct  151   QGTVGVIFNVGTDDITIDEPNAIVSDGKYHVVRFTRSGGNATLQVDSWPVNERYPAGNFD  330

Query  61    NERLAIARQRIPYRLGRVVDEWLLDKGRQLTIFNSQATIII  101
             NERLAIARQRIPYRLGRVVDEWLLDKGRQLTIFNSQA I I
Sbjct  331   NERLAIARQRIPYRLGRVVDEWLLDKGRQLTIFNSQAAIKI  453


>gb|CX199726.1| MNS08623 Mouse Neurosphere Normalized cDNA library Mus musculus
cDNA 5', mRNA sequence. Length=747

 Score =   184 bits (467),  Expect = 3e-56, Method: Compositional matrix adjust.
 Identities = 89/101 (88%), Positives = 93/101 (92%), Gaps = 0/101 (0%)
 Frame = +3

Query  1     QGKIGVKFNVGTDDIAIEESNAIINDGKYHVVRFTRSGGNATLQVDSWPVIERYPAGNND  60
             QG +GV FNVGTDDI I+E NAI++DGKYHVVRFTRSGGNATLQVDSWPV ERYPAGN D
Sbjct  348   QGTVGVIFNVGTDDITIDEPNAIVSDGKYHVVRFTRSGGNATLQVDSWPVNERYPAGNFD  527

Query  61    NERLAIARQRIPYRLGRVVDEWLLDKGRQLTIFNSQATIII  101
             NERLAIARQRIPYRLGRVVDEWLLDKGRQLTIFNSQA I I
Sbjct  528   NERLAIARQRIPYRLGRVVDEWLLDKGRQLTIFNSQAAIRI  650


>gb|CX216431.1| MNS29780 Mouse Neurosphere Normalized cDNA library Mus musculus
cDNA 5', mRNA sequence. Length=588

 Score =   182 bits (462),  Expect = 5e-56, Method: Compositional matrix adjust.
 Identities = 88/101 (87%), Positives = 92/101 (91%), Gaps = 0/101 (0%)
 Frame = +3

Query  1     QGKIGVKFNVGTDDIAIEESNAIINDGKYHVVRFTRSGGNATLQVDSWPVIERYPAGNND  60
             QG +GV FNVGTDDI I+E NAI++DGKYHVVRFTRSGGNATLQVDSWPV ERYPAGN D
Sbjct  240   QGTVGVIFNVGTDDITIDEPNAIVSDGKYHVVRFTRSGGNATLQVDSWPVNERYPAGNFD  419

Query  61    NERLAIARQRIPYRLGRVVDEWLLDKGRQLTIFNSQATIII  101
             NERLAIARQRIPYRLGRVV EWLLDKGRQLTIFNSQA I I
Sbjct  420   NERLAIARQRIPYRLGRVVHEWLLDKGRQLTIFNSQAAIKI  542


>gb|DN173237.1| NMB07261 Mus Musculus Lateral Ventricle Wall C57BL/6 adult Mus
musculus cDNA 5', mRNA sequence. Length=631

 Score =   182 bits (462),  Expect = 6e-56, Method: Compositional matrix adjust.
 Identities = 88/106 (83%), Positives = 97/106 (92%), Gaps = 0/106 (0%)
 Frame = +3
```

```
Query  41   ATLQVDSWPVIERYPAGNNDNERLAIARQRIPYRLGRVVDEWLLDKGRQLTIFNSQATII  100
            ATLQVDSWPV ERYPAGN DNERLAIARQRIPYRLGRVVDEWLLDKGRQLTIFNSQA I
Sbjct  3    ATLQVDSWPVNERYPAGNFDNERLAIARQRIPYRLGRVVDEWLLDKGRQLTIFNSQAAIK  182

Query  101  IGGKEQGQPFQGQLSGLYYNGLKVLNMAAENDANIAIVGNVRLVGE  146
            IGG++QG+PFQGQ+SGLYYNGLKVL +AAE+D N+   G++RLVGE
Sbjct  183  IGGRDQGRPFQGQVSGLYYNGLKVLALAAESDPNVRTEGHLRLVGE  320


>gb|CA327544.1| UI-M-FY0-ccz-i-02-0-UI.r1 NIH_BMAP_FY0 Mus musculus cDNA clone
IMAGE:6826539 5', mRNA sequence. Length=766

 Score =   178 bits (452),  Expect = 7e-54, Method: Compositional matrix adjust.
 Identities = 92/148 (62%), Positives = 105/148 (71%), Gaps = 30/148 (20%)
 Frame = +1

Query  1    QGKIGVKFNVGTDDIAIEESNAIINDGKYHVVRFTRSGGNATLQVDSWPVIERYPAGNND  60
            QG +GV FNVGTDDI I+E NAI++DGKYHVVRFTRSGGNATLQVDSWPV ERYPA
Sbjct  244  QGTVGVIFNVGTDDITIDEPNAIVSDGKYHVVRFTRSGGNATLQVDSWPVNERYPA----  411

Query  61   NERLAIARQRIPYRLGRVVDEWLLDKGRQLTIFNSQATIIIGGKEQGQPFQGQLSGLYYN  120
                                      GRQLTIFNSQA I IGG++QG+PFQGQ+SGLYYN
Sbjct  412  --------------------------GRQLTIFNSQAAIKIGGRDQGRPFQGQVSGLYYN  513

Query  121  GLKVLNMAAENDANIAIVGNVRLVGEVP  148
            GLKVL +AAE+D N+   G++RLVGE P
Sbjct  514  GLKVLALAAESDPNVRTEGHLRLVGEGP  597


>dbj|BB858994.1| BB858994 RIKEN full-length enriched, brain CRL-1443 BC3H1 cDNA
Mus musculus cDNA clone G430003L02 5', mRNA sequence. Length=460

 Score =   145 bits (365),  Expect = 5e-42, Method: Compositional matrix adjust.
 Identities = 84/142 (59%), Positives = 94/142 (66%), Gaps = 25/142 (18%)
 Frame = +1

Query  1    QGKIGVKFNVGTDDIAIEESNAIINDGKYHVVRFTRSGGNATLQVDSWPVIERYPAGNND  60
            QGKIGVKFNVGTDDIAIEESNAIINDGKYHVVRFTRSGGNATLQVDSWP     P
Sbjct  70   QGKIGVKFNVGTDDIAIEESNAIINDGKYHVVRFTRSGGNATLQVDSWPAYRTLPC----  237

Query  61   NERLAIARQ--RIPYRLGRVVDEWLLD------KGRQLTIFNSQATII-----IGGKEQG  107
                   +Q  R P+RL  +  E+ +D       G      +SQ++I      + GK +
Sbjct  238  ------RKQ**RAPWRL--LDSEFHIDLVE*LMNGYSTKGVSSQSSIAKQP**LAGKSRA  393

Query  108  QPFQGQLSGLYYNGLKVLNMAA  129
             PFQGQLSGLYYNGLKVLNMAA
Sbjct  394  SPFQGQLSGLYYNGLKVLNMAA  459


>gb|DT904887.1| BMSB13606 Hematopoietic stem cells Mus musculus cDNA, mRNA sequence.
Length=721

 Score =   144 bits (364),  Expect = 8e-41, Method: Compositional matrix adjust.
 Identities = 69/69 (100%), Positives = 69/69 (100%), Gaps = 0/69 (0%)
 Frame = +3

Query  4    IGVKFNVGTDDIAIEESNAIINDGKYHVVRFTRSGGNATLQVDSWPVIERYPAGNNDNER  63
            IGVKFNVGTDDIAIEESNAIINDGKYHVVRFTRSGGNATLQVDSWPVIERYPAGNNDNER
Sbjct  513  IGVKFNVGTDDIAIEESNAIINDGKYHVVRFTRSGGNATLQVDSWPVIERYPAGNNDNER  692

Query  64   LAIARQRIP  72
```

```
            LAIARQRIP
Sbjct  693  LAIARQRIP  719


>gb|CX220555.1| MNS36241 Mouse Neurosphere Normalized cDNA library Mus musculus
cDNA 5', mRNA sequence. Length=854

 Score =   100 bits (250),  Expect(2) = 2e-37, Method: Compositional matrix adjust.
 Identities = 60/108 (56%), Positives = 64/108 (59%), Gaps = 31/108 (29%)
 Frame = +1

Query  37   SGGNATLQVDSWPVIERYPAGNNDNERLAIARQRIPYRLGRVVDEWLLDKGRQLTIFNSQ  96
            SGGNATLQVDSWPVIERYPAG                              RQLTIFNSQ
Sbjct  454  SGGNATLQVDSWPVIERYPAG------------------------------RQLTIFNSQ  543

Query  97   ATIIIGGKEQGQPFQGQLSGLYYNGLKVLNMAA-ENDANIAIVGNVRL  143
            ATIIIGGKEQGQPFQGQLSGLYYNG +     + E   +    GNV+L
Sbjct  544  ATIIIGGKEQGQPFQGQLSGLYYNGFESSEYGSRERCQHRHSGGNVKL  687


>gb|CX203319.1| MNS05584 Mouse Neurosphere Normalized cDNA library Mus musculus
cDNA 5', mRNA sequence. Length=645

 Score =   125 bits (313),  Expect = 1e-33, Method: Compositional matrix adjust.
 Identities = 60/66 (91%), Positives = 62/66 (94%), Gaps = 0/66 (0%)
 Frame = +2

Query  34   FTRSGGNATLQVDSWPVIERYPAGNNDNERLAIARQRIPYRLGRVVDEWLLDKGRQLTIF  93
             +RSGGNATLQVDSWPV ERYPAGN DNERLAIARQRIPYRLGRVV+EWLLDKGRQLTIF
Sbjct  446  LSRSGGNATLQVDSWPVNERYPAGNFDNERLAIARQRIPYRLGRVVNEWLLDKGRQLTIF  625

Query  94   NSQATI  99
            NSQA I
Sbjct  626  NSQAAI  643
```

Xenopus (1/9 splice variants)

```
                                                                   Score     E
Sequences producing significant alignments:                       (Bits)  Value

gb|CX806523.1|  JGI_CAAJ17809.fwd NIH_XGC_tropBrn2 Xenopus (Si...    258   1e-84
gb|CX390033.2|  JGI_XZT38012.fwd NIH_XGC_tropTad5 Xenopus (Sil...    254   1e-83
gb|CX842791.2|  JGI_CAAK10710.rev NIH_XGC_tropBrn3 Xenopus (Si...    228   3e-73
gb|CX849796.2|  JGI_CAAL6519.rev NIH_XGC_tropBrn4 Xenopus (Sil...    210   5e-66
gb|EL657246.1|  AGENCOURT_103340710 NICHD_XGC_tropInt_62 Xenop...    210   6e-66
gb|CX875450.1|  JGI_CAAL11566.fwd NIH_XGC_tropBrn4 Xenopus (Si...    203   9e-64
gb|CX802944.2|  JGI_CAAJ15807.rev NIH_XGC_tropBrn2 Xenopus (Si...    200   2e-62
gb|CX849797.1|  JGI_CAAL6519.fwd NIH_XGC_tropBrn4 Xenopus (Sil...    198   3e-61
gb|CX881742.2|  JGI_CAAL20915.rev NIH_XGC_tropBrn4 Xenopus (Si...    137   5e-38
gb|CX806710.2|  JGI_CAAJ17915.rev NIH_XGC_tropBrn2 Xenopus (Si...    111   4e-28
gb|CN079641.1|  EC2BBA16DC06.b1 Xenopus tropicalis xtbs plasmi...    109   2e-27
gb|EL638922.1|  CBTC435.fwd NICHD_XGC_tropBone1 Xenopus (Silur...    101   1e-24
gb|CX879987.2|  JGI_CAAL19921.rev NIH_XGC_tropBrn4 Xenopus (Si...   99.0    6e-24
gb|CX814841.2|  JGI_CAAK651.rev NIH_XGC_tropBrn3 Xenopus (Silu...   99.0    6e-24
gb|CX879988.1|  JGI_CAAL19921.fwd NIH_XGC_tropBrn4 Xenopus (Si...   97.4    5e-23
gb|CX799128.1|  JGI_CAAJ13677.fwd NIH_XGC_tropBrn2 Xenopus (Si...   88.2    9e-20

ALIGNMENTS
>gb|CX806523.1| JGI_CAAJ17809.fwd NIH_XGC_tropBrn2 Xenopus (Silurana) tropicalis
cDNA clone IMAGE:7647663 5', mRNA sequence. Length=839

 Score =   258 bits (658),  Expect = 1e-84, Method: Compositional matrix adjust.
 Identities = 120/148 (81%), Positives = 135/148 (91%), Gaps = 0/148 (0%)
 Frame = +1

Query  1    QGKIGVKFNVGTDDIAIEESNAIINDGKYHVVRFTRSGGNATLQVDSWPVIERYPAGNND  60
            QG +GV FNVGTDDI IEE  A+++DGKYHVVRFTRSGGNATLQVDSWPV ERYPAGN D
Sbjct  118  QGTVGVIFNVGTDDITIEEPGALVSDGKYHVVRFTRSGGNATLQVDSWPVNERYPAGNPD  297

Query  61   NERLAIARQRIPYRLGRVVDEWLLDKGRQLTIFNSQATIIIGGKEQGQPFQGQLSGLYYN  120
            +ERLA+ARQRIPYRLGRVVDEWLLDKGRQLTIFNSQA I +GGK+QG+PFQGQ+SGLYYN
Sbjct  298  SERLALARQRIPYRLGRVVDEWLLDKGRQLTIFNSQAEIKVGGKDQGRPFQGQISGLYYN  477

Query  121  GLKVLNMAAENDANIAIVGNVRLVGEVP  148
            GLKVL++AAEND N+   GN+RL+GEVP
Sbjct  478  GLKVLSLAAENDPNVKTEGNLRLIGEVP  561


>gb|CX390033.2| JGI_XZT38012.fwd NIH_XGC_tropTad5 Xenopus (Silurana) tropicalis
cDNA clone IMAGE:7614026 5', mRNA sequence. Length=818

 Score =   254 bits (650),  Expect = 1e-83, Method: Compositional matrix adjust.
 Identities = 121/135 (90%), Positives = 128/135 (95%), Gaps = 0/135 (0%)
 Frame = +2

Query  14   DIAIEESNAIINDGKYHVVRFTRSGGNATLQVDSWPVIERYPAGNNDNERLAIARQRIPY  73
            DI+IEE NAIINDGKYH VRFTRSGGNATL VD+WPVIERYPAGNNDNERLAIARQRIPY
Sbjct  2    DISIEEVNAIINDGKYHTVRFTRSGGNATLHVDNWPVIERYPAGNNDNERLAIARQRIPY  181

Query  74   RLGRVVDEWLLDKGRQLTIFNSQATIIIGGKEQGQPFQGQLSGLYYNGLKVLNMAAENDA  133
            RLGRVVD+WLLDKGRQLTIFNSQATI IGGK+QGQPFQGQLSGLYYNGLKVLNMAAEN+
Sbjct  182  RLGRVVDDWLLDKGRQLTIFNSQATIKIGGKDQGQPFQGQLSGLYYNGLKVLNMAAENNP  361

Query  134  NIAIVGNVRLVGEVP  148
            +I I GNVRLVG+VP
Sbjct  362  DIVIEGNVRLVGDVP  406
```

```
>gb|CX842791.2| JGI_CAAK10710.rev NIH_XGC_tropBrn3 Xenopus (Silurana) tropicalis
cDNA clone IMAGE:7661344 3', mRNA sequence. Length=831

 Score =   228 bits (581),  Expect = 3e-73, Method: Compositional matrix adjust.
 Identities = 107/119 (90%), Positives = 113/119 (95%), Gaps = 0/119 (0%)
 Frame = -1

Query  30   HVVRFTRSGGNATLQVDSWPVIERYPAGNNDNERLAIARQRIPYRLGRVVDEWLLDKGRQ  89
            H VRFTRSGGNATL VD+WPVIERYPAGNNDNERLAIARQRIPYRLGRVVD+WLLDKGRQ
Sbjct  831  HTVRFTRSGGNATLHVDNWPVIERYPAGNNDNERLAIARQRIPYRLGRVVDDWLLDKGRQ  652

Query  90   LTIFNSQATIIIGGKEQGQPFQGQLSGLYYNGLKVLNMAAENDANIAIVGNVRLVGEVP  148
            LTIFNSQATI IGGK+QGQPFQGQLSGLYYNGLKVLNMAAEN+ +I I GNVRLVG+VP
Sbjct  651  LTIFNSQATIKIGGKDQGQPFQGQLSGLYYNGLKVLNMAAENNPDIVIEGNVRLVGDVP  475


>gb|CX849796.2| JGI_CAAL6519.rev NIH_XGC_tropBrn4 Xenopus (Silurana) tropicalis
cDNA clone IMAGE:7664856 3', mRNA sequence. Length=852

 Score =   210 bits (534),  Expect = 5e-66, Method: Compositional matrix adjust.
 Identities = 100/115 (87%), Positives = 106/115 (92%), Gaps = 0/115 (0%)
 Frame = -1

Query  34   FTRSGGNATLQVDSWPVIERYPAGNNDNERLAIARQRIPYRLGRVVDEWLLDKGRQLTIF  93
            F    GNATL VD+WPVIERYPAGNNDNERLAIARQRIPYRLGRVVD+WLLDKGRQLTIF
Sbjct  810  FYAKWGNATLHVDNWPVIERYPAGNNDNERLAIARQRIPYRLGRVVDDWLLDKGRQLTIF  631

Query  94   NSQATIIIGGKEQGQPFQGQLSGLYYNGLKVLNMAAENDANIAIVGNVRLVGEVP  148
            NSQATI IGGK+QGQPFQGQLSGLYYNGLKVLNMAAEN+ +I I GNVRLVG+VP
Sbjct  630  NSQATIKIGGKDQGQPFQGQLSGLYYNGLKVLNMAAENNPDIVIEGNVRLVGDVP  466


 Score = 36.6 bits (83),  Expect = 0.16, Method: Compositional matrix adjust.
 Identities = 16/18 (89%), Positives = 16/18 (89%), Gaps = 0/18 (0%)
 Frame = -2

Query  21   NAIINDGKYHVVRFTRSG  38
            NAIINDGK H VRFTRSG
Sbjct  848  NAIINDGKSHTVRFTRSG  795


>gb|EL657246.1| AGENCOURT_103340710 NICHD_XGC_tropInt_62 Xenopus (Silurana)
tropicalis cDNA clone IMAGE:8956924 5', mRNA sequence. Length=879

 Score =   210 bits (534),  Expect = 6e-66, Method: Compositional matrix adjust.
 Identities = 100/115 (87%), Positives = 107/115 (93%), Gaps = 0/115 (0%)
 Frame = +1

Query  34   FTRSGGNATLQVDSWPVIERYPAGNNDNERLAIARQRIPYRLGRVVDEWLLDKGRQLTIF  93
            +   GGNATL VD+WPVIERYPAGNNDNERLAIARQRIPYRLGRVVD+WLLDKGRQLTIF
Sbjct  7    YCEVGGNATLHVDNWPVIERYPAGNNDNERLAIARQRIPYRLGRVVDDWLLDKGRQLTIF  186

Query  94   NSQATIIIGGKEQGQPFQGQLSGLYYNGLKVLNMAAENDANIAIVGNVRLVGEVP  148
            NSQATI IGGK+QGQPFQGQLSGLYYNGLKVLNMAAEN+ +I I GNVRLVG+VP
Sbjct  187  NSQATIKIGGKDQGQPFQGQLSGLYYNGLKVLNMAAENNPDIVIEGNVRLVGDVP  351


>gb|CX875450.1| JGI_CAAL11566.fwd NIH_XGC_tropBrn4 Xenopus (Silurana) tropicalis
cDNA clone IMAGE:7669381 5', mRNA sequence. Length=766

 Score =   203 bits (517),  Expect = 9e-64, Method: Compositional matrix adjust.
```

```
 Identities = 105/148 (71%), Positives = 111/148 (75%), Gaps = 30/148 (20%)
 Frame = +3

Query  1    QGKIGVKFNVGTDDIAIEESNAIINDGKYHVVRFTRSGGNATLQVDSWPVIERYPAGNND  60
            QGKIGVKFNVGTDDI+IEE NAIINDGKYH VRFTRSGGNATL VD+WPVIERYPA
Sbjct  3    QGKIGVKFNVGTDDISIEEVNAIINDGKYHTVRFTRSGGNATLHVDNWPVIERYPA----  170

Query  61   NERLAIARQRIPYRLGRVVDEWLLDKGRQLTIFNSQATIIIGGKEQGQPFQGQLSGLYYN  120
                                      GRQLTIFNSQATI IGGK+QGQPFQGQLSGLYYN
Sbjct  171  --------------------------GRQLTIFNSQATIKIGGKDQGQPFQGQLSGLYYN  272

Query  121  GLKVLNMAAENDANIAIVGNVRLVGEVP  148
            GLKVLNMAAEN+ +I I GNVRLVG+VP
Sbjct  273  GLKVLNMAAENNPDIVIEGNVRLVGDVP  356


>gb|CX802944.2| JGI_CAAJ15807.rev NIH_XGC_tropBrn2 Xenopus (Silurana) tropicalis
cDNA clone IMAGE:7646009 3', mRNA sequence. Length=791

 Score =   200 bits (509),  Expect = 2e-62, Method: Compositional matrix adjust.
 Identities = 94/105 (90%), Positives = 100/105 (95%), Gaps = 0/105 (0%)
 Frame = -3

Query  44   QVDSWPVIERYPAGNNDNERLAIARQRIPYRLGRVVDEWLLDKGRQLTIFNSQATIIIGG  103
             VD+WPVIERYPAGNNDNERLAIARQRIPYRLGRVVD+WLLDKGRQLTIFNSQATI IGG
Sbjct  789  HVDNWPVIERYPAGNNDNERLAIARQRIPYRLGRVVDDWLLDKGRQLTIFNSQATIKIGG  610

Query  104  KEQGQPFQGQLSGLYYNGLKVLNMAAENDANIAIVGNVRLVGEVP  148
            K+QGQPFQGQLSGLYYNGLKVLNMAAEN+ +I I GNVRLVG+VP
Sbjct  609  KDQGQPFQGQLSGLYYNGLKVLNMAAENNPDIVIEGNVRLVGDVP  475


>gb|CX849797.1| JGI_CAAL6519.fwd NIH_XGC_tropBrn4 Xenopus (Silurana) tropicalis
cDNA clone IMAGE:7664856 5', mRNA sequence. Length=922

 Score =   198 bits (504),  Expect = 3e-61, Method: Compositional matrix adjust.
 Identities = 95/107 (89%), Positives = 101/107 (94%), Gaps = 0/107 (0%)
 Frame = +1

Query  1    QGKIGVKFNVGTDDIAIEESNAIINDGKYHVVRFTRSGGNATLQVDSWPVIERYPAGNND  60
            QGKIGVKFNVGTDDI+IEE NAIINDGKYH VRFTRSGGNATL VD+WPVIERYPAG ND
Sbjct  565  QGKIGVKFNVGTDDISIEEVNAIINDGKYHTVRFTRSGGNATLHVDNWPVIERYPAGXND  744

Query  61   NERLAIARQRIPYRLGRVVDEWLLDKGRQLTIFNSQATIIIGGKEQG  107
            NERLAIARQRIPYRLGRVVD+WLLDKGRQLTIF+SQ+TI IGG+ QG
Sbjct  745  NERLAIARQRIPYRLGRVVDDWLLDKGRQLTIFHSQSTIKIGGEGQG  885


>gb|CX881742.2| JGI_CAAL20915.rev NIH_XGC_tropBrn4 Xenopus (Silurana) tropicalis
cDNA clone IMAGE:7672727 3', mRNA sequence. Length=846

 Score =   137 bits (346),  Expect = 5e-38, Method: Compositional matrix adjust.
 Identities = 65/75 (87%), Positives = 70/75 (93%), Gaps = 0/75 (0%)
 Frame = -1

Query  74   RLGRVVDEWLLDKGRQLTIFNSQATIIIGGKEQGQPFQGQLSGLYYNGLKVLNMAAENDA  133
            RLGRVVD+WLLDKGRQLTIFN QATI IGGK+QGQPFQGQLSGLYYNGLKVLNMAAEN+
Sbjct  846  RLGRVVDDWLLDKGRQLTIFNXQATIKIGGKDQGQPFQGQLSGLYYNGLKVLNMAAENNP  667

Query  134  NIAIVGNVRLVGEVP  148
            +I I GNVRLVG+VP
Sbjct  666  DIVIEGNVRLVGDVP  622
```

Danio rerio (1/6 splice variants)

```
                                                                 Score     E
Sequences producing significant alignments:                      (Bits)  Value

gb|CN505896.1| AGENCOURT_22422788 NIH_ZGC_9 Danio rerio cDNA ...    230    9e-74
gb|EB940169.1| 9541915 ZF34 Danio rerio cDNA clone 3410906, m...    205    2e-65
gb|DN896482.1| nap10b06.y1 Zebrafish anterior segment (minus ...    169    8e-51
gb|EG574210.1| AGENCOURT_88150324 NIH_ZGC_26 Danio rerio cDNA...    152    5e-44
gb|EB962033.1| 14363019 ZF35XL Danio rerio cDNA clone 3866436...    128    8e-37
gb|EB961180.1| 14363301 ZF35XL Danio rerio cDNA clone 3866772...    111    6e-30
gb|EB923101.1|  12401429 ZF31 Danio rerio cDNA clone 3275619, ...  59.3    4e-10
emb|AL914040.1|  AL914040 PJR-Z1+Z2 Danio rerio cDNA clone 088...  34.7    0.34


ALIGNMENTS
>gb|CN505896.1| AGENCOURT_22422788 NIH_ZGC_9 Danio rerio cDNA clone IMAGE:7275113
5', mRNA sequence. Length=947

 Score =   230 bits (587),  Expect = 9e-74, Method: Compositional matrix adjust.
 Identities = 110/148 (74%), Positives = 127/148 (86%), Gaps = 0/148 (0%)
 Frame = +3

Query  1    QGKIGVKFNVGTDDIAIEESNAIINDGKYHVVRFTRSGGNATLQVDSWPVIERYPAGNND  60
            QGK+GV FNVGTDDI I+E    +NDGKYHVVRFTRSGGNATLQVD+ PVIER+P+GN D
Sbjct  330  QGKVGVIFNVGTDDITIDEPAVTVNDGKYHVVRFTRSGGNATLQVDNQPVIERFPSGNID  509

Query  61   NERLAIARQRIPYRLGRVVDEWLLDKGRQLTIFNSQATIIIGGKEQGQPFQGQLSGLYYN  120
            NERLAIARQRIPYRLGRVVDEWLLDKGRQLTIFNSQA I +GG+++G+PF GQ+S LYYN
Sbjct  510  NERLAIARQRIPYRLGRVVDEWLLDKGRQLTIFNSQAAIKVGGQDKGRPFPGQISCLYYN  689

Query  121  GLKVLNMAAENDANIAIVGNVRLVGEVP  148
            GL+VL +AAE + N+ + G   LVGE P
Sbjct  690  GLQVLKLAAEGEPNVQVAGIFPLVGEPP  773


>gb|EB940169.1| 9541915 ZF34 Danio rerio cDNA clone 3410906, mRNA sequence.
Length=620

 Score =   205 bits (522),  Expect = 2e-65, Method: Compositional matrix adjust.
 Identities = 94/141 (67%), Positives = 120/141 (85%), Gaps = 0/141 (0%)
 Frame = +1

Query  1    QGKIGVKFNVGTDDIAIEESNAIINDGKYHVVRFTRSGGNATLQVDSWPVIERYPAGNND  60
            QGKIGV FN+GT DI ++ES+  +NDGKYHVVRFTR+GGNATLQVD+W + E +P+GN+D
Sbjct  196  QGKIGVTFNIGTADITVQESSTAVNDGKYHVVRFTRNGGNATLQVDNWAINEHFPSGNSD  375

Query  61   NERLAIARQRIPYRLGRVVDEWLLDKGRQLTIFNSQATIIIGGKEQGQPFQGQLSGLYYN  120
            NER+ +A ++IP++  R V+EWL +KGRQLTIFN+QATI IGG ++ +PFQGQLSGLYYN
Sbjct  376  NERIQMANKKIPFKYARPVEEWLQEKGRQLTIFNTQATITIGGSDRKRPFQGQLSGLYYN  555

Query  121  GLKVLNMAAENDANIAIVGNV  141
            GLKVLNMAA+ + NI I G+V
Sbjct  556  GLKVLNMAAQGNPNIKINGSV  618


>gb|DN896482.1| nap10b06.y1 Zebrafish anterior segment (minus lens). Unnormalized
(nap) Danio rerio cDNA clone nap10b06 5', mRNA sequence. Length=697

 Score =   169 bits (427),  Expect = 8e-51, Method: Compositional matrix adjust.
 Identities = 89/149 (60%), Positives = 102/149 (68%), Gaps = 31/149 (21%)
 Frame = +3
```

```
Query  1    QGKIGVKFNVGTDDIAIEESNAIINDGKYHVVRFTRSGGNATLQVDSWPVIERYPAGNND  60
            +G I V FNVGTDDI IEE++  +NDGKYH+VRFTRSGGNATLQVD  PVIERYP+
Sbjct  120  RGNIKVVFNVGTDDINIEETSKFVNDGKYHIVRFTRSGGNATLQVDDLPVIERYPS----  287

Query  61   NERLAIARQRIPYRLGRVVDEWLLDKGRQLTIFNSQATIIIGGKEQG-QPFQGQLSGLYY  119
                                      GRQLTIFNSQ TI IGG E+G +PFQGQLSGLYY
Sbjct  288  --------------------------GRQLTIFNSQTTIKIGGWEKGSRPFQGQLSGLYY  389

Query  120  NGLKVLNMAAENDANIAIVGNVRLVGEVP  148
            NGLKVLNMAAE D N+ + G+ RLVG++P
Sbjct  390  NGLKVLNMAAEGDPNVRVEGSARLVGDMP  476
```

>gb|EG574210.1| AGENCOURT_88150324 NIH_ZGC_26 Danio rerio cDNA clone IMAGE:8766761 5', mRNA sequence. Length=829

```
 Score =   152 bits (385),  Expect = 5e-44, Method: Compositional matrix adjust.
 Identities = 71/86 (83%), Positives = 80/86 (93%), Gaps = 0/86 (0%)
 Frame = +3

Query  1    QGKIGVKFNVGTDDIAIEESNAIINDGKYHVVRFTRSGGNATLQVDSWPVIERYPAGNND  60
            +G++GV FNVGTDDI IEES  +++DGKYHVVRFTRSGGNATLQVD+ PVIER+PAGN D
Sbjct  420  RGRVGVIFNVGTDDIVIEESGVMVSDGKYHVVRFTRSGGNATLQVDNLPVIERFPAGNFD  599

Query  61   NERLAIARQRIPYRLGRVVDEWLLDK  86
            +ERLAIARQRIPYRLGRVVDEWLLDK
Sbjct  600  SERLAIARQRIPYRLGRVVDEWLLDK  677
```

>gb|EB962033.1| 14363019 ZF35XL Danio rerio cDNA clone 3866436, mRNA sequence. Length=247

```
 Score =   128 bits (322),  Expect = 8e-37, Method: Compositional matrix adjust.
 Identities = 58/78 (74%), Positives = 70/78 (90%), Gaps = 0/78 (0%)
 Frame = +1

Query  71   IPYRLGRVVDEWLLDKGRQLTIFNSQATIIIGGKEQGQPFQGQLSGLYYNGLKVLNMAAE  130
            IPYRLGRVVDEWLLDKGRQLTIFNSQA I +GG+++G+PFQGQ+SGLYYNGL+VL +AAE
Sbjct  1    IPYRLGRVVDEWLLDKGRQLTIFNSQAAIKVGGQDKGRPFQGQISGLYYNGLQVLKLAAE  180

Query  131  NDANIAIVGNVRLVGEVP  148
             D N+ + GN+RLVG+ P
Sbjct  181  GDPNVQVAGNLRLVGDAP  234
```

>gb|EB961180.1| 14363301 ZF35XL Danio rerio cDNA clone 3866772, mRNA sequence. Length=318

```
 Score =   111 bits (277),  Expect = 6e-30, Method: Compositional matrix adjust.
 Identities = 47/77 (61%), Positives = 64/77 (83%), Gaps = 0/77 (0%)
 Frame = +2

Query  1    QGKIGVKFNVGTDDIAIEESNAIINDGKYHVVRFTRSGGNATLQVDSWPVIERYPAGNND  60
            QGKIGV FN+GT DI ++ES+  +NDGKYHVVRFTR+GGNATLQVD+W + E +P+ N+D
Sbjct  83   QGKIGVTFNIGTADITVQESSTAVNDGKYHVVRFTRNGGNATLQVDNWAINEHFPSXNSD  262

Query  61   NERLAIARQRIPYRLGR  77
            NER+ +A ++IP++  R
Sbjct  263  NERIQMANKKIPFQYAR  313
```

CIONA (0/2 splice variants)

```
Sequences producing significant alignments:                      (Bits)  Value

dbj|AV990413.1|  AV990413 Nori Satoh unpublished cDNA library,...    102   3e-25
dbj|BW351510.1|  BW351510 Yutaka Satou unpublished cDNA librar...  92.0    1e-21
dbj|AV854354.1|  AV854354 Nori Satoh unpublished cDNA library,...  31.6    2.6
dbj|AV854371.1|  AV854371 Nori Satoh unpublished cDNA library,...  31.6    3.7

>dbj|AV990413.1| AV990413 Nori Satoh unpublished cDNA library, larva Ciona
intestinalis
cDNA clone cilv22i22 5', mRNA sequence. Length=692

 Score =   102 bits (253),  Expect = 3e-25, Method: Compositional matrix adjust.
 Identities = 54/156 (35%), Positives = 88/156 (56%), Gaps = 19/156 (12%)
 Frame = +1

Query  8    FNVGTDDIAIEESNAIINDGKYHVVRFTRSGGNATLQVDSWPVIERYPAGNNDNERLAIA  67
            FN+G  D+ ++E ++ ++DG+YH VR  R   NATL+VD+WP       G +  +R A+A
Sbjct  25   FNIGNSDVKLQE-HSPVSDGQYHTVRVRRKWMNATLRVDNWPERTATTGGS-RDRFALA  198

Query  68   RQRIPYRLGRVVDEWLLDKGRQLTIFNSQATIIIGGK-EQGQP----------------F  110
            +  IP  +   V+ WL  K    T+FNSQ+ I IGG  +Q  P                F
Sbjct  199  KPSIPTSVLSTVETWLRSKEYSNTVFNSQSVIQIGGLLDQSSPYLNSMRSKRSPGIRKAF  378

Query  111  QGQLSGLYYNGLKVLNMAAENDANIAIVGNVRLVGE  146
             GQ+ G ++N ++V  +AA+ D ++ ++G+V ++GE
Sbjct  379  HGQMMGFFFNDVRVFQLAAQGDPSVQLIGDVEIIGE  486


>dbj|BW351510.1| BW351510 Yutaka Satou unpublished cDNA library, embryo whole
animal Ciona intestinalis cDNA clone ciem848m04 5', mRNA sequence. Length=623

 Score = 92.0 bits (227),  Expect = 1e-21, Method: Compositional matrix adjust.
 Identities = 49/139 (35%), Positives = 76/139 (55%), Gaps = 18/139 (13%)
 Frame = +1

Query  25   NDGKYHVVRFTRSGGNATLQVDSWPVIERYPAGNNDNERLAIARQRIPYRLGRVVDEWLL  84
            +DG+YH VR  R   NATL+VD+WP       G +  +R A+A+  IP  +   V+ WL
Sbjct  1    SDGQYHTVRVRRKWMNATLRVDNWPERTATTGGS-RDRFALAKPSIPTSVLSTVETWLR  177

Query  85   DKGRQLTIFNSQATIIIGGK-EQGQP----------------FQGQLSGLYYNGLKVLNM  127
             K    T+FNSQ+ I IGG  +Q  P                F GQ+ G ++N ++V  +
Sbjct  178  SKEYSNTVFNSQSVIQIGGLLDQSSPYLNSMRSKRSPGIRKAFHGQMMGFFFNDVRVFQL  357

Query  128  AAENDANIAIVGNVRLVGE  146
            AA+ D ++ ++G+V ++GE
Sbjct  358  AAQGDPSVQLIGDVEIIGE  414
```
